# Supplementary material for: Re-evaluating Phoneme Frequencies
Source: Front Psychol. 2020 Nov 20;11:570895. doi: 10.3389/fpsyg.2020.570895 (PMC7714923; doi:10.3389/fpsyg.2020.570895)
Supplement: Supplementary file 1 [file Data_Sheet_1.PDF]

Supplementary information

Re-evaluating phoneme frequencies

Supplementary information and materials for this study consist of seven parts. This document contains five sections of text-based supplementary information:

- S1: Neutral expectations about phonemes' lexical and discourse frequencies
- S2: Guide to data and code
- S3: Original wordlist sources
- S4: Comparison of phoneme inventories in Ausphon and Phoible
- S5: Tables of results

In addition, the following supplementary materials are downloadable from http://doi.org/10.5281/zenodo.4104116:

- S6: Data viewer
- S7: Data and code

S1 discusses the neutral expectations about lexical and discourse frequencies described briefly in the Data section of the main paper. S2, as the name suggests, is a user guide to the various files and directories in S6 and S7. S3 gives bibliographic details for the original wordlists used in the study. S4 compares phonemes inventories in our Ausphon data to inventories in the corresponding Australian languages recorded in Phoible (Moran & McCloy 2019, Round 2019). S5 gives extended tables of results, listing maximum likelihood estimates of parameters, goodness-of-fit statistics and p values for each language and distribution type.

S6 is found in the downloadable directory `S6_data_viewer`. This directory contains an interactive app for visualizing phoneme frequencies in each language, the fit of different distributions, and uncertainty in our results. It was not used at any stage of analysis. The intention is to give a convenient tool for viewing plots in any given language, rather than simply presenting a list of plots for all 166 languages in a long, unwieldy document.

S7 is comprised of the remainder of the material downloadable from http://doi.org/10.5281/zenodo.4104116. This contains all the code and data files needed to replicate the study and the original output we produced saved in an Rdata format. For usage details, consult the guide in S2 below.

S1. Neutral expectations about lexical and discourse frequencies

In the Data section of the main paper we briefly discussed the fact that the neutral expectation is that lexical phoneme frequencies and discourse phoneme frequencies will have comparable distributions, despite that fact that words in discourse themselves have a Zipfian distribution. Since this result can be counter-intuitive at first, here we provide a more extended discussion of why it is so. There is some light mathematics involved, but the aim here has been to keep the discussion at an accessible level.

To aid our discussion of quantitative frequencies, it is helpful to begin with some definitions. Firstly, for raw numbers of phonemes and words:

- Our phoneme inventory consists of n phonemes, designated p_i for $i \in 1, 2, \ldots, n$. Throughout this discussion, we will use the subscript i for phonemes.
- Our lexicon consists of m words, designated w_j for $j \in 1, 2, \ldots, m$. Throughout this discussion, we will use subscript j for words.
- Designate as t_{ij} the number of tokens of phoneme p_i in word w_j.
  - Similarly, designate as t_i the number of tokens of phoneme p_i in *all* words, i.e., $t_i = \sum_{j=1}^{m} t_{ij}$.
  - Designate as t_j the number of tokens *all* phonemes in word w_j, i.e., $t_j = \sum_{i=1}^{n} t_{ij}$.
  - And designate simply as t the total number of tokens *all* phonemes in *all* words, i.e., $t = \sum_{i=1}^{n} \sum_{j=1}^{m} t_{ij} = \sum_{i=1}^{n} t_i = \sum_{j=1}^{m} t_j$.

We can now define the lexical frequency of our phonemes:

- Designate as l_i the (relative) lexical frequency of phoneme p_i, namely $l_i = t_i/t$. Note that these lexical frequencies have been defined so they sum to 1:

$$\sum_{i=1}^{n} l_i = \sum_{i=1}^{n} (t_i/t) = \frac{1}{t} \sum_{i=1}^{n} t_i = \frac{1}{t} t = 1$$

Next, we need the discourse frequency of words:

- Designate as f_j the (relative) discourse frequency of word w_j, defined so that $\sum_{j=1}^{m} f_j = 1$.

And from that we can derive discourse frequencies of phonemes.

- Designate as R the discourse 'rate' of phonemes, i.e., the mean number of phonemes per word in discourse, which is given as the sum of the length (in phonemes) of each word, weighted by that word's discourse frequency. i.e., $R = \sum_{j=1}^{m} (t_j \cdot f_j)$.
- Designate as d_i the (relative) discourse frequency of a specific phoneme p_i. This will be given by $d_i = \sum_{j=1}^{m} (t_{ij} \cdot f_j)/R$. Note that these sum to 1:

$$\sum_{i=1}^{n} d_i = \sum_{i=1}^{n} \sum_{j=1}^{m} (t_{ij} \cdot f_j)/R = \frac{1}{R} \sum_{i=1}^{n} \sum_{j=1}^{m} (t_{ij} \cdot f_j) = \frac{1}{R} \sum_{j=1}^{m} (f_j \cdot \sum_{i=1}^{n} t_{ij}) = \frac{1}{R} \sum_{j=1}^{m} (f_j \cdot t_j) = \frac{1}{R} R = 1$$

Thought experiment #1

To begin our first thought experiment, suppose that our language contains $m = 10,000$ lexemes. Each lexeme is 6 phonemes long, so $t_j = 6, j \in 1, 2, \ldots, m$. Trivially, in this situation, the discourse rate of phonemes (the mean number of phonemes per word in discourse), R, will also be 6. And the total number of phoneme tokens in the lexicon, t, is $6 \cdot m$, or 60,000.

As a general fact owing to how the lexical frequency l_i of a phoneme p_i is defined, if we examine any randomly chosen word w_j and then any randomly chosen phoneme token within it, then the chance of that token being of phoneme type p_i (for any i) is l_i. Recall also that $l_i = t_i/t$. Since $t = 6 \cdot m$ this means that in this thought experiment, $l_i = t_i/6m$

Next, suppose that every word w_j has exactly the same discourse frequency, f_j. Since all the words' discourse frequencies sum to 1 and since there are m words, this means that $f_j = \frac{1}{m}, j \in 1, 2, \ldots, m$.

When every word has the same discourse frequency, this means that every phoneme p_i has a discourse frequency d_i which is equal to its lexical frequency l_i. We can show this as follows:

$$d_i = \sum_{j=1}^{m}(t_{ij} \cdot f_j)/R = \frac{1}{6}\sum_{j=1}^{m}(t_{ij} \cdot f_j) = \frac{1}{6}\sum_{j=1}^{m}(t_{ij} \cdot \frac{1}{m}) = \frac{1}{6m}\sum_{j=1}^{m} t_{ij} = \frac{1}{6m} t_i = l_i$$

So, in this first thought experiment, we see that discourse frequencies and lexical frequencies are exactly equal.

Thought experiment #2

In the second thought experiment, we begin with the language that we set up in thought experiment #1. We then increase by 50% the rate at which half of the words occur in discourse and decrease by 50% the rate at which the other half occur. This means that now $f_j = \frac{1.5}{m}, j \in 1, 2, \ldots, 5000$ and $f_j = \frac{0.5}{m}, j \in 5001, 5002, \ldots, 10000$. Because in this thought experiment every word still contains 6 phonemes, it is still the case that R, the discourse rate of phonemes, is 6.

Now the question is, what happens to phonemes' discourse rates? For the phonemes lucky enough to be in w_1 for example, their discourse frequencies will be boosted by a small amount. For the phonemes unlucky enough to be in w_{5001}, their discourse frequencies will be reduced. But notice, that when dividing the words into those whose discourse frequencies we increased versus those whose discourse frequencies we decreased, we selected the words blindly, and not on the basis of *which* phonemes they contained. Consequently, any given phoneme is just as likely to occur in word w_1 (whose discourse frequency was increased by an amount of $\frac{0.5}{m}$) as it is to occur in word w_{5001} (whose discourse frequency was decreased by precisely the same amount, $\frac{0.5}{m}$). Thus although we have significantly changed the discourse frequencies of the *words*, it remains the case that any given phoneme is just as likely to have had its discourse frequency increased as decreased; so *on average* its change is 0. Consequently, the discourse frequency of any given phoneme *on average* stays the same as it was in thought experiment #1, i.e., it is equal to the phoneme's lexical frequency l_i.

So, in this second thought experiment, we see that even if the discourse frequency of words is changed or skewed, our neutral expectation for the discourse frequency of any given phoneme p_i is still $d_i = l_i$.

Thought experiment #3

Although we have confirmed, in a lexicon where all words are the same length, that our neutral expectation for the discourse frequency of any given phoneme p_i remains $d_i = l_i$, we have not yet considered a lexicon where words have different lengths, and more to the point, one in which shorter words are more frequent, as we know them to be, on average, in real languages. So, in thought experiment #3, suppose that half the words in the lexicon ($w_1, w_2, \ldots, w_{5000}$) contain not 6 but 4 phonemes, and the other half ($w_{5001}, w_{5002}, \ldots, w_{10000}$) contain not 6 but 8. Like in thought experiment #2, we assume that the first 5000 words (all the shorter ones) have a high discourse frequency $f_j = \frac{1.5}{m}, j \in 1, 2, \ldots, 5000$ and the other half (all the longer ones) have a low discourse frequency $f_j = \frac{0.5}{m}, j \in 5001, 5002, \ldots, 10000$. Before we ask what happens to the phoneme frequencies, we should note that because shorter words are now more frequent, there is a change in the discourse rate of phonemes, R. In discourse, the frequency of shorter words, which comprise half of the lexicon and each contain 4 phonemes, is $\frac{1.5}{m}$ while the frequency of longer words, which comprise half of the lexicon and each contain 8 phonemes is $\frac{0.5}{m}$. Consequently the average length in phonemes of any word in discourse is $R = (\frac{1}{2} \times 4 \times \frac{1.5}{m} + \frac{1}{2} \times 8 \times \frac{0.5}{m})/m = 5$, or 5/6 of what it was in thought experiments #1 and #2.

Next we should consider the lexical distribution of phonemes. Half of the lexicon is now comprised of words containing 4 phonemes, while half is comprised of words that are twice as long, containing 8. Consequently, just one third of all phoneme tokens are situated in the 'short' words, and two thirds of them are in the 'long' words. Similarly, for any specific phoneme p_i, on average, one third of its tokens will be in short words and two thirds of them will be in long words. We should notice again, that in this experiment, when dividing the lexicon into shorter and longer words, we selected the words blindly. We did not, for example, say that short words would be more or less likely to contain any specific phonemes.

To understand the implications for discourse frequencies of phonemes, we can use thought experiment #1 as a baseline. In #1, all words had the same discourse frequency, $\frac{1}{m}$, and phonemes' tokens were equally distributed among them. In the current thought experiment, on average, 1/3 of a phonemes' tokens are in short words whose discourse frequencies are 1.5 times as great, at $\frac{1.5}{m}$, while 2/3 of a phonemes' tokens are in long words whose discourse frequencies are 0.5 times as great, at $\frac{0.5}{m}$. On average then, the rate at which phonemes appear in discourse has changed by a factor of $(\frac{1}{3} \times 1.5 + \frac{2}{3} \times 0.5) = \frac{2.5}{3} = 5/6$, or exactly what we noted was the overall change in R. Since each phoneme's rate of occurrence has changed on average exactly as much as the overall discourse rate of phonemes, its relative discourse frequency d_i remains unchanged from our baseline (thought experiment #1), in which $d_i = l_i$.

So, in this third thought experiment, we see that even if the discourse frequency of words is changed or skewed *and* words have unequal lengths *and* their lengths correlate with their discourse frequencies, our neutral expectation for the discourse frequency of any given phoneme p_i is still $d_i = l_i$.

Discussion

We have seen that even as *words* are given different discourse frequencies, and as their lengths are changed, our neutral expectation for the discourse frequencies of *phonemes*, d_i, remains equal to their lexical frequencies l_i. This finding can be dissected a little further. Firstly, the result assumes that differences in words' discourse frequencies are *independent* of their phonemic content. Secondly, it assumes that differences in words' lengths are likewise independent of their phonemic content. Were either of these assumptions not to hold, then our expectation will potentially change. Furthermore, when we state that 'the neutral expectation for the discourse frequencies d_i, remains equal to their lexical frequencies l_i', we are not stating that we expect that $d_i = l_i$ for each i. On the contrary, we expect that there will be some difference due to normal, statistical fluctuations, which we can designate $\delta_i = (d_i - l_i)$, but that because δ_i can be both positive or negative, the values of δ_i overall cancel out. We can show this, using the fact that our frequencies d_i and l_i have been defined such that they always sum to 1, i.e., $\sum_{i=1}^{n} d_i = 1$ and $\sum_{i=1}^{n} l_i = 1$:

$$\sum_{i=1}^{n} \delta_i = \sum_{i=1}^{n} (d_i - l_i) = \sum_{i=1}^{n} d_i - \sum_{i=1}^{n} l_i = 1 - 1 = 0$$

And if the sum of δ_i is 0, then so too is the mean of δ_i: the average difference between discourse and lexical frequencies is zero. As we have already noted, this is not to say that the *magnitude* of each δ_i is expected to be zero. On the contrary, specific values of d_i and l_i will differ and consequently we can think of any *distribution* of phonemic discourse frequencies d_i as being a 'noisy' reflection of the distribution of phonemic lexical frequencies l_i, and vice-versa. Nevertheless though, beyond that random noise, there is no systematic, biased difference expected (provided our assumptions of independence hold true as mentioned above).

S2. Guide to data and code

S6 and S7 are downloadable from http://doi.org/10.5281/zenodo.4104116. This directory contains a data viewer app, original data files and code for replicating the study, plus the original results files generated as output for the paper. Within these materials, the *S6_data_viewer* directory contains the data viewer app. The *S7_data_code_results* directory contains three subdirectories, `data`, `R` and `results`. Each of these are described in turn below.

Guide to Data Viewer

S6 is a Shiny app hosted on Github at [[https://github.com/JaydenM-C/phonfreq]]. It is also downloadable directly from *S6_data_viewer* at http://doi.org/10.5281/zenodo.4104116. The main feature of this repository is an interactive app for visualizing phoneme frequency data for each language. The app was produced in R, using the package *shiny* (Chang et al. 2018). In addition, the app plots lines corresponding to the fit of each candidate distribution and plots the results of each bootstrapping procedure (including approximate 95% confidence intervals for each parameter). The best and easiest way to use the app is to download it as an R package with the following command:

If the `devtools` package is not installed, uncomment the first line to install it first.

Once the package is installed, the data viewer app can be opened with the following command:

Alternatively, to run the app from the *S6_data_viewer* directory, open the **app.R** file in Rstudio and click 'Run App'. If using the regular R console, set the working directory to the location of **app.R** and run the command 'shiny::runApp()'. The app will need to access two other directories, *data* and *results* which are also downloadable at http://doi.org/10.5281/zenodo.4104116.

Data files

The `data` directory contains two data spreadsheets in tab-separated format, **Aus_segment_frequencies_2020-10-13.tsv** and **Aus_metadata_2020-10-13.tsv**. The first of these contains frequency data extracted from the Ausphon-Lexicon database. Each row corresponds to one unique phonological segment (identified in the 'match' column). The 'lex_ID' contains a Ausphon-Lexicon's numerical identifier for each language variety. The corresponding common name of the language variety is listed in the 'variety_name' column. The 'count' column contains the segment's raw count in the language's wordlist, 'freq' gives the segment's frequency expressed as a fraction of the total number of segments in the wordlist. 'n_forms' is the number of words in the language's lexicon.

The second file, **Aus_metadata_2020-10-13.tsv**, is a simple key matching Ausphon-Lexicon ID numbers to language variety names.

Code

The *R* directory contains the R scripts used to conduct the analyses in the study. Analysis was performed with the script **Aus_powerlaw.R**, which is called with a single parameter giving the index of the language to be analyses (between 1 and 166). The script was submitted as a job batch to the Awoonga high performance computer cluster at The University of Queensland, Australia. The Awoonga system has 40 nodes, each consisting of 24 cores (Intel(R) Xeon(R) CPU E5-2670 v3 @ 2.30GHz), with 256 GB of RAM and 300 GB of disk memory, interconnected by a 10 Gigabit Ethernet network. The operating system is CentOS version 7 and batch jobs are submitted via PBS Pro software. A script **compile_MLE_results.R** compiles the many small results file generated by **Aus_powerlaw.R** into the main results files described in the next section. The script **Vuongs_LRT.R** performs Vuong's likelihood ratio tests. The script **create_figs_FrontPsychol.R** generates the figures that appear in the paper.

The first three R scripts are dependent on two R packages, *tidyverse* and *poweRlaw*. To plot figures we also used the packages *cowplot*, *kableExtra*, *extrafont*, *ggrepel* and *Cairo*, and the fontface *Noto Sans*. These packages are available on CRAN (cran.r-project.org) and if they are not already installed on the user's machine, they can be installed via the standard `install.packages` command in the R console. The scripts

are also dependent on the `data` and `results` directories. As long as these requirements are satisfied, any user should be able to open and run the scripts in R software to reproduce the study's analysis and results. Just be aware that the bootstrapping procedures are computationally intensive and may take a long time (i.e. several days) to run on a personal machine.

Results files

The script **Aus_powerlaw.R** initially produces four results files for each individual language, stored in Rdata format in an `individual_results` directory. The script **compile_MLE_results.R** compiles these into four main results files in Rdata format, saved in the `results` directory. Each of those Rdata files contain four list objects. The four lists are (replacing * with one of 'pl', 'lnorm', 'exp' or 'pois'):

aus_dis*: A list of length 166, where each element in the list is a 'dis*' object (defined in the *poweRlaw* package)—one for each language variety in our language sample. Each element in the list is named with the applicable Ausphon-Lexicon lex_ID. Each dis* object contains the maximum likelihood estimates of the parameters for a given distribution for a particular language. In other words, we use the *poweRlaw* package to fit a particular distribution to the segment frequencies of each language and save the results for each language as an element of a list. The 'dis*' object classes contain a variety of other information, such as a copy of all data points and information on the package version used—see the documentation for the *poweRlaw* package for details.

aus_dis*_xmin: A list of length 166, exactly the same as above, except an x_{min} parameter is estimated first using maximum likelihood and then the distribution is fitted to the remaining frequency values equal to or above x_{min}.

aus_bootstrap_*: A list of length 166, as above. This time, each element of the list is a 'dis*' object which is the result of running the bootstrap procedure (using the 'bootstrap_p' function in the *poweRlaw* package). Each 'dis*' object includes the output of each individual bootstrap iteration.

aus_bootstrap_*_xmin: As above, but with the x_{min} parameter included.

The script **Vuongs_LRT.R** produces one results file in Rdata format, saved in the *results* directory. This file, **Aus_Voungs_LRT.Rdata** contains results of our seven pairwise likelihood ratio test, in seven objects. One, named **lrt_results_e_l**, is the comparison of the exponential and lognormal distributions without x_{min}, and six named **lrt_results_xmin_*a*_*b*** where a and b are each possible combination of e, l and p, comparing the exponential, lognormal and power law distributions using the x_{min} parameter estimated for the a distribution.

S3. Original wordlist sources

Adnyamathanha

CHIRILA source: CHIRILA/v2/McEnteeMcKenzie

McEntee, John & Pearl McKenzie. 1992. *Adna-mat-na English dictionary.* Adelaide: the authors. 125 pp.

Phonemic normalization: Coda tap normalized as vibrant. Otherwise, voiced stops, taps and fricatives normalized to lenis obstruents.

Alawa

Sharpe, Margaret C. 2001. *Alawa nanggaya nindanya yalanu junggulu = Alawa-Kriol-English dictionary.* Prospect, South Australia: Caitlin Press. 245 pp.

Amurdak

Handelsmann, Robert. 1991. *Towards a description of Amurdak: A language of northern Australia.* Melbourne: University of Melbourne Honours Thesis

Phonemic normalization: Prelateralized stops normalized as flapped laterals.

Angkamuthi

Crowley, Terry. 1983. Uradhi. In R. M. W. Dixon & Barry J. Blake (eds.), *Handbook of Australian languages*, vol. 3, 5 vols., 307–428. Amsterdam: John Benjamins

Anguthimri

CHIRILA source: CHIRILA/v1/ASEDA0240

Crowley, Terry. 1989. Mbakwithi vocabulary. Australian Institute of Aboriginal and Torres Strait Islander Studies, Australian Indigenous Languages Collection. ASEDA 0240. Canberra

Atampaya

Crowley, Terry. 1983. Uradhi. In R. M. W. Dixon & Barry J. Blake (eds.), *Handbook of Australian languages*, vol. 3, 5 vols., 307–428. Amsterdam: John Benjamins

Badimaya

Marmion, Doug. 1995. Badimaya dictionary. Australian Institute of Aboriginal and Torres Strait Islander Studies, Australian Indigenous Languages Collection. ASEDA 0615. Canberra

Phonemic normalization: Double a normalized to long vowel.

Bakanh

Hamilton, Philip. 1997a. *Pakanh Alphabetical Search Index.* Oykangand and Olkola Dictionary. http://www.oocities.org/athens/delphi/2970/pakalpha.htm

Bardi

CHIRILA source: CHIRILA/v1/akl99

Aklif, Gedda & Kimberley Language Resource Centre. 1999. *Ardiyooloon Bardi Ngaanka: One Arm Point Bardi dictionary.* Halls Creek, WA, Australia: Kimberley Language Resource Centre. 222 pp.

Bidyara

Breen, Gavan. 1973. *Bidyara and Gungabula grammar and vocabulary.* Vol. 8 (Linguistic Communications). Melbourne: Monash University. 227 pp.

Bilinarra

Meakins, Felicity, Lauren Campbell, et al. 2013. *Bilinarra to English dictionary.* Batchelor, NT, Australia: Batchelor Press. 264 pp.

Biri

CHIRILA source: CHIRILA/v1/Terrell

Terrill, Angela. 1999. Biri lexicons. Australian Institute of Aboriginal and Torres Strait Islander Studies, Australian Indigenous Languages Collection. ASEDA 0700. Canberra. http://aiatsis.gov.au/sites/default/files/catalogue_resources/0700_access.zip

Bularnu

Breen, Gavan. 1988. Bularnu grammar and vocabulary machine-readable files. Australian Institute of Aboriginal and Torres Strait Islander Studies, Australian Indigenous Languages Collection. ASEDA 0007. Canberra. http://aiatsis.gov.au/sites/default/files/catalogue_resources/0007_access.zip

Bunuba

Centre, Kimberley Language Resource. 2010. *Bunuba draft dictionary.* Halls Creek, WA, Australia: Kimberley Language Resource Centre. https://www.klrc.org.au/dictionary/bunuba/lexicon/01.htm (26 July, 2018)

Burarra

CHIRILA source: CHIRILA/v1/Glasgow

Glasgow, Kathleen. 1994. *Burarra-Gun-nartpa dictionary with English finder list.* SIL

Butchulla

Bell, Jeanie. 2003. *A sketch grammar of the Badjala language of Gari (Fraser Island).* Melbourne: University of Melbourne M.A. Thesis

Central Arrernte

Wilkins, David. N.d. Mparntwe Arrernte (Aranda): Studies in the structure and semantics of grammar. Australian Institute of Aboriginal and Torres Strait Islander Studies, Australian Indigenous Languages Collection. ASEDA 0476. Canberra

Phonemic normalization: Labialized consonants normalized to C + w. Prestopped nasals normalized to stop + nasal sequence. Prepalatalized consonants normalized to j + C.

Dalabon

Evans, Nicholas, Francesca Merlan & Maggie Tukumba. 2004. *A first dictionary of Dalabon (Ngalkbon).* Maningrida, NT, Australia: Maningrida Arts & Culture. 489 pp.

Dhangu

Zorc, R. David. 2004. Yolngu Matha dictionary. Australian Institute of Aboriginal and Torres Strait Islander Studies, Australian Indigenous Languages Collection. ASEDA 0778. Canberra. http://aiatsis.gov.au/sites/default/files/catalogue_resources/0778_Access.zip

Phonemic normalization: Lenis retroflex stop normalized to retroflex flap.

Dharumbal

CHIRILA source: CHIRILA/v2/ter02

Terrill, Angela. 2002. *Dharumbal: The language of Rockhampton, Australia* (Pacific Linguistics 525). Canberra: Pacific Linguistics. 108 pp. https://doi.org/10.15144/PL-525

Dhay'yi

Wunungmurra, Djarrayang. 1993. Dhalwangu dictionary. Australian Institute of Aboriginal and Torres Strait Islander Studies, Australian Indigenous Languages Collection. ASEDA 0502. Canberra

Phonemic normalization: Lenis retroflex stop normalized to retroflex flap; all other voicing is allophonic.

Diyari

Austin, Peter K. 1981. *A grammar of Diyari, South Australia* (Cambridge Studies in Linguistics 32). Cambridge; New York: Cambridge University Press. 269 pp.

Phonemic normalization: Phonetic trill-released stop normalized as stop + trill. Otherwise, voiced stops normalized as taps.

Djabugay

Robertson, Sue & Bruce A. Sommer. 1997. Jaabugay dictionary. Australian Institute of Aboriginal and Torres Strait Islander Studies, Australian Indigenous Languages Collection. ASEDA 0013. Canberra

Djapu

CHIRILA source: CHIRILA/v1/mor83

Morphy, Frances. 1983. Djapu, a Yolngu dialect. In R. M. W. Dixon & Barry Blake (eds.), *Handbook of Australian languages*, vol. 3, 5 vols., 1–188. Amsterdam: John Benjamins

Phonemic normalization: Lenis retroflex stop normalized to retroflex flap.

Djinang

CHIRILA source: CHIRILA/v1/ASEDA0009

Waters, Bruce E. 1988. Djinang dictionary. Australian Institute of Aboriginal and Torres Strait Islander Studies, Australian Indigenous Languages Collection. ASEDA 0009. Canberra

Phonemic normalization: Glottal closure normalized to a segment phoneme.

Duungidjawu

CHIRILA source: CHIRILA/v2/K&W 04

Kite, Suzanne & Stephen A. Wurm. 2004. *The Duungidjawu language of southeast Queensland: Grammar, texts and vocabulary* (Pacific Linguistics 553). Canberra: Pacific Linguistics. 298 pp. https://doi.org/10.15144/PL-553

Dyirbal

CHIRILA source: CHIRILA/v1/dix72

Dixon, R. M. W. 1972. *The Dyirbal language of North Queensland*. Cambridge: Cambridge University Press

Emmi

Ford, Lysbeth J. 1998. *A description of the Emmi language of the Northern Territory of Australia*. Canberra: The Australian National University dissertation. 446 pp. http://hdl.handle.net/1885/10796 (12 July, 2018)

Erre

Birch, Bruce. 2006. *A first dictionary of Erre, Mengerrdji and Urningangk: Three languages from the Alligator Rivers region of the north western Arnhem Land, Northern Territory, Australia*. Jabiru, NT, Australia: Gundjeihmi Aboriginal Corporation. 125 pp.

Phonemic normalization: Double stops normalized as fortis.

Gamilaraay

CHIRILA source: CHIRILA/v1/ash03

Ash, Anna, John Giacon & Amanda Lissarrague. 2003. *Gamilaraay, Yuwaalaraay & Yuwaalayaay dictionary*. Alice Springs, NT, Australia: IAD Press. 344 pp.

Gangulu

CHIRILA source: CHIRILA/v1/Terrell

Terrill, Angela. 1999. Biri lexicons. Australian Institute of Aboriginal and Torres Strait Islander Studies, Australian Indigenous Languages Collection. ASEDA 0700. Canberra. http://aiatsis.gov.au/sites/default/files/catalogue_resources/0700_access.zip

Gidabal

CHIRILA source: CHIRILA/v1/cro78

Crowley, Terry. 1978. *The Middle Clarence dialects of Bandjalang.* Vol. 12 (Research and regional studies). Canberra: Australian Institute of Aboriginal Studies

Gooniyandi

Centre, Kimberley Language Resource. 1993. *Gooniyandi wordbook.* Halls Creek, Western Australia: Kimberley Language Resource Centre. 84 pp.

Gugu Badhun

CHIRILA source: CHIRILA/v1/sut73

Sutton, Peter John. 1973. Gugu-Badhun and its neighbours. In *Gugu-Badhun and its neighbours: A linguistic salvage study*, 24–67. Sydney: Macquarie University

Gumbaynggir

Murrbay Aboriginal and Culture Cooperative. 2001. *A Gumbaynggir language dictionary = Gumbayngirr bijarr jandaygam.* Canberra: Aboriginal Studies Press. 160 pp.

Gunya

CHIRILA source: CHIRILA/v1/dixbla81

Breen, Gavan. 1981a. Margany and Gunya. In R. M. W. Dixon & Barry Blake (eds.), *Handbook of Australian languages*, vol. 2, 275–394. Amsterdam: John Benjamins

Gupapuyngu

CHIRILA source: CHIRILA/v1/BL

Lowe, Beulah & Beulah Lowe. 1976. Temporary Gupapuyngu dictionary. Milingimbi, NT, Australia

Gurindji

Meakins, Felicity, Patrick McConvell, et al. 2013. *Gurindji to English dictionary.* Batchelor, NT, Australia: Batchelor Press. 596 pp.

Gurr-Goni

Green, Rebecca & Leila Nimbadja (eds.). 2015. *Gurr-Goni to English dictionary.* Google-Books-ID: gIGpDAEACAAJ. Batchelor, NT, Australia: Batchelor Press. 357 pp.

Phonemic normalization: Geminate stops normalized to fortis.

Guugu Yimidhirr

Haviland, John B. 1979. Guugu Yimidhirr. In R. M. W. Dixon & Barry Blake (eds.), *Handbook of Australian languages*, vol. 1, 5 vols., 26–180. Amsterdam: John Benjamins

Guwamu

CHIRILA source: CHIRILA/v1/Austin 1980

Austin, Peter K. 1980. Guwamu vocabulary and English-Guwamu finder list. Cambridge, MA

Iwaidja

Pym, Noreen & Bonnie Larrimore. 2011. *Iwaidja-English Interactive Dictionary.* AuSIL Interactive Dictionary Series A-2. In collab. with Charles E. Grimes & Maarten Lecompte. http://ausil.org/Dictionary/Iwaidja/lexicon/mainintro.htm (23 July, 2018)

Jaru

Tsunoda, Tasaku. 1981. Jaru wordlist. In David Nash (ed.). In collab. with Kathleen Menning, Joyce Hudson & G Cooling, *Sourcebook for Central Australian languages.* ASEDA 0119. Alice Springs, NT, Australia: Institute for Aboriginal Development

Phonemic normalization: iji and uwu normalized as long high vowels.

Jawoyn

Merlan, Francesca & Pascale Jacq. 2005. *Jawoyn-English dictionary and English finder-list.* In collab. with Jawoyn elders. Katherine, NT, Australia: Diwurruwurru-jaru Aboriginal Corporation. 342 pp.

Jiwarli

CHIRILA source: CHIRILA/v2/ASEDA0435

Austin, Peter K. N.d.(a). A dictionary of Jiwarli. Australian Institute of Aboriginal and Torres Strait Islander Studies, Australian Indigenous Languages Collection. ASEDA 0435. Canberra

Kalkatungu

CHIRILA source: CHIRILA/v2/ASEDA0205

Blake, Barry J. 1990a. Kalkatungu vocabulary. Australian Institute of Aboriginal and Torres Strait Islander Studies, Australian Indigenous Languages Collection. ASEDA 0205. Canberra

Phonemic normalization: Double short vowels normalized as long.

Karajarri

McKelson, Kevin R. 1989. Studies in Karajarri

Kariyarra

Smythe, Sue & Manny Lockyer. N.d. Kariyarra wordlist. Australian Institute of Aboriginal and Torres Strait Islander Studies, Australian Indigenous Languages Collection. ASEDA 0582. Canberra. http://aiatsis.gov.au/sites/default/files/catalogue_resources/0582_access.zip

Kartujarra

O'Grady, Geoffrey N. 1988a. Gardudjarra wordlist. Australian Institute of Aboriginal and Torres Strait Islander Studies, Australian Indigenous Languages Collection. ASEDA 0067. Canberra. http://aiatsis.gov.au/sites/default/files/catalogue_resources/0067_access.zip

Kija

CHIRILA source: CHIRILA/v1/bly01

Blyth, Noel. 2001. Wangka dictionary and grammar. Australian Institute of Aboriginal and Torres Strait Islander Studies, Australian Indigenous Languages Collection. ASEDA 0709. Canberra. http://aiatsis.gov.au/sites/default/files/catalogue_resources/0709_access.zip

Kok Nar

Sommer, Bruce A. N.d.(b). Koko Narr. Fryer Library Bruce Sommer Collection. UQFL476_b10f03_64, UQFL476_b10f03_65. Brisbane

Koko Bera

Black, Paul D. & Kokoberrin Tribal Aboriginal Corporation. 2007. The Kokoberrin and their languages. Australian Institute of Aboriginal and Torres Strait Islander Studies, Australian Indigenous Languages Collection. MS 4584

Kugu Nganhcara

CHIRILA source: CHIRILA/v1/ASEDA0021

Smith, Ian & Steve Johnson. 1989. Kugu Nganchara. Australian Institute of Aboriginal and Torres Strait Islander Studies, Australian Indigenous Languages Collection. ASEDA 0021. Canberra

Kukatj

Breen, Gavan. 1991. Kukatj grammar machine-readable files. Australian Institute of Aboriginal and Torres Strait Islander Studies, Australian Indigenous Languages Collection. ASEDA 0022. Canberra. http://aiatsis.gov.au/sites/default/files/catalogue_resources/0022_access.zip

Phonemic normalization: Featureless vowel normalized as schwa.

Kukatja

CHIRILA source: CHIRILA/v1/ASEDA0504

Peile, Anthony Rex & Hilaire Valiquette. N.d. A basic Kukatja to English dictionary. Australian Institute of Aboriginal and Torres Strait Islander Studies, Australian Indigenous Languages Collection. ASEDA 0504. Canberra

Kuku Yalanji

Hershberger, Henry D. & Ruth Hershberger. 1986. *Kuku-Yalanji dictionary.* In collab. with Australian Aborigines Branch Summer Institute of Linguistics. Vol. 7 (Work Papers of SIL - AAIB. Series B). Darwin: Summer Institute of Linguistics, Australian Aborigines Branch. 294 pp.

Kurrama

Dench, Alan C. N.d. Kurrama. Australian Institute of Aboriginal and Torres Strait Islander Studies, Australian Indigenous Languages Collection. ASEDA 0481. Canberra. http://aiatsis.gov.au/sites/default/files/catalogue_resources/0481_access.zip

Kurtjar

CHIRILA source: CHIRILA/v1/ASEDA0026

Black, Paul D. & Rolly Gilbert. 1988. Kurtjar dictionary. Australian Institute of Aboriginal and Torres Strait Islander Studies, Australian Indigenous Languages Collection. ASEDA 0026. Canberra

Phonemic normalization: Retroflex glide~tap normalized as glide.

Kuugu Ya'u

Thompson, David A. 1988. "Sand Beach" language: An outline of Kuuku Ya'u and Umpila. Australian Institute of Aboriginal and Torres Strait Islander Studies, Australian Indigenous Languages Collection. ASEDA 0027. Canberra

Lardil

Hale, Kenneth & Ngakulmungan Kangka Leman. 1997. *Lardil dictionary: A vocabulary of the language of the Lardil people, Mornington Island, Gulf of Carpentaria, Queensland; with English-Lardil finder list.* Gununa, QLD, Australia: Mornington Shire Council. 347 pp.

Larrakia

Harvey, Mark. 2004. *Larrakia dictionary.* Darwin: ATSIC, Yirra Bandoo Aboriginal Corporation. 92 pp.

Limilngan

Harvey, Mark. 2001. *A grammar of Limilngan: A language of the Mary River region, Northern Territory, Australia* (Pacific Linguistics 516). Canberra: Pacific Linguistics. 209 pp. https://doi.org/10.15144/PL-516

Linngithigh

Hale, Kenneth. 1999. A Linngithigh vocabulary. Australian Institute of Aboriginal and Torres Strait Islander Studies, Australian Indigenous Languages Collection. ASEDA 0687. Canberra

Phonemic normalization: Trill-released stop normalized as stop + trill. Prenasalized stops normalized to nasal + lenis stop.

Malkana

Gargett, Andrew. 2011. *A salvage grammar of Malgana, the language of Shark Bay, Western Australia* (Pacific Linguistics 624). Canberra: Pacific Linguistics. 102 pp. https://doi.org/10.15144/PL-624

Malyangapa

Hercus, Luise A. 1989. Maljangapa-Wadigali vocabulary. Australian Institute of Aboriginal and Torres Strait Islander Studies, Australian Indigenous Languages Collection. ASEDA 0246. Canberra. http://aiatsis.gov.au/sites/default/files/catalogue_resources/0246_access.zip

Mangala

McKelson, Kevin. 1989a. Mangala wordlist. Australian Institute of Aboriginal and Torres Strait Islander Studies, Australian Indigenous Languages Collection. ASEDA 0220. Canberra. http://aiatsis.gov.au/sites/default/files/catalogue_resources/0220_access.zip

Margany

CHIRILA source: CHIRILA/v1/bre81

Breen, Gavan. 1981a. Margany and Gunya. In R. M. W. Dixon & Barry Blake (eds.), *Handbook of Australian languages*, vol. 2, 275–394. Amsterdam: John Benjamins

Marra

Heath, Jeffrey. 1981. *Basic materials in Mara: Grammar, texts and dictionary* (Pacific Linguistics Series C 60). Canberra: Pacific Linguistics. 534 pp. https://doi.org/10.15144/PL-C60

Martuthunira

Dench, Alan C. 1995. *Martuthunira, a language of the Pilbara region of Western Australia* (Pacific Linguistics Series C 125). Canberra: Pacific Linguistics. 406 pp. https://doi.org/10.15144/PL-C125

Matngele

Zandvoort, Franklin D. 1999. *A grammar of Matngele.* Armidale, NSW, Australia: University of New England B.A. (Hons)

Mawng

Singer, Ruth et al. 2015. *Mawng dictionary v1.0.* mawngngaralk.org.au/main/dictionary.php (23 July, 2018)

Mbabaram

Dixon, R. M. W. 1991a. Mbabaram. In R. M. W. Dixon & Barry J. Blake (eds.), *Handbook of Australian languages*, vol. 4, 5 vols., 348–402. Melbourne: Oxford University Press

Phonemic normalization: Labialized consonants normalized to C + w. Final schwa treated as a phoneme.

Mengerrdji

Birch, Bruce. 2006. *A first dictionary of Erre, Mengerrdji and Urningangk: Three languages from the Alligator Rivers region of the north western Arnhem Land, Northern Territory, Australia.* Jabiru, NT, Australia: Gundjeihmi Aboriginal Corporation. 125 pp.

Phonemic normalization: Double stops normalized as fortis.

Miriwoong

Kofod, F. M. 1976. Miriwung - English. Australian Institute of Aboriginal and Torres Strait Islander Studies, Australian Indigenous Languages Collection. MS 1896. Canberra

Mirniny

O'Grady, Geoffrey N. & Edward M. Curr. 1988. Mirniny wordlist. Australian Institute of Aboriginal and Torres Strait Islander Studies, Australian Indigenous Languages Collection. ASEDA 0070. Canberra. http://aiatsis.gov.au/sites/default/files/catalogue_resources/0070_access.zip

Mudburra

Nash, David et al. 1988. Mudburra wordlist. Australian Institute of Aboriginal and Torres Strait Islander Studies, Australian Indigenous Languages Collection. ASEDA 0031. Canberra. http://aiatsis.gov.au/sites/default/files/catalogue_resources/0031_access.zip

Murrinh-patha

Street, Chester S. 1987. *An introduction to the language and culture of the Murrinh-Patha.* Darwin: Summer Institute of Linguistics. Australian Aborigines Branch. 117, plus audio cassette

Muruwari

CHIRILA source: CHIRILA/v1/ASEDA0252

Oates, Lynette Frances. 1992. *Muruwari (Moo-roo-warri) dictionary: Words of an Aboriginal language of north-western New South Wales.* Albury, NSW, Australia: Graeme van Brummelen, produced with the assistance of the Australian Institute of Aboriginal & Torres Strait Islander Studies. 97 pp.

Nakara

Eather, Bronwyn, Yurrbukka Community & Bawinanga Aboriginal Corporation. 2005. *A first dictionary of Na-Kara.* In collab. with Jimmy Kalamirnda. Winnellie, NT, Australia: Maningrida Arts & Culture. 199 pp.

Phonemic normalization: Geminate stops normalized to fortis.

Ngaanyatjarra

Glass, Amee. 1988. Ngaanyatjarra wordlist. Australian Institute of Aboriginal and Torres Strait Islander Studies, Australian Indigenous Languages Collection. ASEDA 0033. Canberra. http://aiatsis.gov.au/sites/default/files/catalogue_resources/0033_access.zip

Ngadjunmaya

Wangka Maya Pilbara Aboriginal Language Centre. 2008a. *Ngajumaya dictionary 2008.* South Hedland, WA, Australia: Wangka Maya Pilbara Aboriginal Language Centre. 16 pp.

Ngalakgan

CHIRILA source: CHIRILA/v1/mor83

Merlan, Francesca C. 1983. *Ngalakan grammar, texts and vocabulary* (Pacific Linguistics Series B 89). Canberra: Pacific Linguistics. 229 pp. https://doi.org/10.15144/PL-B89

Phonemic normalization: Geminate stops normalized to fortis.

Ngandi

Heath, Jeffrey. 1978. *Ngandi grammar, texts and dictionary.* Canberra: Australian Institute of Aboriginal Studies

Ngardily

Green, Thomas M. 1988. Ngardily wordlist. Australian Institute of Aboriginal and Torres Strait Islander Studies, Australian Indigenous Languages Collection. ASEDA 0034. Canberra. http://aiatsis.gov.au/sites/default/files/catalogue_resources/0034_access.zip

Ngarinyin

Coate, Howard H. J. & A. P. Elkin. 1974. *Ngarinjin-English dictionary* (Oceania linguistic monographs 16). Sydney: University of Sydney. 534 pp.

Ngarinyman

Jones, Caroline. 2005. Ngarinman vocabulary. Australian Institute of Aboriginal and Torres Strait Islander Studies, Australian Indigenous Languages Collection. ASEDA 0796. Canberra

Ngarla

Brown, Alexander & Brian Geytenbeek. N.d. Ngarla-English dictionary (interim), English-Ngarla wordlist. Australian Institute of Aboriginal and Torres Strait Islander Studies, Australian Indigenous Languages Collection. ASEDA 0060. Canberra

Ngarluma

Hale, Kenneth. 1989. Ngarluma wordlist. Australian Institute of Aboriginal and Torres Strait Islander Studies, Australian Indigenous Languages Collection. ASEDA 0037. Canberra. http://aiatsis.gov.au/sites/default/files/catalogue_resources/0037_access.zip

Ngawun

CHIRILA source: CHIRILA/v2/BreenMayi

Breen, Gavan. 1981b. *The Mayi languages of the Queensland Gulf Country* (A.I.A.S. New Series 29). Canberra: Australian Institute of Aboriginal Studies. 238 pp.

Ngiyambaa

CHIRILA source: CHIRILA/v1/don-lex

Donaldson, Tamsin. 1997. *Ngiyambaa wordworld.* Canberra: The Author, Australian Institute of Aboriginal & Torres Strait Islander Studies

Nhanda

CHIRILA source: CHIRILA/v1/ble01

Blevins, Juliette. 2001. *Nhanda: An Aboriginal language of Western Australia* (Oceanic linguistics special publication 30). Honolulu: University of Hawai'i Press. 170 pp.

Nhangu

CHIRILA source: CHIRILA/v1/CB-fieldnotes

James, Bentley. 2003. *Yan-nhangu dictionary.* In collab. with Laurie Baymarrwanga et al. Milingimbi, NT, Australia: B. James. 34 pp.

Nhirrpi

CHIRILA source: CHIRILA/v1/bow-nhi

Bowern, Claire. 1999. Nhirrpi vocabulary, based on fieldnotes of S. A. Wurm

Nukunu

CHIRILA source: CHIRILA/v2/her92

Hercus, Luise A. 1992a. *A Nukunu dictionary.* Canberra: Department of Linguistics, Australian National University. 51 pp.

Phonemic normalization: Voiced retroflex stop normalized to retroflex tap.

Nungali

Bolt, Janet E., W. G. Hoddinott & F. M. Kofod. 1971. *An elementary grammar of the Ngaliwuru language of the Northern Territory.* MS 211, mimeographed. Canberra: Australian Institute of Aboriginal & Torres Strait Islander Studies, Australian Indigenous Languages Collection

Nyamal

Burgman, Albert. 2007b. *Nyamal dictionary: English-Nyamal finderlist and topical wordlist.* In collab. with Wangka Maya Pilbara Aboriginal Language Centre. South Hedland, WA, Australia: Wangka Maya Pilbara Aboriginal Language Centre. 59 pp.

Nyangumarta

Geytenbeek, Brian, Helen Geytenbeek & Wangka Maya Pilbara Aboriginal Language Centre. 1991. *Nyangumarta-English dictionary (interim), with an English-Nyangumarta finder list.* Port Hedland, WA, Australia: Wangka Maya Pilbara Aboriginal Language Centre. 119 pp.

Nyawaygi

Dixon, R. M. W. 1983. Nyawaygi. In R. M. W. Dixon & Barry J. Blake (eds.), *Handbook of australian languages*, vol. 3, 5 vols., 431–531. Amsterdam: John Benjamins

Nyikina

CHIRILA source: CHIRILA/v1/Stokes

Stokes, Bronwyn. N.d. Nyikina-English: A first lexicon. Australian Institute of Aboriginal and Torres Strait Islander Studies, Australian Indigenous Languages Collection. ASEDA0472. Canberra. http://aiatsis.gov.au/sites/default/files/catalogue_resources/0472_access.zip

Nyiyaparli

O'Grady, Geoffrey N. 1988b. Nyiyabali wordlist. Australian Institute of Aboriginal and Torres Strait Islander Studies, Australian Indigenous Languages Collection. ASEDA 0074. Canberra. http://aiatsis.gov.au/sites/default/files/catalogue_resources/0074_access.zip

Ogh Angkula

Sommer, Bruce A. N.d.(a). ANkula. Fryer Library Bruce Sommer Collection. UQFL476_b10f04. Brisbane

Ogh Unyjan

Sommer, Bruce A. N.d.(c). Ogh Unydjan. Fryer Library Bruce Sommer Collection. UQFL476_b09f03_s05. Brisbane

Olkol

Hamilton, Philip. 1997b. *Uw Olkola and Uw Oykangand Alphabetical Search Index.* Oykangand and Olkola Multimedia Dictionary. http://www.oocities.org/athens/delphi/2970/olkola.htm

Oykangand

Hamilton, Philip. 1997b. *Uw Olkola and Uw Oykangand Alphabetical Search Index.* Oykangand and Olkola Multimedia Dictionary. http://www.oocities.org/athens/delphi/2970/olkola.htm

Panyjima

Dench, Alan C. 1991. Panyjima. Australian Institute of Aboriginal and Torres Strait Islander Studies, Australian Indigenous Languages Collection. ASEDA 0375. Canberra. http://aiatsis.gov.au/sites/default/files/catalogue_resources/0375_access.zip

Patjtjamalh

Ford, Lysbeth J. 1997. *Batjamalh: Dictionary and texts.* Bungendore, NSW, Australia: L.J. Ford. 108 pp.

Payungu

CHIRILA source: CHIRILA/v1/ASEDA0394

Austin, Peter K. N.d.(d). Payungu - English dictionary. Australian Institute of Aboriginal and Torres Strait Islander Studies, Australian Indigenous Languages Collection. ASEDA 0394. Canberra

Pintupi

Hansen, Kenneth & Lesley Hansen. 1992. *Pintupi/Luritja dictionary.* 3rd edn. Alice Springs, NT, Australia: Institute for Aboriginal Development. 267 pp.

Pitta Pitta

CHIRILA source: CHIRILA/v1/bla0275

Blake, Barry J. 1990b. Pitta Pitta wordlist. Australian Institute of Aboriginal and Torres Strait Islander Studies, Australian Indigenous Languages Collection. ASEDA 0275. Canberra

Purduna

Burgman, Albert. 2007a. *Burduna dictionary: English-Burduna wordlist and thematic wordlist.* In collab. with Wangka Maya Pilbara Aboriginal Language Centre. South Hedland, WA, Australia: Wangka Maya Pilbara Aboriginal Language Centre. 86 pp.

Putijarra

Wangka Maya Pilbara Aboriginal Language Centre & Australian Institute of Aboriginal and Torres Strait Islander Studies. 2004. *Putijarra-English wordlist, English-Putijarra finder topical wordlist &sketch morphology.* South Hedland, WA, Australia: Wangka Maya Pilbara Aboriginal Language Centre. 118 pp.

Rembarrnga

McKay, Graham. 2011. Rembarrnga dictionary and grammar. Australian Institute of Aboriginal and Torres Strait Islander Studies, Australian Indigenous Languages Collection. ASEDA 0607. Canberra. https://iats.ent.sirsidynix.net.au/client/en_AU/external/search/detailnonmodal/ent:$002f$002fSD_ILS$002f0$002fSD_ILS:402512/one?qu=AILEC+0607

Phonemic normalization: Geminate stops normalized to fortis.

Ritharrngu

CHIRILA source: CHIRILA/v1/Heath

Heath, Jeffrey. 1976b. Ritharngu. In Robert M. W. Dixon (ed.), *Grammatical categories in australian languages* (Linguistic series 22), 285–287. Canberra: Australian Institute of Aboriginal Studies

Southern Paakintyi

Hercus, Luise A. N.d.(a). Paakantyi dictionary. Australian Institute of Aboriginal and Torres Strait Islander Studies, Australian Indigenous Languages Collection. ASEDA 0525. Canberra. http://aiatsis.gov.au/sites/default/files/catalogue_resources/0525_access.zip

Thaayorre

Foote, Tom & Allen Hall. 1993. *Kuuk Thaayorre dictionary: Thaayorre/English; September, 1966-92.* Brisbane: Jolien Press. 239 pp.

Thalanyji

CHIRILA source: CHIRILA/v2/ASEDA0437

Austin, Peter K. N.d.(b). A dictionary of Thalanyji. Australian Institute of Aboriginal and Torres Strait Islander Studies, Australian Indigenous Languages Collection. ASEDA 0437. Canberra

Tharrkari

Austin, Peter K. 1992. *A dictionary of Tharrgari, Western Australia.* Bundoora, Victoria, Australia: La Trobe University. 60 pp.

Thaynakwithi

Fletcher, Gloria Thancoupie. 2007. *Thanakupi's guide to language and culture: A Thaynakwith dictionary.* North Sydney, NSW, Australia: Jennifer Isaacs Arts & Publishing. 144 pp.

Thirarri

Austin, Peter K. 1981. *A grammar of Diyari, South Australia* (Cambridge Studies in Linguistics 32). Cambridge; New York: Cambridge University Press. 269 pp.

Phonemic normalization: Phonetic trill-released stop normalized as stop + trill. Otherwise, voiced stops normalized as taps.

Tiwi

Lee, Jenny. 2013. *Tiwi-English Interactive Dictionary.* AuSIL Interactive Dictionary Series A-4. In collab. with Charles E. Grimes & Maarten Lecompte. http://ausil.org/Dictionary/Tiwi/lexicon/main.htm (23 July, 2018)

Phonemic normalization: r + alveolar normalized as retroflex. Following Breen 1979, (w)o is normalized as wa.

Umpila

O'Grady, Geoffrey N. 1988c. Umpila wordlist. Australian Institute of Aboriginal and Torres Strait Islander Studies, Australian Indigenous Languages Collection. ASEDA 0094. Canberra. http://aiatsis.gov.au/sites/default/files/catalogue_resources/0094_access.zip

Unggumi

McGregor, William. 1985. *Handbook of Kimberley languages: Word lists.* Vol. 2. Broome: Kimberley Resource Centre

Urningangg

Birch, Bruce. 2006. *A first dictionary of Erre, Mengerrdji and Urningangk: Three languages from the Alligator Rivers region of the north western Arnhem Land, Northern Territory, Australia.* Jabiru, NT, Australia: Gundjeihmi Aboriginal Corporation. 125 pp.

Waalubal

CHIRILA source: CHIRILA/v1/cro78

Crowley, Terry. 1978. *The Middle Clarence dialects of Bandjalang.* Vol. 12 (Research and regional studies). Canberra: Australian Institute of Aboriginal Studies

Waanyi

CHIRILA source: CHIRILA/v1/WaanyiDict

Laughren, Mary. 2016. Waanyi dictionary database. Brisbane

Wagiman

Wilson, Stephen & Mark Harvey. 2001. *The Wagiman online dictionary.* The University of Syndey. In collab. with Lulu Martin Dalpbalngali. http://sydney.edu.au/arts/linguistics/research/wagiman/dict/dict.html (9 July, 2018)

Walmajarri

Hudson, Joyce & Eirlys Richards. 1993. Walmajarri dictionary. Australian Institute of Aboriginal and Torres Strait Islander Studies, Australian Indigenous Languages Collection. ASEDA 0167. Canberra

Wambaya

CHIRILA source: CHIRILA/v1/RN Wambaya 29,269,292; NE NA

Wangkatja

Blyth, Noel. 2001. Wangka dictionary and grammar. Australian Institute of Aboriginal and Torres Strait Islander Studies, Australian Indigenous Languages Collection. ASEDA 0709. Canberra. http://aiatsis.gov.au/sites/default/files/catalogue_resources/0709_access.zip

Wangkumara

CHIRILA source: CHIRILA/v1/robnd

Robertson, Carol. 1985. *Wangkumara grammar and dictionary.* Sydney: Department of Technical & Further Education, Aboriginal Education Unit. 90 pp.

Phonemic normalization: Double a normalized to long vowel.

Wanyjirra

Senge, Chikako. 2015. *A grammar of Wanyjirra, a language of northern Australia.* Canberra: The Australian National University dissertation. https://openresearch-repository.anu.edu.au/bitstream/1885/109341/3/Senge%20Thesis%202016.pdf (23 July, 2018)

Wardaman

CHIRILA source: CHIRILA/v1/Merlan NA

Warlmanpa

Nash, David, Kenneth Hale & Gavan Breen. 1984. Preliminary vocabulary of Warlmanpa. Australian Institute of Aboriginal and Torres Strait Islander Studies, Australian Indigenous Languages Collection. ASEDA 0049. Canberra

Warlpiri

CHIRILA source: CHIRILA/v2/WarlpiriDict

Swartz, Steve. 1996. Warlpiri draft dictionary

Warluwarra

Breen, Gavan. 1990. Warluwara grammar and wordlist. Australian Institute of Aboriginal and Torres Strait Islander Studies, Australian Indigenous Languages Collection. ASEDA 0253. Canberra

Phonemic normalization: Prenasalized stops normalized to nasal + lenis stop. Tense glides normalized to fricatives. Tense lateral normalized to double lateral.

Warndarrang

CHIRILA source: CHIRILA/v1/hea80

Heath, Jeffrey. 1980. *Basic materials in Warndarang: Grammar, texts and dictionary* (Pacific Linguistics Series B 72). Canberra: Pacific Linguistics. 186 pp. https://doi.org/10.15144/PL-B72

Warnman

CHIRILA source: CHIRILA/v2/ASEDA0334

Wangka Maya Pilbara Aboriginal Language Centre. N.d. Warnman wordlist. Australian Institute of Aboriginal and Torres Strait Islander Studies, Australian Indigenous Languages Collection. ASEDA 0334. Canberra. http://aiatsis.gov.au/sites/default/files/catalogue_resources/0334_Access.zip

Warrgamay

CHIRILA source: CHIRILA/v1/dixbla81

Dixon, R. M. W. 1981. Wargamay. In R. M. W. Dixon & Barry Blake (eds.), *Handbook of Australian languages*, vol. 2, 5 vols., 1–145. Amsterdam: John Benjamins

Warriyangga

Austin, Peter K. N.d.(c). A dictionary of Warriyangka. Australian Institute of Aboriginal and Torres Strait Islander Studies, Australian Indigenous Languages Collection. ASEDA 0439. Canberra

Watjarri

Mackman, Doreen (ed.). 2012. *Wajarri dictionary: The language of the Murchison Region of Western Australia.* In collab. with Irra Wangga Language Centre & Yamaji Language Aboriginal Corporation. Geraldton, WA, Australia: Irra Wangga Language Centre. 249 pp. http://www.bundiyarra.com.au/wajarriApp/ (23 July, 2018)

Wemba Wemba

Hercus, Luise A. 1992b. *Wembawemba dictionary.* Canberra: L.A. Hercus. 116 pp.

Western Arrernte

Breen, Gavan. 2000. *Introductory dictionary of Western Arrernte.* In collab. with John Pfitzner. Alice Springs, NT, Australia: IAD Press. 120 pp.

Phonemic normalization: Labialized consonants normalized to C + w. Prestopped nasals normalized to stop + nasal sequence. Prepalatalized consonants normalized to j + C.

Western Wakaya

Breen, Gavan. 2006. Wakaya. Australian Institute of Aboriginal and Torres Strait Islander Studies, Australian Indigenous Languages Collection. ASEDA 0047. Canberra. http://aiatsis.gov.au/sites/default/files/catalogue_resources/0047_access.zip (30 July, 2018)

Wik Mungkan

Kilham, Christine et al. 2011. *Wik Mungkan-English Interactive Dictionary.* AuSIL Interactive Dictionary Series A-6. In collab. with Charles E. Grimes & Maarten Lecompte. http://ausil.org/Dictionary/Wik-Mungkan/lexicon/mainintro.htm (26 July, 2018)

Wik-Ngathan

CHIRILA source: CHIRILA/v2/PS

Sutton, Peter. 1995. *Wik-Ngathan dictionary.* Prospect, South Australia: Caitlin Press. 182 pp.

Wirangu

Hercus, Luise A. 1999. *A grammar of the Wirangu language from the West Coast of South Australia* (Pacific Linguistics Series C 150). Canberra: Pacific Linguistics. 239 pp. https://doi.org/10.15144/PL-C150

Phonemic normalization: Double a normalized to long vowel.

Wiri

Terrill, Angela. 1999. Biri lexicons. Australian Institute of Aboriginal and Torres Strait Islander Studies, Australian Indigenous Languages Collection. ASEDA 0700. Canberra. http://aiatsis.gov.au/sites/default/files/catalogue_resources/0700_access.zip

Worrorra

McGregor, William. 1985. *Handbook of Kimberley languages: Word lists.* Vol. 2. Broome: Kimberley Resource Centre

Wotjobaluk

Hercus, Luise A. N.d.(b). Wergaia vocabulary 1. Australian Institute of Aboriginal and Torres Strait Islander Studies, Australian Indigenous Languages Collection. ASEDA 0273. Canberra. http://aiatsis.gov.au/sites/default/files/catalogue_resources/0273_access.zip

Wubuy

CHIRILA source: CHIRILA/v1/Nunggubuyu Flora Fauna

Heath, Jeffrey. 1976a. Nunggubuyu flora and fauna terminology. Australian Institute of Aboriginal and Torres Strait Islander Studies, Australian Indigenous Languages Collection. Canberra

Phonemic normalization: Morphophonemic w1, w2 represented as their phonemic realizations w,b,k.

Yadhaykenu

Crowley, Terry. 1983. Uradhi. In R. M. W. Dixon & Barry J. Blake (eds.), *Handbook of Australian languages*, vol. 3, 5 vols., 307–428. Amsterdam: John Benjamins

Yalarnnga

CHIRILA source: CHIRILA/v1/ASEDA0204

Breen, Gavan & Barry J Blake. N.d. Yalarnnga vocab. Australian Institute of Aboriginal and Torres Strait Islander Studies, Australian Indigenous Languages Collection. ASEDA 0204. Canberra

Yandruwandha

CHIRILA source: CHIRILA/v1/breyandr

Breen, Gavan. 2004. *Innamincka talk: A grammar of the Innamincka dialect of Yandruwandha with notes on other dialects* (Pacific Linguistics 558). Canberra: Pacific Linguistics. 245 pp. https://doi.org/10.15144/PL-558

Phonemic normalization: Trill-released stop normalized as stop + trill. Prestopped laterals normalized to stop + lateral sequence.

Yanyuwa

Bradley, John. N.d. Yanyuwa dictionary. Australian Institute of Aboriginal and Torres Strait Islander Studies, Australian Indigenous Languages Collection. ASEDA 0382. Canberra

Phonemic normalization: Prenasalized stops normalized to nasal + stop sequence.

Yawijibaya

McGregor, William. 1985. *Handbook of Kimberley languages: Word lists.* Vol. 2. Broome: Kimberley Resource Centre

Yaygir

CHIRILA source: CHIRILA/v1/morelli2011

Morelli, Steve. 2012. *Yaygirr dictionary and grammar.* In collab. with Many Rivers Aboriginal Language Centre. Nambucca Heads, NSW, Australia: Muurrbay Aboriginal Language & Culture Co-operative. 254 pp.

Yidiny

CHIRILA source: CHIRILA/v2/dix91

Dixon, R. M. W. 1991b. *Words of our country: Stories, place names, and vocabulary in Yidiny, the Aboriginal language of the Cairns-Yarrabah region.* In collab. with Tony Irvine. St Lucia, Qld: University of Queensland Press. 312 pp.

Yindjibarndi

Anderson, Bruce, E. Richards & Summer Institute of Linguistics. N.d. Yindjibarndi dictionary. Australian Institute of Aboriginal and Torres Strait Islander Studies, Australian Indigenous Languages Collection. ASEDA 0297. Canberra. http://aiatsis.gov.au/sites/default/files/catalogue_resources/0297_access.zip

Yinhawangka

Wangka Maya Pilbara Aboriginal Language Centre. 2008b. *Yinhawangka dictionary: English-Yinhawangka wordlist and topical wordlists 2008: draft 1.* South Hedland, WA, Australia: Wangka Maya Pilbara Aboriginal Language Centre. 92 pp.

Yintyingka

Verstraete, Jean-Christophe & Bruce Rigsby. 2015. *A grammar and lexicon of Yintyingka* (Pacific Linguistics 648). Boston, Berlin: De Gruyter Mouton. 414 pp. https://doi.org/10.1515/9781614519003

Yir Yoront

Alpher, Barry. 1991. *Yir-Yoront lexicon: Sketch and dictionary of an Australian language.* Vol. 6 (Trends in Linguistics: Documentation). Berlin, New York: Mouton de Gruyter

Yorta Yorta

CHIRILA source: CHIRILA/v1/bowmor99

Bowe, Heather & Stephen Morey. 1999. *The Yorta Yorta (Bangerang) language of the Murray Goulburn including Yabula Yabula* (Pacific Linguistics Series C 154). Canberra: Pacific Linguistics. 286 pp.

Yulparija

McKelson, Kevin. 1989b. Yulparija. Australian Institute of Aboriginal and Torres Strait Islander Studies, Australian Indigenous Languages Collection. ASEDA 0032. Canberra. http://aiatsis.gov.au/sites/default/files/catalogue_resources/0032_access.zip

Yuwaalaraay

CHIRILA source: CHIRILA/v1/ash03

Ash, Anna, John Giacon & Amanda Lissarrague. 2003. *Gamilaraay, Yuwaalaraay & Yuwaalayaay dictionary.* Alice Springs, NT, Australia: IAD Press. 344 pp.

Yuwaliyaay

CHIRILA source: CHIRILA/v1/ash03

Ash, Anna, John Giacon & Amanda Lissarrague. 2003. *Gamilaraay, Yuwaalaraay & Yuwaalayaay dictionary.* Alice Springs, NT, Australia: IAD Press. 344 pp.

S4. Comparison of phonemes in the Ausphon lexical data and Phoible

A reviewer asked whether the AusPhon lexical datasets contain at least one token of each phoneme that we believe the language has, and suggested comparing the AusPhon datasets against the phoneme inventories in Phoible (https://phoible.org/) (Moran & McCloy 2019). Here we make that comparison, against the Australian languages inventories in the Phoible "ER" dataset (Round 2019).

Load Phoible ER and AusPhon data

```
library(tidyverse)

phoible_ER <-
  read_csv(url('https://github.com/phoible/dev/blob/master/data/phoible.csv?raw=true')) %>%
  filter(Source == "er") %>%
  select(language = LanguageName, phoneme = Phoneme)

data_file_newest <- rev(list.files("../data", "Aus_segment_frequencies"))[1]
ausphon <-
  read_tsv(file.path("../data", data_file_newest)) %>%
  select(language = variety_name, phoneme = match)

## Subset both datasets to just the overlapping languages
matching_languages <- intersect(phoible_ER$language, ausphon$language)

phoible_ER <- phoible_ER %>% filter(language %in% matching_languages)
ausphon <- ausphon %>% filter(language %in% matching_languages)
```

Patch Phoible ER

At time of writing, Phoible ER dataset contains some errors which will be corrected soon. This code provides a corresponding patch:

```
# Add some phonemes to Phoible ER
phoible_ER <-
  phoible_ER %>%
  bind_rows(
    data.frame(
      stringsAsFactors = FALSE,
      language =
        c("Thaynakwithi", "Tharrkari", "Nhirrpi", "Djapu",
          "Anguthimri", rep("Angkamuthi", 2),
          rep("Linngithigh", 5)),
      phoneme =
        c("\u0278", "l\u032a", "\u027e", "\u0294",
          "\u0294", "e", "e\u02d0",
          "ø", "\u00e3", "æ\u0303", "\u0129", "\u00f5"))
  )

# Remove some phonemes from Phoible ER
phoible_ER <-
  phoible_ER %>%
  filter(
    !(language == "Ngandi" & phoneme == "u\u02d0"),
    !(language == "Nungali" & phoneme == "r\u033a"),
    !(language == "Gidabal" & phoneme == "\u0279\u033a"),
```

```
    !(language == "Malkana" & phoneme == "i\u02d0"),
    !(language == "Tiwi" & phoneme == "l\u032a"),
    !(language == "Pitta Pitta" & phoneme == "o"),
    !(language == "Kartujarra" & phoneme == "\u0259"),
    !(language == "Gangulu" & phoneme == "\u0259"),
    !(language == "Mangala" & phoneme %in% c("i\u02d0", "o\u02d0", "o")),
    !(language == "Malyangapa" & phoneme == "\u0234"),
    !(language == "Thaynakwithi" & phoneme %in% c("i\u02d0", "o\u02d0", "u\u02d0"))
  )
```

Sources occasionally list a phoneme in an inventory but add text, to hedge that it might not exist:

- Limilngan: Harvey (2001: p.11) places "?" against the fortis retroflex stop phoneme, presumably because he had no confirmation of it. The wordlist contains none either.
- Bularnu: Breen (1978: p.3) writes "It is possible that vowel length is phonemic", i.e., it's not confirmed. The wordlist data confirms /a:/ but not /i:, u:/.
- Ngadjunmaya is a Mirniny language. Its Phoilbe source is O'Grady, Voegelin & Voegelin (1966), which notes (p134) that the distinctiveness of the laminals is uncertain. Phoibe includes a dental lateral but the Ausphon data doesn't. It's likely, then, that the dental lateral is not a distinctive phoneme.
- Kok Nar: Breen (1976: pp.244-5) details his uncertainty about the schwa phoneme, but lists it tentatively. The Ausphon data source (Sommer n.d.[b]) doesn't have clear evidence of a contrast.
- Atampaya and Yadhaykenu: Crowley (1983: p.318) states that /e:/ occurs in "no attested minimal pairs" and is "even more marginal" than "very low frequency" /e/ which raises reasonable questions as to its existence. His own wordlists do not contain it.
- Adnyamathanha: McEntee & McKenzie (1992: p.17), the dictionary use for AusPhon, note there is just one word with an intervocalic lenis velar obstruent, and this is the only item preventing the lenis and fortis series from being collapsed into a single phonemic category. Schebeck (1974), used for Phoible, does not include the contrast.
- Yorta Yorta: Bowe & Morey (1999: p.42) are unsure if the retroflex glide is distinctive.

We remove these suspect phonemes where they don't occur in the AusPhon data:

```
phoible_ER <-
  phoible_ER %>%
  filter(
    !(language == "Limilngan" & phoneme == "\u0288\u0348"),
    !(language == "Bularnu" & phoneme %in% c("i\u02d0", "u\u02d0")),
    !(language == "Ngadjunmaya" & phoneme == "l\u032a"),
    !(language == "Kok Nar" & phoneme == "\u0259"),
    !(language %in% c("Atampaya", "Yadhaykenu") & phoneme == c("e\u02d0"))
  )
```

Conversely, we add them where they do appear in AusPhon, but are not in Phoible ER:

```
phoible_ER <-
  phoible_ER %>%
  bind_rows(
    data.frame(
      stringsAsFactors = FALSE,
      language = c("Yorta Yorta", "Adnyamathanha"),
      phoneme = c("\u0279\u033a", "\u0263"))
  )
```

Some sources used for Phoible ER are ambiguous, or in one case may contain an error, regarding certain phonemes, as revealed by our comparison with AusPhon:

- Margany: Breen (1981a) describes both Margany & Gunya. He does not provide any examples of /i:/

in Margany, but also does not say iː is a gap. Thus, it seems that the presentation in Breen (1981a) should be read as ambiguous: /iː/ is part of Gunya but not known to be part of Margany.
- Malyangapa: O'Grady, Voegelin & Voegelin (1966) provide no more than a table of phonemes. It has /i a u/ "plus length"; but this appears to be incorrect for the hig vowels.
- Putijarra: Wangka Maya (2004), the source for both Phoible & AusPhon, lists a dental lateral but this may possibly be a copy-and-paste error from the front matter of another dictionary.

Since these don't show up in the AusPhon lexical data, we remove them also:

```
phoible_ER <-
  phoible_ER %>%
  filter(
    !(language == "Margany" & phoneme == "i\u02d0"),
    !(language == "Malyangapa" & phoneme %in% c("i\u02d0", "u\u02d0")),
    !(language == "Putijarra" & phoneme == "l\u032a")
  )
```

AusPhon normalizes the analysis of rounding, and removes foreign phonemes, in a way that also causes some divergence from the Phoible inventories. We'll bring these into line:

```
# Ausphon represents the "labialised" clusters in Arrandic languages as
# cluster + /w/ rather than with labialised consonants as in Phoilbe.
# Similarly, Tiwi /o/ is analysed as a sequence /w a/. Remove those phoible
# consonants:

phoible_ER <-
  phoible_ER %>%
  filter(!str_detect(phoneme, "\u02b7")) %>%
  filter(!(language == "Tiwi" & phoneme == "o"))

# Ausphon marks foreign segments (appearing only in words noted as borrowed,
# or obviously borrowed from English), and we excluded these from our study.
# Warndarrang's dental t is marked as foreign in this way.

phoible_ER <-
  phoible_ER %>%
  filter(!(language == "Warndarrang" & phoneme == "t\u032a"))
```

This completes our 'patch' on the Phoible ER dataset.

Transpose equivalent phonemes with differing symbolization

Many equivalent phonemes have different symbolizations in the two datasets. Here we build up a dataframe that records those equivalences.

```
# Define a helper function
add_to_df = function(df, lang, ph, au) {
  bind_rows(
    df,
    data.frame(
      language = rep(lang, each = length(ph)),
      phoible = ph,
      ausphon = au,
      stringsAsFactors = FALSE)
  )
}
```

```r
# Define some useful segment sets
stops <- c("p", "t", "\u0288", "t\u033a", "t\u032a", "\u0236", "k")
lenis_stops <- str_c(stops, "\u0349")
fortis_stops <-  str_c(stops, "\u0348")
lenis_continuants <-
  c("\u03b2", "\u027e", "\u027d", "\u027e\u033a", "ð", "", "\u0263")
vowels3 <- c("a", "i", "u")
vowels5 <- c("a", "i", "u", "e", "o")

## We begin with differing symbolizations of laminal consonants in
## over 60 languages.

# This set of languages has a minority pattern:
laminal_corresp_minority <-
  c("Nyawaygi", "Dharumbal", "Butchulla", "Ngaanyatjarra", "Tiwi")

# These languages follow the major pattern:
laminal_corresp_majority <-
  filter(ausphon, str_detect(phoneme, "\u033b"))$language %>%
  setdiff(laminal_corresp_minority)

eq <-
  # Initialise the dataframe
  NULL %>%

  # Implement the general laminal equivalences where phoible curled
  # symbols = ausphon laminal diacritic
  add_to_df(
    lang = laminal_corresp_majority,
    ph = c("j", "\u0234", "\u0235", "\u0236",
           "\u0236\u0348", "\u0236\u0349"),
    au = c("j\u033b", "l\u033b", "n\u033b", "t\u033b",
           "t\u033b\u0348", "t\u033b\u0349")) %>%

  # The exceptions mentioned above, where phoible dental = ausphon
  # laminal diacritic
  add_to_df(
    lang = laminal_corresp_minority,
    ph = c("j", "l\u032a", "n\u032a", "t\u032a",
           "t\u032a\u0349", "t\u032a\u0348"),
    au = c("j\u033b", "l\u033b", "n\u033b", "t\u033b",
           "t\u033b\u0348", "t\u033b\u0349")) %>%

## We continue for other segment types with simple, 1:1 symbol
## correspondences between the two datasets

  # Continuant/stop contrast versus lenis/fortis
  add_to_df(
    lang = "Adnyamathanha",
    ph = c(lenis_continuants[-6], stops[-6]),
    au = c(lenis_stops[-6], fortis_stops[-6])) %>%

  # Misc consonant phonemes
```

```r
  add_to_df("Dalabon", ph = "h", au = "\u0266") %>%
  add_to_df("Thaynakwithi", ph = "ð", au = "\u0291") %>%
  # flapped lateral
  add_to_df(c("Iwaidja", "Amurdak"),
            ph = "\u027a\u033a\u0320",
            au = "\u027a\u0322") %>%
  # Unicode correpondences
  add_to_df(c("Yaygir", "Dharumbal"),
            ph = "r\u033a\u0348",
            au = "r\u033a\u0325") %>%

  # Vowel phonemes
  add_to_df("Worrorra",
            ph = c("e", "o"),
            au = c("e\u02d0", "o\u02d0")) %>%
  add_to_df("Kurtjar",
            ph = c("i\u02d0", "u\u02d0"),
            au = c("e\u02d0", "o\u02d0")) %>%
  add_to_df("Matngele", ph = "\u0289", au = "\u0268") %>%
  add_to_df("Kok Nar", ph = "\u0275", au = "\u0289") %>%
  # Unicode correpondences
  add_to_df("Anguthimri",
            ph = c("a\u0303", "e\u0303", "i\u0303"),
            au = c("\u00e3", "\u1ebd", "\u0129"))
```

Compile 2-to-1 correspondences in the datasets

A number of the languages are accorded different phonemic analyses in Phoible (whose sources mainly are grammars) versus AusPhon (mainly wordlists and dictionaries). This can involve 2-to-1 correspondences. Here we compile dataframes of phoneme pairs conflated in either Phoible or AusPhon, relative to the other.

```r
## Instances where Phoible conflates oppositions in AusPhon

ph_conflates <-
  # initialise the dataframe
  NULL %>%

  # 2 vs 1 apical series of consonants
  add_to_df(
    lang = c("Oykangand", "Yorta Yorta"),
    ph = c("l\u033a", "l\u033a", "n\u033a", "n\u033a",
           "t\u033a", "t\u033a", "r\u033a", "\u0279\u033a",
           "t\u033a\u0349", "t\u033a\u0349",
           "t\u033a\u0348", "t\u033a\u0348"),
    au = c("l", "\u026d", "n", "\u0273", "t", "\u0288", "r", "\u027b",
           "t\u0349", "t\u0348", "\u0288\u0348", "\u0288\u0349")) %>%

  # 2 vs 1 laminal series of consonants
  add_to_df(
    lang = c("Gangulu"),
    ph = c("n\u033b", "n\u033b", "t\u033b", "t\u033b"),
    au = c("n\u032a", "\u0235", "t\u032a", "\u0236")) %>%

  # 2 vs 1 laminal glides
```

```r
add_to_df(
  lang = c("Unggumi"),
  ph = c("j", "j"),
  au = c("j", "j\u032a")) %>%

# 2 vs 1 laminal laterals
add_to_df(
  lang = c("Wubuy"),
  ph = c("l\u032a", "l\u032a"),
  au = c("\u0234", "l\u032a")) %>%

# 2 vs no laminal laterals
add_to_df(
  lang = c("Olkol", "Oykangand"),
  ph = c("l\u033a", "l\u033a"),
  au = c("\u0234", "l\u032a")) %>%

# 2 vs 1 apical laterals
add_to_df(
  lang = c("Emmi"),
  ph = c("l", "l"),
  au = c("l", "\u026d")) %>%

# 2 vs 1 vibrants
add_to_df(
  lang = c("Ngarinyin"),
  ph = c("r", "r"),
  au = c("r", "\u027e")) %>%

# 2 vs 1 series of stops
add_to_df(
  lang = c("Patjtjamalh", "Larrakia", "Erre",
           "Mengerrdji", "Urningangg", "Thaynakwithi"),
  ph = c(stops, stops),
  au = c(fortis_stops, lenis_stops)) %>%

# 2 vs 1 series of apical stops
add_to_df(
  lang = c("Nakara"),
  ph = c("t\u0348", "t\u0348"),
  au = c("t\u0348", "t\u0349")) %>%

# 2 vs 1 series of palatal stops
add_to_df(
  lang = c("Adnyamathanha"),
  ph = c("\u0236", "\u0236"),
  au = c("\u0236\u0348", "\u0236\u0349")) %>%

# velar glide
add_to_df(
  lang = c("Oykangand"),
  ph = c("k\u0349"),
  au = c("\u0270")) %>%
```

```r
  # 2 vs 1 vowel lengths
  add_to_df(
    lang = c("Matngele", "Larrakia", "Olkol",
             "Putijarra", "Ngarinyin", "Unggumi",
             "Yawijibaya"),
    ph = c(vowels5, vowels5),
    au = c(vowels5, str_c(vowels5, "\u02d0"))) %>%

  # 2 vs 1 length for the low vowel
  add_to_df(
    lang = c("Badimaya", "Ngawun", "Kariyarra",
             "Yorta Yorta"),
    ph = c("a", "a"),
    au = c("a", "a\u02d0"))


## Instances where AusPhon conflates oppositions in Phoible.

au_conflates <-
  # initialise the dataframe
  NULL %>%

  # 2 vs 1 apical series of consonants
  add_to_df(
    lang = c("Guugu Yimidhirr", "Bidyara", "Gangulu", "Oykangand"),
    ph = c("l", "\u026d", "n", "\u0273", "t", "\u0288", "r", "\u027b"),
    au = c("l\u033a", "l\u033a", "n\u033a", "n\u033a",
           "t\u033a", "t\u033a", "r\u033a", "\u0279\u033a")) %>%

  # 2 vs 1 laminal series of consonants
  add_to_df(
    lang = c("Murrinh-patha", "Nyiyaparli", "Yawijibaya"),
    ph = c("l\u032a", "\u0234", "n\u032a", "\u0235", "t\u032a",
           "\u0236", "t\u032a\u0349", "\u0236\u0349",
           "t\u032a\u0348", "\u0236\u0348"),
    au = c("\u0234", "\u0234", "\u0235", "\u0235", "\u0236",
           "\u0236", "\u0236\u0349", "\u0236\u0349",
           "\u0236\u0348", "\u0236\u0348")) %>%

  # 2 vs 1 series of stops
  add_to_df(
    lang = c("Alawa", "Bakanh", "Linngithigh"),
    ph = c(fortis_stops, lenis_stops),
    au = c(stops, stops)) %>%

  # frictive & lenis stops as (non)distinctive
  add_to_df(
    lang = c("Ogh Unyjan", "Olkol", "Oykangand"),
    ph = lenis_continuants,
    au = lenis_stops) %>%

  # frictive & plain stops as (non)distinctive
  add_to_df(
```

```r
    lang = c("Kok Nar", "Larrakia"),
    ph = lenis_continuants,
    au = stops) %>%

  # 2 vs 1 vowel lengths
  add_to_df(
    lang = "Gangulu",
    ph = c(vowels3, str_c(vowels3, "\u02d0")),
    au = c(vowels3, vowels3)) %>%

  # 2 vs 1 length for the low vowel
  add_to_df(
    lang = c("Yanyuwa"),
    ph = c("a", "a\u02d0"),
    au = c("a", "a")) %>%

  # 3 vs 2 vowel heights
  add_to_df(
    lang = "Yawijibaya",
    ph = c("i", "e", "o", "u"),
    au = c("i", "i", "u", "u"))
```

Harmonize datasets across the two databases

Now that we've established the symbol correspondences between the two databases, we harmonize them with one another, so all phonemes – if they are present – have a 1:1 correspondence with matching phonemes in the other dataset, using the same symbols.

```r
# Helper function
harmonize_matches = function(x,y) {
  left_join(x, y, by = c("language", "phoneme")) %>%
  mutate(phoneme =
    ifelse(is.na(new_phoneme), phoneme, new_phoneme)) %>%
  distinct() %>%
  select(-new_phoneme)
}

# Replace phonemes to represent matches correctly
colnames(eq)[2:3] <- c("new_phoneme", "phoneme")
colnames(au_conflates)[2:3] <- c("phoneme", "new_phoneme")
colnames(ph_conflates)[2:3] <- c("new_phoneme", "phoneme")

au_harmonized <-
  ausphon %>%
  distinct() %>%
  # Change au symbols to match phoible equivalences
  harmonize_matches(eq) %>%
  # Conflate au phonemes to match phoible
  harmonize_matches(ph_conflates)

ph_harmonized <-
  phoible_ER %>%
  distinct() %>%
  # Conflate ph phonemes to match ausphon
```

```r
harmonize_matches(au_conflates)
```

Find true discrepancies in the attested sets of phonemes

Now find the true discrepancies – which segments in Phoible are not found in the AusPhon data, even though we'd expect them to be, and vice-versa.

```r
# Helper function
get_discrepancies = function(x,y) {
  anti_join(x, y, by = c("language", "phoneme")) %>%
  arrange(language, phoneme) %>%
  group_by(language) %>%
  summarise(phonemes = str_c(phoneme, collapse = " "))
}

# Tabulate the discrepancies
in_au_not_ph <- get_discrepancies(au_harmonized, ph_harmonized)
in_ph_not_au <- get_discrepancies(ph_harmonized, au_harmonized)

if(nrow(in_au_not_ph) > 0) {
  kable(
    in_au_not_ph,
    col.names = c("Language", "In Ausphon but not Phoible"),
    align = "ll"
  )
}

if(nrow(in_ph_not_au) > 0) {
  kable(
    in_ph_not_au,
    col.names = c("Language", "In Phoible but not Ausphon"),
    align = "ll"
  )
}
```

Language	In Phoible but not Ausphon
Marra	e l̪ n̪
Emmi	ʈ̺ ʐ
Ngardily	uː

Evaluation

What we see is that all AusPhon phonemes are present in the Phoible data. Nearly all Phoible phonemes are represented in the Ausphon data, but a few are not: these are phonemes which did not appear in the sample that the AusPhon wordlist comprises. The Emmi wordlist is only 304 words long; the Ngardily list is 625 words. The Marra wordlist is longer, at 863 words. Each of its three missing phonemes are identified by Heath (1981: pp.10-11) as marginal with each appearing in no more than three known flora/fauna terms, which do not appear in the AusPhon data. Overall, given that we have examined 166 languages, this indicates that missed phonemes are rare, and are not contributing significantly to the results in our study.

S5. Tables of results

The tables below give the results of maximum likelihood estimation for each distribution type and each language variety. Each table contains estimated parameter values, goodness-of-fit (Kolmogorov-Smirnof statistic) and the p value from the bootstrapping procedure.

All tables contain a '**lex ID**' column giving the numerical identifier for the language variety in the Ausphonlex database and a '**language variety**' column giving the common name identifier in the Ausphonlex database. Tables S5.1–S5.8 all contain a 'p' column giving the p value estimated via the bootstrapping procedure and a '**goodness-of-fit**' column giving the Kolmogorov-Smirnov statistic, which quantifies the distance between the fitted distribution and the observed distribution of the data. The rows of each table are ordered by the goodness-of-fit, from best-fitting to worst-fitting.

Table S5.1 reports results for the power law distribution fitted to each language's full segmental inventory (with no x_{min} parameter). It gives the maximum likelihood estimate of the shape parameter, α and the number of contrastive phonological segments in that language variety's segmental inventory (in the 'inventory size' column).

Table S5.2 reports results for the power law distribution with the addition of the x_{min} parameter. Thus, in addition to the shape parameter, α, Table S5.2 also gives the maximum likelihood estimate of x_{min} for each language. The x_{min} parameter is a count number whereby segments that appear fewer than x_{min} times in the wordlist are disregarded. To make the figure comparable between languages, here we report x_{min} as a fraction of the total number of segments in a language's wordlist. The original x_{min} values in count form can be accessed via the results files in S5. The column 'n **segments**' reports the number of unique phonemes in that language's segmental inventory that are at or above x_{min} in frequency (in other words, the number of observations to which the distribution has been fitted) and '**frac. of inventory**' gives the fraction of that language's total segmental inventory to which the distribution has been fitted.

Table S5.3 reports results for the lognormal distribution fitted to each language's full segmental inventory. Maximum likelihood estimates for the *log* mean and *log* SD parameters are given. Table S5.4 reports results for the lognormal distribution with an x_{min} parameter included.

Tables S5.5 and S5.6 cover the exponential distribution, with and without x_{min} respectively. They report maximum likelihood estimates for the λ shape parameter. Likewise, Tables S5.7 and S5.8 cover the Poisson distribution, with and without x_{min}. The shape parameter for the Poisson distribution is also termed λ.

Lastly, Table S5.9 reports results of Vuong's (1989) likelihood ratio test. The test statistic, R, is the ratio of the log likelihood of the data given an exponential distribution model versus a lognormal distribution model (with no x_{min} parameters). The sign of R indicates which of the two models fits best (positive values indicate the exponential distribution is preferred, negative values for the lognormal distribution). Rows are ordered by R. The 'p' column gives a one-sided p value indicating whether the exponential distribution is favoured over the lognormal distribution to a statistically significant degree. The '**signif.**' column indicates whether the p value is considered statistically significant after Bonferroni correction.

Table S5.1. Power law distribution.

Lex ID	Language	Glottolog	α	Phonemes	p	Goodness-of-fit
865	Diyari	dier1241	1.69	25	0.270	0.15
866	Thirarri	dira1238	1.69	25	0.166	0.17
170	Wik-Ngathan	wikn1245	1.67	28	0.076	0.19
162	Adnyamathanha	adny1235	1.72	29	0.043	0.20
962	Gunya	guny1241	1.41	31	0.030	0.21
540	Ngiyambaa	wang1291	1.57	21	0.056	0.22
592	Muruwari	muru1266	1.64	25	0.031	0.23
821	Gumbaynggir	kumb1268	2.18	19	0.053	0.23
940	Yawijibaya	yawi1239	1.49	23	0.016	0.25
656	Kuugu Ya'u	kuuk1238	1.78	21	0.026	0.25
939	Unggumi	ungg1243	1.32	30	0.006	0.26
787	Jawoyn	djau1244	1.65	27	0.007	0.26
1043	Mbabaram	mbab1239	1.44	27	0.004	0.26
62	Dhay'yi	dhal1246	1.55	26	0.009	0.26
958	Margany	marg1253	1.35	30	0.004	0.27
611	Anguthimri	angu1242	1.27	37	0.003	0.27
737	Kukatj	guga1239	1.66	23	0.010	0.27
930	Purduna	burd1238	1.47	26	0.005	0.27
1042	Thaayorre	thay1249	1.51	26	0.012	0.27
63	Dharumbal	dhar1248	1.45	21	0.017	0.27
977	Warndarrang	wand1263	1.88	19	0.014	0.27
89	Bunuba	buna1275	1.37	24	0.016	0.27
949	Kok Nar	kokn1236	1.56	21	0.009	0.28
985	Yaygir	yayg1236	1.64	20	0.015	0.28
206	Wayilwan	wayi1238	1.65	21	0.011	0.28
13	Dyirbal	dyir1250	1.84	16	0.026	0.28
927	Tiwi	tiwi1244	1.49	20	0.026	0.28
1008	Yuwaalaraay	yuwa1242	1.48	21	0.018	0.28
495	Nyikina	nyig1240	1.59	20	0.014	0.29
979	Mawng	maun1240	1.52	22	0.014	0.29
983	Nhangu	yann1237	1.50	31	0.001	0.29
1032	Kuku Yalanji	kuku1273	1.91	16	0.026	0.29
740	Kugu Nganhcara	wikn1246	1.41	30	0.001	0.29
853	Lardil	lard1243	1.47	25	0.008	0.29
30	Duungidjawu	duun1241	1.52	23	0.003	0.30
1003	Murrinh-patha	murr1259	1.43	25	0.002	0.30
771	Kalkatungu	kalk1246	1.50	26	0.002	0.30
1002	Yintyingka	ayab1239	1.42	26	0.002	0.30
412	Ritharrngu	rita1239	1.52	31	0.000	0.30
835	Gugu Badhun	gugu1253	1.65	17	0.011	0.30
1007	Yuwaliyaay	yuwa1243	1.43	21	0.011	0.31
968	Ogh Angkula	ikar1243	1.58	24	0.001	0.31
1018	Djapu	djap1238	1.55	26	0.001	0.31
77	Dhangu	dhan1270	1.45	26	0.002	0.31
807	Gupapuyngu	gupa1247	1.53	31	0.000	0.31
929	Wanyjirra	wany1244	1.62	21	0.003	0.31
101	Butchulla	baty1234	1.29	24	0.006	0.32
475	Southern Paakintyi	darl1243	1.48	26	0.002	0.32
1012	Warrgamay	warr1255	1.43	19	0.010	0.32
919	Angkamuthi	angg1238	1.33	26	0.003	0.32
1009	Gamilaraay	gami1243	1.47	21	0.004	0.32

Lex ID	Language	Glottolog	α	Phonemes	p	Goodness-of-fit
427	Yidiny	yidi1250	1.58	19	0.007	0.32
978	Wotjobaluk	wotj1234	1.56	19	0.004	0.32
400	Thalanyji	dhal1245	1.35	26	0.002	0.33
200	Western Arrernte	west2441	1.49	25	0.001	0.33
91	Bularnu	bula1255	1.27	30	0.002	0.33
554	Ngarluma	ngar1287	1.54	23	0.001	0.34
1029	Martuthunira	mart1255	1.34	26	0.002	0.34
925	Larrakia	lara1258	1.22	29	0.003	0.34
546	Yanyuwa	yany1243	1.37	23	0.004	0.34
697	Kurrama	kurr1243	1.33	27	0.001	0.34
252	Warlmanpa	warl1255	1.29	28	0.002	0.34
265	Wardaman	ward1246	1.48	22	0.002	0.34
443	Payungu	bayu1240	1.32	26	0.001	0.34
790	Jaru	jaru1254	1.39	23	0.004	0.34
363	Yir Yoront	yiry1245	1.43	26	0.001	0.34
946	Kurtjar	gurd1238	1.54	28	0.000	0.34
926	Limilngan	nucl1327	1.28	24	0.002	0.35
642	Mangala	mang1383	1.34	21	0.005	0.35
5	Gangulu	gang1268	1.39	18	0.005	0.35
1040	Ngarinyin	ngar1284	1.16	27	0.044	0.35
1016	Tharrkari	dhar1247	1.31	32	0.000	0.35
105	Bardi	bard1255	1.56	24	0.000	0.35
269	Wangkumara	wong1246	1.31	30	0.000	0.35
778	Jiwarli	djiw1241	1.37	26	0.001	0.35
752	Kija	kitj1240	1.28	24	0.005	0.35
237	Warluwarra	warl1256	1.20	33	0.001	0.35
928	Bakanh	paka1251	1.30	26	0.001	0.35
911	Nyawaygi	nyaw1247	1.46	18	0.004	0.36
1026	Gidabal	gida1240	1.45	20	0.002	0.36
1006	Central Arrernte	mpar1238	1.52	25	0.000	0.36
81	Dalabon	ngal1292	1.31	29	0.003	0.36
204	Wemba Wemba	wemb1241	1.44	22	0.002	0.36
921	Gurr-Goni	gura1252	1.39	27	0.001	0.36
631	Yalarnnga	yala1262	1.37	24	0.001	0.36
800	Guwamu	guwa1243	1.30	21	0.003	0.36
1001	Ngandi	ngan1295	1.20	34	0.001	0.36
1025	Waalubal	band1358	1.43	20	0.003	0.36
996	Emmi	amii1238	1.31	31	0.000	0.36
943	Pintupi	pint1250	1.46	23	0.002	0.36
965	Oykangand	oyka1239	1.22	31	0.001	0.36
982	Nhirrpi	nhir1234	1.25	30	0.000	0.36
12	Erre	erre1238	1.23	27	0.001	0.36
847	Ngalakgan	ngal1293	1.31	27	0.001	0.36
957	Ngaanyatjarra	ngaa1240	1.52	23	0.001	0.37
417	Yindjibarndi	yind1247	1.48	25	0.000	0.37
905	Malkana	malg1242	1.31	25	0.000	0.37
519	Nukunu	nugu1241	1.31	28	0.000	0.37
288	Wangkatja	pini1245	1.48	23	0.000	0.37
857	Amurdak	amar1271	1.29	24	0.001	0.37
849	Waanyi	wany1247	1.30	20	0.006	0.37
94	Biri	biri1256	1.39	18	0.003	0.37
945	Wiri	biri1256	1.38	18	0.002	0.37

Lex ID	Language	Glottolog	α	Phonemes	p	Goodness-of-fit
1023	Wubuy	nung1290	1.30	26	0.001	0.38
118	Badimaya	badi1246	1.30	24	0.001	0.38
38	Djabugay	dyaa1242	1.32	19	0.005	0.38
31	Djinang	djin1253	1.36	25	0.000	0.38
838	Gooniyandi	goon1238	1.34	23	0.001	0.38
914	Yinhawangka	yinh1234	1.27	26	0.002	0.38
618	Mirniny	mirn1243	1.23	25	0.001	0.38
856	Patjtjamalh	wadj1254	1.20	27	0.001	0.38
935	Rembarrnga	remb1249	1.38	28	0.000	0.39
934	Urningangg	urni1239	1.24	27	0.000	0.39
845	Iwaidja	iwai1244	1.38	23	0.001	0.39
744	Koko Bera	gugu1254	1.54	21	0.000	0.39
621	Mengerrdji	mang1382	1.22	26	0.000	0.39
922	Nakara	naka1260	1.39	26	0.000	0.39
848	Wagiman	wage1238	1.30	27	0.000	0.39
852	Gurindji	guri1247	1.27	23	0.004	0.39
1011	Yorta Yorta	yort1237	1.23	24	0.001	0.40
650	Linngithigh	leni1238	1.23	29	0.000	0.40
1031	Ngadjunmaya	ngad1258	1.23	25	0.001	0.40
228	Warriyangga	wari1262	1.23	26	0.000	0.40
901	Guugu Yimidhirr	gugu1255	1.46	21	0.000	0.40
964	Olkol	ulku1238	1.18	29	0.002	0.40
762	Kariyarra	kari1304	1.27	24	0.000	0.40
606	Mudburra	mudb1240	1.23	23	0.001	0.40
760	Kartujarra	kart1247	1.28	23	0.001	0.41
85	Burarra	bura1267	1.44	26	0.000	0.41
534	Nhanda	nhan1238	1.22	33	0.000	0.41
565	Ngarinyman	ngar1235	1.23	23	0.002	0.41
967	Thaynakwithi	tyan1235	1.22	32	0.000	0.41
242	Warlpiri	warl1254	1.37	24	0.000	0.41
1024	Ngardily	west2437	1.30	23	0.000	0.41
1021	Watjarri	waja1257	1.29	26	0.000	0.41
433	Pitta Pitta	pitt1247	1.24	25	0.000	0.41
923	Matngele	madn1237	1.20	25	0.001	0.42
952	Ngawun	ngaw1240	1.23	22	0.000	0.42
841	Worrorra	woro1258	1.33	25	0.000	0.42
462	Panyjima	pany1241	1.21	25	0.000	0.42
1030	Putijarra	pudi1238	1.18	25	0.002	0.42
99	Bidyara	bidy1243	1.31	19	0.001	0.42
851	Bilinarra	bili1250	1.24	23	0.002	0.42
920	Yadhaykenu	yadh1237	1.26	25	0.000	0.42
917	Nyamal	nyam1271	1.19	24	0.002	0.43
645	Malyangapa	maly1234	1.25	24	0.000	0.43
734	Kukatja	kuka1246	1.33	23	0.001	0.43
493	Nyiyaparli	nija1241	1.24	22	0.001	0.43
1019	Walmajarri	walm1241	1.21	23	0.003	0.43
941	Wambaya	nucl1328	1.17	23	0.004	0.44
598	Yandruwandha	yand1253	1.20	30	0.000	0.44
305	Western Wakaya	waga1260	1.19	26	0.000	0.44
563	Ngarla	ngar1296	1.21	23	0.001	0.45
910	Nungali	nung1291	1.38	16	0.001	0.45
377	Umpila	umpi1239	1.25	22	0.000	0.45

**Lex ID**	**Language**	**Glottolog**	α	**Phonemes**	p	**Goodness-of-fit**
117	Wirangu	wira1265	1.29	23	0.000	0.45
767	Karajarri	kara1476	1.20	21	0.003	0.46
918	Atampaya	atam1239	1.21	25	0.000	0.46
113	Yulparija	yulp1239	1.19	23	0.002	0.46
966	Wik Mungkan	wikm1247	1.22	26	0.000	0.46
232	Warnman	wanm1242	1.25	23	0.000	0.47
972	Ogh Unyjan	kawa1290	1.21	25	0.000	0.47
915	Alawa	alaw1244	1.31	21	0.000	0.48
620	Miriwoong	miri1266	1.17	20	0.001	0.51
863	Marra	mara1385	1.18	20	0.001	0.52
507	Nyangumarta	nyan1301	1.23	21	0.000	0.53

Table S5.2. **Power law distribution with x_{min}.**

Lex ID	Language	Glottolog	α	x_{min}	Phonemes	Frac. of inventory	p	Goodness-of-fit
919	Angkamuthi	angg1238	2.39	99	16	0.62	0.990	0.08
1006	Central Arrernte	mpar1238	2.35	173	11	0.44	0.994	0.09
917	Nyamal	nyam1271	2.55	173	13	0.54	0.996	0.09
762	Kariyarra	kari1304	2.54	54	18	0.75	0.958	0.09
288	Wangkatja	pini1245	2.26	241	19	0.83	0.968	0.09
400	Thalanyji	dhal1245	2.33	85	18	0.69	0.952	0.09
228	Warriyangga	wari1262	2.37	47	15	0.58	0.947	0.09
1024	Ngardily	west2437	2.33	56	17	0.74	0.938	0.10
507	Nyangumarta	nyan1301	2.31	178	19	0.90	0.898	0.10
845	Iwaidja	iwai1244	2.76	393	13	0.57	0.984	0.10
778	Jiwarli	djiw1241	2.34	147	17	0.65	0.909	0.10
606	Mudburra	mudb1240	2.70	190	11	0.48	0.979	0.10
563	Ngarla	ngar1296	2.42	193	17	0.74	0.906	0.10
1030	Putijarra	pudi1238	2.45	151	19	0.76	0.858	0.10
170	Wik-Ngathan	wikn1245	3.77	249	12	0.43	0.972	0.11
760	Kartujarra	kart1247	2.35	147	12	0.52	0.946	0.11
972	Ogh Unyjan	kawa1290	3.09	76	15	0.60	0.907	0.11
1023	Wubuy	nung1290	2.46	200	14	0.54	0.928	0.11
1003	Murrinh-patha	murr1259	3.24	101	12	0.48	0.949	0.11
921	Gurr-Goni	gura1252	2.83	367	15	0.56	0.899	0.11
113	Yulparija	yulp1239	2.39	352	13	0.57	0.921	0.11
985	Yaygir	yayg1236	3.31	234	12	0.60	0.954	0.11
943	Pintupi	pint1250	2.29	635	18	0.78	0.791	0.11
1019	Walmajarri	walm1241	2.52	740	16	0.70	0.867	0.11
767	Karajarri	kara1476	2.67	433	11	0.52	0.933	0.12
957	Ngaanyatjarra	ngaa1240	2.34	249	19	0.83	0.743	0.12
162	Adnyamathanha	adny1235	2.39	205	18	0.62	0.722	0.12
519	Nukunu	nugu1241	2.20	31	19	0.68	0.540	0.12
967	Thaynakwithi	tyan1235	3.11	112	13	0.41	0.862	0.12
914	Yinhawangka	yinh1234	2.29	127	19	0.73	0.671	0.12
1040	Ngarinyin	ngar1284	3.34	1924	13	0.48	0.921	0.12
592	Muruwari	muru1266	2.35	184	15	0.60	0.825	0.12
475	Southern Paakintyi	darl1243	1.79	59	25	0.96	0.486	0.12
852	Gurindji	guri1247	2.76	1235	11	0.48	0.939	0.12
863	Marra	mara1385	2.71	179	12	0.60	0.884	0.12
849	Waanyi	wany1247	2.70	341	12	0.60	0.910	0.12
857	Amurdak	amar1271	2.61	85	11	0.46	0.880	0.12
656	Kuugu Ya'u	kuuk1238	2.58	182	14	0.67	0.798	0.12
740	Kugu Nganhcara	wikn1246	3.01	94	12	0.40	0.865	0.12
265	Wardaman	ward1246	2.73	338	15	0.68	0.748	0.12
77	Dhangu	dhan1270	2.39	53	17	0.65	0.671	0.12
91	Bularnu	bula1255	2.00	44	22	0.73	0.474	0.12
905	Malkana	malg1242	2.61	44	16	0.64	0.663	0.13
631	Yalarnnga	yala1262	2.45	98	14	0.58	0.791	0.13
915	Alawa	alaw1244	2.68	517	14	0.67	0.792	0.13
565	Ngarinyman	ngar1235	2.51	289	12	0.52	0.829	0.13
982	Nhirrpi	nhir1234	2.63	43	15	0.50	0.665	0.13
12	Erre	erre1238	3.36	100	11	0.41	0.881	0.13
929	Wanyjirra	wany1244	2.67	176	13	0.62	0.800	0.13
1011	Yorta Yorta	yort1237	2.91	84	9	0.38	0.910	0.13
865	Diyari	dier1241	2.24	43	13	0.52	0.582	0.13

Lex ID	Language	Glottolog	α	x_{min}	Phonemes	Frac. of inventory	p	Goodness-of-fit
1008	Yuwaalaraay	yuwa1242	4.22	530	10	0.48	0.940	0.13
866	Thirarri	dira1238	2.24	43	13	0.52	0.600	0.13
821	Gumbaynggir	kumb1268	3.01	74	13	0.68	0.653	0.13
5	Gangulu	gang1268	2.91	161	7	0.39	0.965	0.13
968	Ogh Angkula	ikar1243	3.75	128	10	0.42	0.867	0.13
621	Mengerrdji	mang1382	2.99	85	13	0.50	0.778	0.13
204	Wemba Wemba	wemb1241	2.98	266	13	0.59	0.784	0.13
117	Wirangu	wira1265	2.26	55	17	0.74	0.574	0.13
81	Dalabon	ngal1292	3.21	679	15	0.52	0.604	0.13
952	Ngawun	ngaw1240	2.50	59	12	0.55	0.775	0.13
1012	Warrgamay	warr1255	2.50	170	13	0.68	0.794	0.13
206	Wayilwan	wayi1238	3.77	203	11	0.52	0.862	0.13
493	Nyiyaparli	nija1241	2.59	48	12	0.55	0.747	0.13
85	Burarra	bura1267	2.34	331	20	0.77	0.385	0.13
1031	Ngadjunmaya	ngad1258	2.82	159	13	0.52	0.752	0.14
642	Mangala	mang1383	2.76	284	11	0.52	0.832	0.14
31	Djinang	djin1253	2.29	319	18	0.72	0.484	0.14
958	Margany	marg1253	2.49	50	14	0.47	0.677	0.14
650	Linngithigh	leni1238	2.46	58	18	0.62	0.413	0.14
966	Wik Mungkan	wikm1247	2.55	673	16	0.62	0.584	0.14
930	Purduna	burd1238	2.52	111	15	0.58	0.643	0.14
38	Djabugay	dyaa1242	3.03	271	10	0.53	0.887	0.14
101	Butchulla	baty1234	2.75	85	15	0.62	0.639	0.14
62	Dhay'yi	dhal1246	2.25	149	17	0.65	0.537	0.14
554	Ngarluma	ngar1287	2.41	133	18	0.78	0.502	0.14
848	Wagiman	wage1238	2.93	271	15	0.56	0.645	0.14
901	Guugu Yimidhirr	gugu1255	2.71	47	15	0.71	0.593	0.14
734	Kukatja	kuka1246	2.31	742	11	0.48	0.744	0.14
598	Yandruwandha	yand1253	2.28	175	9	0.30	0.767	0.14
1029	Martuthunira	mart1255	2.55	170	15	0.58	0.620	0.14
946	Kurtjar	gurd1238	3.47	194	16	0.57	0.551	0.14
232	Warnman	wanm1242	2.21	126	17	0.74	0.390	0.14
443	Payungu	bayu1240	2.31	84	19	0.73	0.428	0.14
1043	Mbabaram	mbab1239	3.45	46	11	0.41	0.699	0.14
807	Gupapuyngu	gupa1247	2.18	212	24	0.77	0.270	0.14
841	Worrorra	woro1258	2.77	69	12	0.48	0.604	0.14
928	Bakanh	paka1251	2.65	78	16	0.62	0.507	0.14
996	Emmi	amii1238	2.35	43	16	0.52	0.347	0.14
1002	Yintyingka	ayab1239	2.38	60	16	0.62	0.528	0.14
1032	Kuku Yalanji	kuku1273	2.45	334	15	0.94	0.630	0.14
242	Warlpiri	warl1254	2.22	719	18	0.75	0.403	0.14
744	Koko Bera	gugu1254	3.88	350	10	0.48	0.832	0.14
851	Bilinarra	bili1250	2.29	240	17	0.74	0.517	0.14
377	Umpila	umpi1239	2.53	130	15	0.68	0.609	0.14
534	Nhanda	nhan1238	2.33	53	20	0.61	0.278	0.14
433	Pitta Pitta	pitt1247	2.22	82	16	0.64	0.486	0.14
89	Bunuba	buna1275	2.81	141	13	0.54	0.709	0.14
697	Kurrama	kurr1243	2.22	101	17	0.63	0.409	0.14
1009	Gamilaraay	gami1243	4.08	288	7	0.33	0.933	0.14
99	Bidyara	bidy1243	2.25	107	15	0.79	0.529	0.14
934	Urningangg	urni1239	3.75	132	8	0.30	0.795	0.15
835	Gugu Badhun	gugu1253	2.61	102	12	0.71	0.701	0.15

Lex ID	Language	Glottolog	α	x_{min}	Phonemes	Frac. of inventory	p	Goodness-of-fit
462	Panyjima	pany1241	2.23	68	14	0.56	0.432	0.15
618	Mirniny	mirn1243	2.47	70	9	0.36	0.683	0.15
918	Atampaya	atam1239	2.48	80	13	0.52	0.474	0.15
63	Dharumbal	dhar1248	3.01	158	8	0.38	0.873	0.15
771	Kalkatungu	kalk1246	1.97	114	21	0.81	0.208	0.15
979	Mawng	maun1240	2.80	723	11	0.50	0.704	0.15
269	Wangkumara	wong1246	1.93	47	24	0.80	0.160	0.15
417	Yindjibarndi	yind1247	2.30	94	18	0.72	0.294	0.15
941	Wambaya	nucl1328	2.46	345	12	0.52	0.599	0.15
1021	Watjarri	waja1257	2.33	129	19	0.73	0.229	0.15
237	Warluwarra	warl1256	2.32	160	14	0.42	0.432	0.15
1016	Tharrkari	dhar1247	2.56	60	13	0.41	0.404	0.15
925	Larrakia	lara1258	2.84	206	8	0.28	0.754	0.15
1007	Yuwaliyaay	yuwa1243	4.67	627	9	0.43	0.846	0.15
611	Anguthimri	angu1242	2.36	41	20	0.54	0.217	0.15
1018	Djapu	djap1238	2.32	126	16	0.62	0.322	0.15
977	Warndarrang	wand1263	2.82	126	6	0.32	0.857	0.15
920	Yadhaykenu	yadh1237	2.41	74	15	0.60	0.404	0.16
940	Yawijibaya	yawi1239	2.79	55	12	0.52	0.539	0.16
838	Gooniyandi	goon1238	2.32	216	17	0.74	0.301	0.16
200	Western Arrernte	west2441	2.88	73	11	0.44	0.516	0.16
939	Unggumi	ungg1243	2.67	61	15	0.50	0.399	0.16
252	Warlmanpa	warl1255	2.37	236	8	0.29	0.632	0.16
645	Malyangapa	maly1234	2.03	20	19	0.79	0.131	0.16
540	Ngiyambaa	wang1291	3.94	121	6	0.29	0.771	0.16
118	Badimaya	badi1246	2.62	106	10	0.42	0.602	0.16
949	Kok Nar	kokn1236	3.11	86	13	0.62	0.486	0.16
787	Jawoyn	djau1244	3.73	372	14	0.52	0.378	0.16
978	Wotjobaluk	wotj1234	2.90	154	12	0.63	0.563	0.16
847	Ngalakgan	ngal1293	3.84	229	11	0.41	0.495	0.16
1026	Gidabal	gida1240	3.35	364	12	0.60	0.547	0.16
427	Yidiny	yidi1250	3.66	659	6	0.32	0.806	0.16
752	Kija	kitj1240	3.20	481	10	0.42	0.571	0.16
983	Nhangu	yann1237	2.51	275	14	0.45	0.295	0.16
926	Limilngan	nucl1327	2.07	49	16	0.67	0.292	0.16
105	Bardi	bard1255	2.07	60	22	0.92	0.108	0.16
923	Matngele	madn1237	3.96	222	10	0.40	0.575	0.17
495	Nyikina	nyig1240	2.29	237	14	0.70	0.311	0.17
1001	Ngandi	ngan1295	3.04	199	13	0.38	0.211	0.17
910	Nungali	nung1291	3.02	211	6	0.38	0.707	0.17
856	Patjtjamalh	wadj1254	3.29	222	13	0.48	0.442	0.17
911	Nyawaygi	nyaw1247	3.27	220	6	0.33	0.834	0.17
945	Wiri	biri1256	3.23	131	7	0.39	0.749	0.17
1025	Waalubal	band1358	3.32	363	12	0.60	0.459	0.17
412	Ritharrngu	rita1239	2.81	420	6	0.19	0.484	0.17
922	Nakara	naka1260	2.19	122	20	0.77	0.056	0.18
1042	Thaayorre	thay1249	5.48	1381	7	0.27	0.838	0.18
935	Rembarrnga	remb1249	3.58	263	9	0.32	0.462	0.18
853	Lardil	lard1243	3.55	663	12	0.48	0.441	0.18
927	Tiwi	tiwi1244	2.27	522	14	0.70	0.272	0.18
737	Kukatj	guga1239	4.67	295	8	0.35	0.555	0.19
620	Miriwoong	miri1266	4.07	952	8	0.40	0.532	0.19

Lex ID	Language	Glottolog	α	x_{min}	Phonemes	Frac. of inventory	p	Goodness-of-fit
94	Biri	biri1256	2.24	113	13	0.72	0.213	0.19
962	Gunya	guny1241	1.51	9	29	0.94	0.011	0.19
964	Olkol	ulku1238	2.09	164	21	0.72	0.040	0.19
30	Duungidjawu	duun1241	2.65	89	14	0.61	0.085	0.20
965	Oykangand	oyka1239	2.34	171	19	0.61	0.031	0.20
546	Yanyuwa	yany1243	1.96	216	18	0.78	0.029	0.21
305	Western Wakaya	waga1260	2.72	342	6	0.23	0.358	0.21
800	Guwamu	guwa1243	2.21	55	16	0.76	0.047	0.21
363	Yir Yoront	yiry1245	6.14	452	9	0.35	0.365	0.21
13	Dyirbal	dyir1250	3.09	171	11	0.69	0.126	0.22
790	Jaru	jaru1254	3.12	551	12	0.52	0.092	0.22

Table S5.3. Lognormal distribution.

Lex ID	Language	Glottolog	*log* mean	*log* SD	Phonemes	*p*	Goodness-of-fit
866	Thirarri	dira1238	3.20	1.35	25	0.995	0.06
865	Diyari	dier1241	3.17	1.36	25	0.994	0.06
62	Dhay'yi	dhal1246	5.13	1.06	26	0.967	0.07
771	Kalkatungu	kalk1246	5.33	1.09	26	0.965	0.07
996	Emmi	amii1238	3.64	1.13	31	0.832	0.08
656	Kuugu Ya'u	kuuk1238	5.28	0.85	21	0.958	0.08
598	Yandruwandha	yand1253	4.41	1.42	30	0.847	0.08
821	Gumbaynggir	kumb1268	4.37	0.64	19	0.953	0.08
1018	Djapu	djap1238	4.94	0.98	26	0.879	0.08
427	Yidiny	yidi1250	6.01	0.77	19	0.969	0.09
475	Southern Paakintyi	darl1243	5.16	1.10	26	0.775	0.09
200	Western Arrernte	west2441	4.01	0.94	25	0.721	0.10
962	Gunya	guny1241	3.68	1.58	31	0.510	0.10
269	Wangkumara	wong1246	4.47	1.21	30	0.592	0.10
77	Dhangu	dhan1270	4.08	1.00	26	0.648	0.10
237	Warluwarra	warl1256	4.38	1.62	33	0.504	0.10
546	Yanyuwa	yany1243	5.96	1.13	23	0.772	0.10
63	Dharumbal	dhar1248	4.23	1.31	21	0.761	0.10
162	Adnyamathanha	adny1235	5.16	1.14	29	0.517	0.10
5	Gangulu	gang1268	4.67	1.01	18	0.862	0.10
94	Biri	biri1256	5.02	1.02	18	0.867	0.10
740	Kugu Nganhcara	wikn1246	3.85	1.22	30	0.436	0.10
540	Ngiyambaa	wang1291	3.90	1.04	21	0.701	0.10
433	Pitta Pitta	pitt1247	4.52	1.25	25	0.613	0.11
982	Nhirrpi	nhir1234	3.39	1.29	30	0.394	0.11
1032	Kuku Yalanji	kuku1273	6.30	0.71	16	0.841	0.11
204	Wemba Wemba	wemb1241	5.53	0.86	22	0.655	0.11
592	Muruwari	muru1266	5.00	1.18	25	0.447	0.11
983	Nhangu	yann1237	5.17	1.16	31	0.300	0.11
117	Wirangu	wira1265	4.34	1.07	23	0.553	0.12
979	Mawng	maun1240	6.24	1.07	22	0.566	0.12
920	Yadhaykenu	yadh1237	4.25	1.20	25	0.479	0.12
935	Rembarrnga	remb1249	4.99	0.98	28	0.389	0.12
1016	Tharrkari	dhar1247	3.66	1.19	32	0.254	0.12
744	Koko Bera	gugu1254	5.71	0.63	21	0.612	0.12
105	Bardi	bard1255	4.79	0.94	24	0.459	0.12
918	Atampaya	atam1239	4.22	1.24	25	0.449	0.12
901	Guugu Yimidhirr	gugu1255	4.03	0.80	21	0.540	0.12
968	Ogh Angkula	ikar1243	4.50	0.78	24	0.435	0.12
927	Tiwi	tiwi1244	6.41	1.11	20	0.580	0.12
945	Wiri	biri1256	4.34	1.05	18	0.654	0.12
1006	Central Arrernte	mpar1238	5.15	0.90	25	0.399	0.12
412	Ritharrngu	rita1239	5.09	1.17	31	0.222	0.12
495	Nyikina	nyig1240	5.61	1.05	20	0.550	0.12
1043	Mbabaram	mbab1239	3.16	1.11	27	0.242	0.12
1002	Yintyingka	ayab1239	4.04	1.14	26	0.302	0.12
265	Wardaman	ward1246	5.94	0.86	22	0.476	0.12
645	Malyangapa	maly1234	3.51	1.19	24	0.340	0.13
835	Gugu Badhun	gugu1253	4.76	0.88	17	0.572	0.13
946	Kurtjar	gurd1238	5.15	0.76	28	0.229	0.13
737	Kukatj	guga1239	5.08	0.81	23	0.330	0.13

Lex ID	Language	Glottolog	*log* mean	*log* SD	Phonemes	*p*	Goodness-of-fit
925	Larrakia	lara1258	4.08	1.71	29	0.208	0.13
958	Margany	marg1253	3.21	1.53	30	0.140	0.13
534	Nhanda	nhan1238	3.95	1.25	33	0.140	0.13
1029	Martuthunira	mart1255	4.96	1.22	26	0.269	0.13
807	Gupapuyngu	gupa1247	5.66	1.10	31	0.140	0.13
1023	Wubuy	nung1290	5.02	1.35	26	0.239	0.13
919	Angkamuthi	angg1238	4.52	1.25	26	0.226	0.13
1042	Thaayorre	thay1249	6.45	0.90	26	0.236	0.13
845	Iwaidja	iwai1244	5.78	1.06	23	0.305	0.14
631	Yalarnnga	yala1262	4.36	1.34	24	0.227	0.14
922	Nakara	naka1260	5.19	1.07	26	0.204	0.14
30	Duungidjawu	duun1241	4.37	1.04	23	0.237	0.14
841	Worrorra	woro1258	3.96	1.10	25	0.193	0.14
91	Bularnu	bula1255	4.10	1.42	30	0.104	0.14
170	Wik-Ngathan	wikn1245	4.94	0.94	28	0.111	0.14
966	Wik Mungkan	wikm1247	6.50	1.18	26	0.195	0.14
978	Wotjobaluk	wotj1234	4.98	0.89	19	0.349	0.14
915	Alawa	alaw1244	6.37	0.96	21	0.322	0.14
930	Purduna	burd1238	4.42	1.26	26	0.124	0.14
118	Badimaya	badi1246	4.30	1.19	24	0.186	0.15
99	Bidyara	bidy1243	5.06	1.07	19	0.343	0.15
621	Mengerrdji	mang1382	3.93	1.36	26	0.137	0.15
1009	Gamilaraay	gami1243	5.07	0.98	21	0.234	0.15
697	Kurrama	kurr1243	4.51	1.45	27	0.108	0.15
519	Nukunu	nugu1241	3.62	1.23	28	0.087	0.15
952	Ngawun	ngaw1240	3.80	1.32	22	0.210	0.15
929	Wanyjirra	wany1244	5.10	0.99	21	0.205	0.15
926	Limilngan	nucl1327	3.93	1.49	24	0.146	0.15
377	Umpila	umpi1239	4.96	1.14	22	0.217	0.15
554	Ngarluma	ngar1287	5.16	1.00	23	0.154	0.15
1003	Murrinh-patha	murr1259	4.14	1.04	25	0.114	0.15
921	Gurr-Goni	gura1252	5.73	1.04	27	0.093	0.15
642	Mangala	mang1383	5.46	1.08	21	0.218	0.15
13	Dyirbal	dyir1250	5.10	0.80	16	0.327	0.15
85	Burarra	bura1267	6.16	0.96	26	0.102	0.15
928	Bakanh	paka1251	4.24	1.15	26	0.095	0.15
787	Jawoyn	djau1244	5.56	0.89	27	0.062	0.16
1012	Warrgamay	warr1255	5.18	1.07	19	0.219	0.16
1024	Ngardily	west2437	4.26	1.16	23	0.130	0.16
790	Jaru	jaru1254	5.87	1.21	23	0.125	0.16
400	Thalanyji	dhal1245	4.52	1.23	26	0.076	0.16
852	Gurindji	guri1247	6.67	1.38	23	0.133	0.16
985	Yaygir	yayg1236	5.28	0.86	20	0.152	0.16
1025	Waalubal	band1358	5.76	0.88	20	0.182	0.16
1026	Gidabal	gida1240	5.76	0.86	20	0.178	0.16
363	Yir Yoront	yiry1245	5.48	0.88	26	0.068	0.16
972	Ogh Unyjan	kawa1290	4.15	1.10	25	0.076	0.16
939	Unggumi	ungg1243	3.46	1.47	30	0.023	0.16
848	Wagiman	wage1238	5.31	1.18	27	0.055	0.16
957	Ngaanyatjarra	ngaa1240	5.87	1.00	23	0.086	0.16
943	Pintupi	pint1250	6.73	1.13	23	0.077	0.17
838	Gooniyandi	goon1238	5.57	1.17	23	0.083	0.17

Lex ID	Language	Glottolog	*log* mean	*log* SD	Phonemes	p	Goodness-of-fit
800	Guwamu	guwa1243	4.28	1.20	21	0.113	0.17
905	Malkana	malg1242	3.68	1.21	25	0.051	0.17
853	Lardil	lard1243	5.94	1.13	25	0.043	0.17
228	Warriyangga	wari1262	3.74	1.31	26	0.041	0.17
752	Kija	kitj1240	5.58	1.24	24	0.065	0.17
89	Bunuba	buna1275	4.48	1.31	24	0.044	0.17
911	Nyawaygi	nyaw1247	4.79	1.06	18	0.131	0.17
1008	Yuwaalaraay	yuwa1242	5.66	1.10	21	0.072	0.18
1031	Ngadjunmaya	ngad1258	4.69	1.32	25	0.044	0.18
618	Mirniny	mirn1243	3.84	1.28	25	0.038	0.18
288	Wangkatja	pini1245	5.84	1.10	23	0.047	0.18
760	Kartujarra	kart1247	4.88	1.27	23	0.054	0.18
965	Oykangand	oyka1239	4.88	1.54	31	0.009	0.18
611	Anguthimri	angu1242	3.05	1.74	37	0.001	0.18
443	Payungu	bayu1240	4.57	1.24	26	0.023	0.18
417	Yindjibarndi	yind1247	4.79	1.05	25	0.021	0.18
977	Warndarrang	wand1263	4.48	0.81	19	0.055	0.18
31	Djinang	djin1253	5.92	1.24	25	0.023	0.18
940	Yawijibaya	yawi1239	3.18	1.49	23	0.017	0.19
949	Kok Nar	kokn1236	4.25	0.96	21	0.032	0.19
857	Amurdak	amar1271	3.84	1.57	24	0.021	0.19
1007	Yuwaliyaay	yuwa1243	5.76	1.14	21	0.040	0.19
767	Karajarri	kara1476	5.85	1.35	21	0.042	0.19
242	Warlpiri	warl1254	6.82	1.27	24	0.019	0.19
206	Wayilwan	wayi1238	4.87	0.95	21	0.019	0.20
847	Ngalakgan	ngal1293	4.90	1.15	27	0.008	0.20
1011	Yorta Yorta	yort1237	3.78	1.41	24	0.012	0.20
462	Panyjima	pany1241	4.14	1.45	25	0.007	0.20
734	Kukatja	kuka1246	6.51	1.21	23	0.015	0.20
1040	Ngarinyin	ngar1284	6.52	1.94	27	0.012	0.20
12	Erre	erre1238	3.75	1.53	27	0.003	0.21
914	Yinhawangka	yinh1234	4.96	1.40	26	0.004	0.21
1001	Ngandi	ngan1295	4.41	1.68	34	0.001	0.21
38	Djabugay	dyaa1242	5.24	1.22	19	0.020	0.21
565	Ngarinyman	ngar1235	5.30	1.55	23	0.008	0.21
81	Dalabon	ngal1292	6.04	1.30	29	0.002	0.21
778	Jiwarli	djiw1241	5.00	1.26	26	0.003	0.22
650	Linngithigh	leni1238	3.81	1.55	29	0.001	0.22
762	Kariyarra	kari1304	4.08	1.25	24	0.003	0.22
863	Marra	mara1385	5.12	1.33	20	0.011	0.22
305	Western Wakaya	waga1260	4.83	1.64	26	0.003	0.22
934	Urningangg	urni1239	3.65	1.81	27	0.001	0.22
910	Nungali	nung1291	5.09	0.95	16	0.028	0.23
606	Mudburra	mudb1240	4.80	1.49	23	0.002	0.23
493	Nyiyaparli	nija1241	3.66	1.37	22	0.002	0.23
252	Warlmanpa	warl1255	3.90	2.27	28	0.000	0.23
964	Olkol	ulku1238	5.05	1.91	29	0.001	0.23
856	Patjtjamalh	wadj1254	4.43	1.95	27	0.001	0.24
1021	Watjarri	waja1257	4.96	1.37	26	0.000	0.24
851	Bilinarra	bili1250	5.45	1.66	23	0.002	0.24
967	Thaynakwithi	tyan1235	4.10	1.55	32	0.000	0.24
563	Ngarla	ngar1296	5.27	1.56	23	0.001	0.24

Lex ID	Language	Glottolog	*log* mean	*log* SD	Phonemes	p	Goodness-of-fit
941	Wambaya	nucl1328	5.40	1.78	23	0.002	0.25
507	Nyangumarta	nyan1301	5.68	1.17	21	0.002	0.25
917	Nyamal	nyam1271	4.57	1.96	24	0.001	0.25
849	Waanyi	wany1247	5.32	1.70	20	0.002	0.25
620	Miriwoong	miri1266	6.27	1.44	20	0.002	0.26
1019	Walmajarri	walm1241	6.39	1.77	23	0.002	0.26
923	Matngele	madn1237	4.45	1.82	25	0.000	0.27
101	Butchulla	baty1234	3.74	1.73	24	0.000	0.27
232	Warnman	wanm1242	4.95	1.59	23	0.001	0.28
1030	Putijarra	pudi1238	4.88	1.75	25	0.000	0.29
113	Yulparija	yulp1239	5.57	1.76	23	0.001	0.30

Table S5.4. Lognormal distribution with x_{min}.

Lex ID	Language	Glottolog	*log* mean	*log* SD	x_{min}	Phonemes	Frac. of inventory	p	G[illegible]
996	Emmi	amii1238	3.76	0.97	7	29	0.94	1.000	[illegible]
866	Thirarri	dira1238	3.20	1.35	11	25	1.00	0.994	[illegible]
865	Diyari	dier1241	3.17	1.36	11	25	1.00	0.992	[illegible]
966	Wik Mungkan	wikm1247	6.76	0.76	258	22	0.85	0.997	[illegible]
565	Ngarinyman	ngar1235	5.67	0.89	115	19	0.83	0.999	[illegible]
771	Kalkatungu	kalk1246	5.18	1.12	84	23	0.88	0.986	[illegible]
697	Kurrama	kurr1243	4.90	0.97	45	22	0.81	0.981	[illegible]
62	Dhay'yi	dhal1246	5.13	1.06	32	26	1.00	0.935	[illegible]
841	Worrorra	woro1258	3.97	0.94	22	22	0.88	0.945	[illegible]
475	Southern Paakintyi	darl1243	4.74	1.32	69	22	0.85	0.945	[illegible]
656	Kuugu Ya'u	kuuk1238	4.74	1.01	154	16	0.76	0.994	[illegible]
598	Yandruwandha	yand1253	4.42	1.32	10	29	0.97	0.836	[illegible]
1023	Wubuy	nung1290	5.38	0.94	27	23	0.88	0.940	[illegible]
915	Alawa	alaw1244	6.25	0.83	253	20	0.95	0.976	[illegible]
85	Burarra	bura1267	5.51	1.12	274	22	0.85	0.944	[illegible]
269	Wangkumara	wong1246	4.51	1.12	23	27	0.90	0.797	[illegible]
928	Bakanh	paka1251	4.63	0.68	34	21	0.81	0.925	[illegible]
952	Ngawun	ngaw1240	3.88	1.00	35	16	0.73	0.980	[illegible]
265	Wardaman	ward1246	6.13	0.65	157	19	0.86	0.962	[illegible]
744	Koko Bera	gugu1254	5.78	0.51	149	19	0.90	0.958	[illegible]
857	Amurdak	amar1271	4.19	0.99	31	19	0.79	0.944	[illegible]
917	Nyamal	nyam1271	4.17	1.25	127	16	0.67	0.977	[illegible]
5	Gangulu	gang1268	4.86	0.83	42	15	0.83	0.982	[illegible]
1018	Djapu	djap1238	5.01	0.91	30	25	0.96	0.815	[illegible]
767	Karajarri	kara1476	5.72	0.98	233	17	0.81	0.976	[illegible]
821	Gumbaynggir	kumb1268	4.37	0.64	41	19	1.00	0.920	[illegible]
1024	Ngardily	west2437	1.62	1.68	56	17	0.74	0.954	[illegible]
921	Gurr-Goni	gura1252	5.88	0.80	173	22	0.81	0.872	[illegible]
12	Erre	erre1238	3.78	0.81	100	11	0.41	0.996	[illegible]
925	Larrakia	lara1258	5.11	0.83	38	18	0.62	0.896	[illegible]
427	Yidiny	yidi1250	5.92	0.79	163	18	0.95	0.947	[illegible]
926	Limilngan	nucl1327	4.31	1.07	10	21	0.88	0.834	[illegible]
851	Bilinarra	bili1250	5.76	0.94	162	19	0.83	0.920	[illegible]
934	Urningangg	urni1239	4.59	0.69	37	18	0.67	0.874	[illegible]
412	Ritharrngu	rita1239	5.51	0.84	48	26	0.84	0.679	[illegible]
1006	Central Arrernte	mpar1238	-2.37	2.62	156	12	0.48	0.958	[illegible]
540	Ngiyambaa	wang1291	4.58	0.61	47	12	0.57	0.941	[illegible]
288	Wangkatja	pini1245	1.57	2.06	241	19	0.83	0.927	[illegible]
31	Djinang	djin1253	5.32	1.19	266	20	0.80	0.866	[illegible]
620	Miriwoong	miri1266	6.60	0.65	189	18	0.90	0.918	[illegible]
495	Nyikina	nyig1240	5.87	0.85	76	18	0.90	0.900	[illegible]
606	Mudburra	mudb1240	4.29	1.17	120	16	0.70	0.913	[illegible]
621	Mengerrdji	mang1382	4.71	0.57	46	16	0.62	0.881	[illegible]
863	Marra	mara1385	3.96	1.17	156	14	0.70	0.957	[illegible]
1002	Yintyingka	ayab1239	4.45	0.80	32	19	0.73	0.729	[illegible]
918	Atampaya	atam1239	4.27	1.04	14	24	0.96	0.678	[illegible]
946	Kurtjar	gurd1238	5.32	0.57	66	25	0.89	0.618	[illegible]
923	Matngele	madn1237	5.28	0.56	80	19	0.76	0.828	[illegible]
237	Warluwarra	warl1256	4.88	1.18	22	26	0.79	0.504	[illegible]
941	Wambaya	nucl1328	5.66	1.04	155	18	0.78	0.866	[illegible]
1012	Warrgamay	warr1255	5.46	0.77	79	16	0.84	0.920	[illegible]

Lex ID	Language	Glottolog	*log* mean	*log* SD	x_{min}	Phonemes	Frac. of inventory	p	G
740	Kugu Nganhcara	wikn1246	2.59	1.19	94	12	0.40	0.892	0
170	Wik-Ngathan	wikn1245	5.65	0.46	145	16	0.57	0.833	0
919	Angkamuthi	angg1238	-3.84	2.65	99	16	0.62	0.864	0
200	Western Arrernte	west2441	4.00	0.93	18	23	0.92	0.547	0
982	Nhirrpi	nhir1234	3.57	1.08	4	28	0.93	0.383	0
204	Wemba Wemba	wemb1241	5.61	0.75	60	21	0.95	0.739	0
962	Gunya	guny1241	4.58	1.04	27	20	0.65	0.549	0
752	Kija	kitj1240	5.93	0.74	269	16	0.67	0.888	0
228	Warriyangga	wari1262	2.31	1.48	35	19	0.73	0.726	0
507	Nyangumarta	nyan1301	2.62	1.73	178	19	0.90	0.795	0
77	Dhangu	dhan1270	4.08	1.00	7	26	1.00	0.447	0
760	Kartujarra	kart1247	5.16	0.90	21	21	0.91	0.670	0
377	Umpila	umpi1239	5.05	0.86	37	21	0.95	0.670	0
1030	Putijarra	pudi1238	1.60	1.79	151	19	0.76	0.806	0
847	Ngalakgan	ngal1293	5.25	0.66	53	23	0.85	0.540	0
94	Biri	biri1256	5.31	0.77	70	14	0.78	0.879	0
631	Yalarnnga	yala1262	4.67	1.03	7	22	0.92	0.591	0
546	Yanyuwa	yany1243	5.96	1.13	27	23	1.00	0.600	0
63	Dharumbal	dhar1248	4.40	1.18	10	20	0.95	0.645	0
650	Linngithigh	leni1238	4.41	0.72	21	24	0.83	0.485	0
113	Yulparija	yulp1239	5.61	1.08	242	16	0.70	0.818	0
778	Jiwarli	djiw1241	0.99	2.02	153	16	0.62	0.863	0
945	Wiri	biri1256	4.36	0.89	57	13	0.72	0.908	0
81	Dalabon	ngal1292	6.79	0.47	391	19	0.66	0.663	0
117	Wirangu	wira1265	3.94	1.17	27	22	0.96	0.570	0
807	Gupapuyngu	gupa1247	4.95	1.23	212	24	0.77	0.505	0
99	Bidyara	bidy1243	4.16	1.20	107	15	0.79	0.835	0
162	Adnyamathanha	adny1235	5.16	1.14	63	29	1.00	0.334	0
91	Bularnu	bula1255	1.96	1.88	44	22	0.73	0.517	0
30	Duungidjawu	duun1241	4.70	0.74	19	20	0.87	0.584	0
979	Mawng	maun1240	6.48	0.88	122	20	0.91	0.619	0
848	Wagiman	wage1238	5.70	0.72	53	23	0.85	0.478	0
935	Rembarrnga	remb1249	5.14	0.80	28	26	0.93	0.339	0
400	Thalanyji	dhal1245	-1.73	2.38	85	18	0.69	0.689	0
958	Margany	marg1253	3.59	1.15	62	9	0.30	0.836	0
957	Ngaanyatjarra	ngaa1240	2.71	1.75	249	19	0.83	0.727	0
433	Pitta Pitta	pitt1247	4.52	1.25	2	25	1.00	0.374	0
645	Malyangapa	maly1234	2.52	1.43	18	20	0.83	0.438	0
1011	Yorta Yorta	yort1237	4.26	0.80	15	20	0.83	0.496	0
232	Warnman	wanm1242	5.68	0.87	202	9	0.39	0.840	0
1019	Walmajarri	walm1241	6.97	0.81	226	20	0.87	0.588	0
642	Mangala	mang1383	2.82	1.54	227	14	0.67	0.829	0
519	Nukunu	nugu1241	1.94	1.61	25	22	0.79	0.383	0
1001	Ngandi	ngan1295	4.97	0.85	76	25	0.74	0.305	0
38	Djabugay	dyaa1242	5.65	0.69	188	12	0.63	0.866	0
978	Wotjobaluk	wotj1234	5.14	0.72	58	17	0.89	0.653	0
1031	Ngadjunmaya	ngad1258	4.81	0.95	78	19	0.76	0.562	0
852	Gurindji	guri1247	7.06	0.86	217	20	0.87	0.533	0
965	Oykangand	oyka1239	5.52	0.81	61	24	0.77	0.314	0
1032	Kuku Yalanji	kuku1273	6.30	0.71	207	16	1.00	0.746	0
1026	Gidabal	gida1240	6.04	0.55	184	16	0.80	0.643	0
737	Kukatj	guga1239	5.72	0.37	170	12	0.52	0.755	0

Lex ID	Language	Glottolog	*log* mean	*log* SD	x_{min}	Phonemes	Frac. of inventory	p	G[illegible]
592	Muruwari	muru1266	4.87	1.12	147	17	0.68	0.551	[illegible]
1025	Waalubal	band1358	6.11	0.49	210	15	0.75	0.673	[illegible]
305	Western Wakaya	waga1260	5.30	0.94	26	23	0.88	0.350	[illegible]
838	Gooniyandi	goon1238	4.20	1.37	216	17	0.74	0.594	[illegible]
800	Guwamu	guwa1243	4.59	0.86	19	18	0.86	0.493	[illegible]
101	Butchulla	baty1234	0.82	1.63	85	15	0.62	0.790	[illegible]
563	Ngarla	ngar1296	4.11	1.37	168	18	0.78	0.596	[illegible]
983	Nhangu	yann1237	5.17	1.16	28	31	1.00	0.142	[illegible]
927	Tiwi	tiwi1244	6.63	0.95	202	17	0.85	0.544	[illegible]
1043	Mbabaram	mbab1239	3.68	0.73	10	20	0.74	0.278	[illegible]
105	Bardi	bard1255	4.89	0.85	24	23	0.96	0.280	[illegible]
911	Nyawaygi	nyaw1247	5.06	0.73	67	15	0.83	0.668	[illegible]
920	Yadhaykenu	yadh1237	4.25	1.20	2	25	1.00	0.232	[illegible]
611	Anguthimri	angu1242	2.92	1.22	41	20	0.54	0.398	[illegible]
1016	Tharrkari	dhar1247	3.89	1.03	10	27	0.84	0.108	[illegible]
964	Olkol	ulku1238	5.63	1.00	44	25	0.86	0.208	[illegible]
417	Yindjibarndi	yind1247	4.00	1.23	75	21	0.84	0.377	[illegible]
845	Iwaidja	iwai1244	1.13	1.83	393	13	0.57	0.813	[illegible]
206	Wayilwan	wayi1238	4.56	0.79	170	13	0.62	0.775	[illegible]
901	Guugu Yimidhirr	gugu1255	4.03	0.80	7	21	1.00	0.308	[illegible]
968	Ogh Angkula	ikar1243	4.50	0.78	17	24	1.00	0.223	[illegible]
493	Nyiyaparli	nija1241	3.87	0.87	20	19	0.86	0.347	[illegible]
443	Payungu	bayu1240	1.55	1.85	85	18	0.69	0.540	[illegible]
967	Thaynakwithi	tyan1235	4.77	0.58	45	24	0.75	0.144	[illegible]
929	Wanyjirra	wany1244	3.49	1.35	138	16	0.76	0.570	[illegible]
905	Malkana	malg1242	1.36	1.64	37	17	0.68	0.415	[illegible]
849	Waanyi	wany1247	0.77	1.90	341	12	0.60	0.741	[illegible]
554	Ngarluma	ngar1287	4.91	1.15	182	10	0.43	0.706	[illegible]
1003	Murrinh-patha	murr1259	3.96	0.86	112	9	0.36	0.735	[illegible]
943	Pintupi	pint1250	5.84	1.28	550	19	0.83	0.372	[illegible]
1029	Martuthunira	mart1255	5.07	1.09	13	25	0.96	0.132	[illegible]
914	Yinhawangka	yinh1234	5.75	0.91	221	8	0.31	0.669	[illegible]
1042	Thaayorre	thay1249	6.52	0.83	127	25	0.96	0.130	[illegible]
462	Panyjima	pany1241	5.12	0.81	106	8	0.32	0.633	[illegible]
252	Warlmanpa	warl1255	5.05	1.05	72	20	0.71	0.304	[illegible]
534	Nhanda	nhan1238	3.94	1.18	8	32	0.97	0.031	[illegible]
242	Warlpiri	warl1254	5.69	1.40	602	20	0.83	0.317	[illegible]
363	Yir Yoront	yiry1245	6.09	0.33	212	15	0.58	0.381	[illegible]
787	Jawoyn	djau1244	5.92	0.60	153	21	0.78	0.160	[illegible]
930	Purduna	burd1238	4.73	1.03	19	23	0.88	0.123	[illegible]
835	Gugu Badhun	gugu1253	4.76	0.88	28	17	1.00	0.404	[illegible]
922	Nakara	naka1260	4.91	1.06	88	23	0.88	0.148	[illegible]
972	Ogh Unyjan	kawa1290	4.34	0.78	106	7	0.28	0.711	[illegible]
118	Badimaya	badi1246	4.47	0.95	18	22	0.92	0.121	[illegible]
910	Nungali	nung1291	4.66	0.87	114	14	0.88	0.525	[illegible]
734	Kukatja	kuka1246	6.16	1.11	406	19	0.83	0.228	[illegible]
1021	Watjarri	waja1257	2.50	1.71	113	21	0.81	0.240	[illegible]
790	Jaru	jaru1254	6.16	0.88	121	20	0.87	0.129	[illegible]
13	Dyirbal	dyir1250	5.20	0.73	69	15	0.94	0.265	[illegible]
618	Mirniny	mirn1243	4.14	0.91	10	22	0.88	0.045	[illegible]
1008	Yuwaalaraay	yuwa1242	6.30	0.50	272	14	0.67	0.274	[illegible]
949	Kok Nar	kokn1236	4.71	0.63	96	9	0.43	0.490	[illegible]

Lex ID	Language	Glottolog	*log* mean	*log* SD	x_{min}	Phonemes	Frac. of inventory	p	G[illegible]
1009	Gamilaraay	gami1243	5.07	0.98	20	21	1.00	0.080	[illegible]
853	Lardil	lard1243	6.27	0.83	89	22	0.88	0.049	[illegible]
939	Unggumi	ungg1243	3.28	1.16	70	11	0.37	0.229	[illegible]
89	Bunuba	buna1275	5.02	0.88	40	18	0.75	0.067	[illegible]
977	Warndarrang	wand1263	1.67	1.52	126	6	0.32	0.668	[illegible]
856	Patjtjamalh	wadj1254	5.33	0.75	66	20	0.74	0.043	[illegible]
1040	Ngarinyin	ngar1284	7.59	0.71	691	18	0.67	0.032	[illegible]
1007	Yuwaliyaay	yuwa1243	5.76	1.14	33	21	1.00	0.007	[illegible]

Table S5.5. Exponential distribution.

Lex ID	Language	Glottolog	λ	Phonemes	p	Goodness-of-fit
1002	Yintyingka	ayab1239	0.011	26	0.963	0.08
5	Gangulu	gang1268	0.006	18	0.995	0.08
996	Emmi	amii1238	0.016	31	0.911	0.08
1018	Djapu	djap1238	0.005	26	0.940	0.09
1023	Wubuy	nung1290	0.004	26	0.944	0.09
656	Kuugu Ya'u	kuuk1238	0.004	21	0.972	0.09
952	Ngawun	ngaw1240	0.012	22	0.928	0.09
305	Western Wakaya	waga1260	0.004	26	0.887	0.10
964	Olkol	ulku1238	0.003	29	0.846	0.10
12	Erre	erre1238	0.012	27	0.834	0.10
852	Gurindji	guri1247	0.001	23	0.966	0.10
740	Kugu Nganhcara	wikn1246	0.012	30	0.757	0.10
945	Wiri	biri1256	0.009	18	0.950	0.10
807	Gupapuyngu	gupa1247	0.002	31	0.788	0.10
94	Biri	biri1256	0.004	18	0.956	0.10
1011	Yorta Yorta	yort1237	0.012	24	0.840	0.10
845	Iwaidja	iwai1244	0.002	23	0.904	0.10
495	Nyikina	nyig1240	0.003	20	0.933	0.10
621	Mengerrdji	mang1382	0.011	26	0.783	0.10
540	Ngiyambaa	wang1291	0.014	21	0.872	0.10
1043	Mbabaram	mbab1239	0.026	27	0.700	0.11
979	Mawng	maun1240	0.001	22	0.926	0.11
927	Tiwi	tiwi1244	0.001	20	0.952	0.11
1031	Ngadjunmaya	ngad1258	0.005	25	0.780	0.11
565	Ngarinyman	ngar1235	0.002	23	0.838	0.11
77	Dhangu	dhan1270	0.011	26	0.702	0.11
760	Kartujarra	kart1247	0.004	23	0.793	0.11
1003	Murrinh-patha	murr1259	0.011	25	0.712	0.11
30	Duungidjawu	duun1241	0.009	23	0.765	0.11
1001	Ngandi	ngan1295	0.005	34	0.520	0.11
752	Kija	kitj1240	0.002	24	0.790	0.11
926	Limilngan	nucl1327	0.009	24	0.727	0.11
841	Worrorra	woro1258	0.012	25	0.681	0.12
928	Bakanh	paka1251	0.009	26	0.660	0.12
821	Gumbaynggir	kumb1268	0.015	19	0.825	0.12
968	Ogh Angkula	ikar1243	0.010	24	0.698	0.12
31	Djinang	djin1253	0.002	25	0.807	0.12
800	Guwamu	guwa1243	0.008	21	0.771	0.12
935	Rembarrnga	remb1249	0.005	28	0.606	0.12
631	Yalarnnga	yala1262	0.007	24	0.696	0.12
1024	Ngardily	west2437	0.009	23	0.710	0.12
941	Wambaya	nucl1328	0.002	23	0.777	0.12
790	Jaru	jaru1254	0.002	23	0.808	0.12
642	Mangala	mang1383	0.003	21	0.791	0.12
170	Wik-Ngathan	wikn1245	0.006	28	0.565	0.12
412	Ritharrngu	rita1239	0.004	31	0.517	0.12
851	Bilinarra	bili1250	0.002	23	0.753	0.12
1012	Warrgamay	warr1255	0.004	19	0.801	0.12
62	Dhay'yi	dhal1246	0.004	26	0.598	0.12
737	Kukatj	guga1239	0.005	23	0.659	0.12
838	Gooniyandi	goon1238	0.002	23	0.709	0.12

Lex ID	Language	Glottolog	λ	Phonemes	p	Goodness-of-fit
697	Kurrama	kurr1243	0.005	27	0.547	0.12
105	Bardi	bard1255	0.006	24	0.619	0.13
848	Wagiman	wage1238	0.003	27	0.558	0.13
606	Mudburra	mudb1240	0.004	23	0.641	0.13
853	Lardil	lard1243	0.002	25	0.671	0.13
650	Linngithigh	leni1238	0.011	29	0.436	0.13
546	Yanyuwa	yany1243	0.002	23	0.714	0.13
1009	Gamilaraay	gami1243	0.005	21	0.629	0.13
99	Bidyara	bidy1243	0.004	19	0.690	0.13
767	Karajarri	kara1476	0.002	21	0.724	0.13
778	Jiwarli	djiw1241	0.004	26	0.483	0.13
918	Atampaya	atam1239	0.008	25	0.471	0.14
771	Kalkatungu	kalk1246	0.003	26	0.487	0.14
857	Amurdak	amar1271	0.010	24	0.467	0.14
978	Wotjobaluk	wotj1234	0.006	19	0.642	0.14
200	Western Arrernte	west2441	0.012	25	0.428	0.14
965	Oykangand	oyka1239	0.004	31	0.312	0.14
377	Umpila	umpi1239	0.005	22	0.547	0.14
917	Nyamal	nyam1271	0.004	24	0.478	0.14
63	Dharumbal	dhar1248	0.007	21	0.555	0.14
856	Patjtjamalh	wadj1254	0.005	27	0.392	0.14
288	Wangkatja	pini1245	0.002	23	0.565	0.14
443	Payungu	bayu1240	0.006	26	0.398	0.14
427	Yidiny	yidi1250	0.002	19	0.670	0.14
563	Ngarla	ngar1296	0.003	23	0.523	0.14
921	Gurr-Goni	gura1252	0.002	27	0.427	0.14
957	Ngaanyatjarra	ngaa1240	0.002	23	0.533	0.14
919	Angkamuthi	angg1238	0.006	26	0.367	0.14
89	Bunuba	buna1275	0.006	24	0.411	0.14
13	Dyirbal	dyir1250	0.005	16	0.684	0.15
1019	Walmajarri	walm1241	0.001	23	0.714	0.15
762	Kariyarra	kari1304	0.010	24	0.398	0.15
1016	Tharrkari	dhar1247	0.013	32	0.206	0.15
911	Nyawaygi	nyaw1247	0.006	18	0.591	0.15
117	Wirangu	wira1265	0.008	23	0.422	0.15
118	Badimaya	badi1246	0.008	24	0.375	0.15
417	Yindjibarndi	yind1247	0.005	25	0.359	0.15
982	Nhirrpi	nhir1234	0.017	30	0.226	0.15
787	Jawoyn	djau1244	0.003	27	0.327	0.15
920	Yadhaykenu	yadh1237	0.008	25	0.342	0.15
985	Yaygir	yayg1236	0.004	20	0.510	0.15
228	Warriyangga	wari1262	0.012	26	0.298	0.15
905	Malkana	malg1242	0.014	25	0.320	0.15
939	Unggumi	ungg1243	0.014	30	0.212	0.15
929	Wanyjirra	wany1244	0.004	21	0.456	0.15
493	Nyiyaparli	nija1241	0.014	22	0.380	0.15
972	Ogh Unyjan	kawa1290	0.011	25	0.312	0.15
592	Muruwari	muru1266	0.004	25	0.330	0.15
204	Wemba Wemba	wemb1241	0.003	22	0.422	0.15
835	Gugu Badhun	gugu1253	0.007	17	0.566	0.15
934	Urningangg	urni1239	0.011	27	0.245	0.15
946	Kurtjar	gurd1238	0.005	28	0.257	0.15

Lex ID	Language	Glottolog	λ	Phonemes	p	Goodness-of-fit
1032	Kuku Yalanji	kuku1273	0.002	16	0.688	0.15
1008	Yuwaalaraay	yuwa1242	0.002	21	0.466	0.15
930	Purduna	burd1238	0.006	26	0.270	0.15
242	Warlpiri	warl1254	0.001	24	0.684	0.15
977	Warndarrang	wand1263	0.010	19	0.458	0.16
1029	Martuthunira	mart1255	0.004	26	0.275	0.16
265	Wardaman	ward1246	0.002	22	0.431	0.16
462	Panyjima	pany1241	0.008	25	0.268	0.16
232	Warnman	wanm1242	0.003	23	0.347	0.16
949	Kok Nar	kokn1236	0.011	21	0.374	0.16
967	Thaynakwithi	tyan1235	0.009	32	0.142	0.16
113	Yulparija	yulp1239	0.002	23	0.420	0.16
618	Mirniny	mirn1243	0.012	25	0.245	0.16
1030	Putijarra	pudi1238	0.003	25	0.256	0.16
475	Southern Paakintyi	darl1243	0.003	26	0.238	0.16
734	Kukatja	kuka1246	0.001	23	0.590	0.16
269	Wangkumara	wong1246	0.006	30	0.139	0.16
85	Burarra	bura1267	0.002	26	0.326	0.17
554	Ngarluma	ngar1287	0.004	23	0.275	0.17
400	Thalanyji	dhal1245	0.006	26	0.199	0.17
162	Adnyamathanha	adny1235	0.003	29	0.167	0.17
847	Ngalakgan	ngal1293	0.005	27	0.178	0.17
237	Warluwarra	warl1256	0.005	33	0.083	0.17
1026	Gidabal	gida1240	0.003	20	0.341	0.17
38	Djabugay	dyaa1242	0.003	19	0.362	0.17
901	Guugu Yimidhirr	gugu1255	0.015	21	0.263	0.17
206	Wayilwan	wayi1238	0.006	21	0.266	0.17
363	Yir Yoront	yiry1245	0.003	26	0.173	0.17
1007	Yuwaliyaay	yuwa1243	0.002	21	0.329	0.17
645	Malyangapa	maly1234	0.017	24	0.165	0.17
849	Waanyi	wany1247	0.002	20	0.349	0.18
865	Diyari	dier1241	0.015	25	0.140	0.18
866	Thirarri	dira1238	0.015	25	0.142	0.18
922	Nakara	naka1260	0.003	26	0.156	0.18
433	Pitta Pitta	pitt1247	0.006	25	0.149	0.18
598	Yandruwandha	yand1253	0.005	30	0.080	0.18
1006	Central Arrernte	mpar1238	0.004	25	0.144	0.18
1021	Watjarri	waja1257	0.004	26	0.127	0.18
923	Matngele	madn1237	0.006	25	0.130	0.18
966	Wik Mungkan	wikm1247	0.001	26	0.328	0.18
1042	Thaayorre	thay1249	0.001	26	0.240	0.18
1025	Waalubal	band1358	0.003	20	0.236	0.19
914	Yinhawangka	yinh1234	0.004	26	0.115	0.19
863	Marra	mara1385	0.004	20	0.212	0.19
519	Nukunu	nugu1241	0.014	28	0.056	0.19
925	Larrakia	lara1258	0.007	29	0.057	0.19
91	Bularnu	bula1255	0.007	30	0.045	0.19
611	Anguthimri	angu1242	0.017	37	0.012	0.20
958	Margany	marg1253	0.016	30	0.029	0.20
1040	Ngarinyin	ngar1284	0.001	27	0.329	0.20
962	Gunya	guny1241	0.009	31	0.027	0.21
534	Nhanda	nhan1238	0.010	33	0.018	0.21

**Lex ID**	**Language**	**Glottolog**	λ	**Phonemes**	p	**Goodness-of-fit**
983	Nhangu	yann1237	0.003	31	0.027	0.21
81	Dalabon	ngal1292	0.002	29	0.039	0.22
744	Koko Bera	gugu1254	0.003	21	0.050	0.23
507	Nyangumarta	nyan1301	0.002	21	0.054	0.24
943	Pintupi	pint1250	0.001	23	0.121	0.24
915	Alawa	alaw1244	0.002	21	0.067	0.25
940	Yawijibaya	yawi1239	0.016	23	0.012	0.26
910	Nungali	nung1291	0.005	16	0.055	0.26
101	Butchulla	baty1234	0.009	24	0.005	0.26
252	Warlmanpa	warl1255	0.004	28	0.002	0.27
620	Miriwoong	miri1266	0.001	20	0.036	0.27

Table S5.6. Exponential distribution with x_{min}.

Lex ID	Language	Glottolog	λ	x_{min}	Phonemes	Frac. of inventory	p	Goodness-of-fi
1002	Yintyingka	ayab1239	0.011	11	24	0.92	0.974	0.0
915	Alawa	alaw1244	0.002	253	20	0.95	0.996	0.0
1023	Wubuy	nung1290	0.003	12	25	0.96	0.962	0.0
740	Kugu Nganhcara	wikn1246	0.011	11	26	0.87	0.914	0.0
966	Wik Mungkan	wikm1247	0.001	575	17	0.65	0.991	0.0
427	Yidiny	yidi1250	0.003	163	18	0.95	0.995	0.0
5	Gangulu	gang1268	0.006	9	18	1.00	0.992	0.0
996	Emmi	amii1238	0.016	2	31	1.00	0.827	0.0
265	Wardaman	ward1246	0.003	224	18	0.82	0.981	0.0
1018	Djapu	djap1238	0.005	25	26	1.00	0.878	0.0
925	Larrakia	lara1258	0.005	36	19	0.66	0.927	0.0
656	Kuugu Ya'u	kuuk1238	0.004	73	20	0.95	0.949	0.0
565	Ngarinyman	ngar1235	0.003	115	19	0.83	0.956	0.0
952	Ngawun	ngaw1240	0.011	10	19	0.86	0.919	0.0
412	Ritharrngu	rita1239	0.003	41	28	0.90	0.752	0.0
697	Kurrama	kurr1243	0.005	7	25	0.93	0.826	0.0
935	Rembarrnga	remb1249	0.006	64	25	0.89	0.824	0.0
377	Umpila	umpi1239	0.005	37	21	0.95	0.900	0.0
495	Nyikina	nyig1240	0.002	67	19	0.95	0.932	0.1
928	Bakanh	paka1251	0.011	52	19	0.73	0.837	0.1
621	Mengerrdji	mang1382	0.013	65	15	0.58	0.939	0.1
305	Western Wakaya	waga1260	0.004	1	26	1.00	0.788	0.1
964	Olkol	ulku1238	0.003	1	29	1.00	0.714	0.1
921	Gurr-Goni	gura1252	0.003	173	22	0.81	0.850	0.1
744	Koko Bera	gugu1254	0.005	313	11	0.52	0.980	0.1
852	Gurindji	guri1247	0.001	45	22	0.96	0.931	0.1
12	Erre	erre1238	0.012	1	27	1.00	0.702	0.1
926	Limilngan	nucl1327	0.009	3	23	0.96	0.786	0.1
540	Ngiyambaa	wang1291	0.013	27	16	0.76	0.868	0.1
939	Unggumi	ungg1243	0.012	10	23	0.77	0.726	0.1
1043	Mbabaram	mbab1239	0.026	8	23	0.85	0.647	0.1
945	Wiri	biri1256	0.009	6	18	1.00	0.921	0.1
807	Gupapuyngu	gupa1247	0.002	49	31	1.00	0.589	0.1
94	Biri	biri1256	0.004	12	18	1.00	0.916	0.1
934	Urningangg	urni1239	0.010	17	22	0.81	0.752	0.1
1011	Yorta Yorta	yort1237	0.012	14	21	0.88	0.759	0.1
1012	Warrgamay	warr1255	0.004	105	15	0.79	0.931	0.1
965	Oykangand	oyka1239	0.004	58	25	0.81	0.677	0.1
105	Bardi	bard1255	0.006	78	16	0.67	0.889	0.1
845	Iwaidja	iwai1244	0.002	24	23	1.00	0.800	0.1
63	Dharumbal	dhar1248	0.006	37	15	0.71	0.899	0.1
841	Worrorra	woro1258	0.013	22	22	0.88	0.722	0.1
863	Marra	mara1385	0.005	67	19	0.95	0.878	0.1
1001	Ngandi	ngan1295	0.005	3	33	0.97	0.439	0.1
170	Wik-Ngathan	wikn1245	0.007	231	13	0.46	0.926	0.1
767	Karajarri	kara1476	0.002	111	19	0.90	0.829	0.1
204	Wemba Wemba	wemb1241	0.004	97	20	0.91	0.786	0.1
611	Anguthimri	angu1242	0.014	7	28	0.76	0.429	0.1
821	Gumbaynggir	kumb1268	0.016	62	15	0.79	0.851	0.1
30	Duungidjawu	duun1241	0.010	43	19	0.83	0.797	0.1
979	Mawng	maun1240	0.001	86	22	1.00	0.807	0.1

Lex ID	Language	Glottolog	λ	x_{min}	Phonemes	Frac. of inventory	p	Goodness-of-fi
927	Tiwi	tiwi1244	0.001	89	20	1.00	0.869	0.1
81	Dalabon	ngal1292	0.002	580	18	0.62	0.850	0.1
1031	Ngadjunmaya	ngad1258	0.005	2	25	1.00	0.628	0.1
968	Ogh Angkula	ikar1243	0.010	30	23	0.96	0.621	0.1
851	Bilinarra	bili1250	0.002	162	19	0.83	0.802	0.1
857	Amurdak	amar1271	0.009	3	22	0.92	0.655	0.1
77	Dhangu	dhan1270	0.011	7	26	1.00	0.516	0.1
752	Kija	kitj1240	0.003	269	16	0.67	0.860	0.1
760	Kartujarra	kart1247	0.004	4	23	1.00	0.635	0.1
1003	Murrinh-patha	murr1259	0.011	7	25	1.00	0.533	0.1
943	Pintupi	pint1250	0.001	424	20	0.87	0.826	0.1
848	Wagiman	wage1238	0.004	130	22	0.81	0.632	0.1
917	Nyamal	nyam1271	0.004	2	22	0.92	0.626	0.1
978	Wotjobaluk	wotj1234	0.006	83	16	0.84	0.809	0.1
232	Warnman	wanm1242	0.003	202	9	0.39	0.949	0.1
946	Kurtjar	gurd1238	0.008	164	18	0.64	0.589	0.1
38	Djabugay	dyaa1242	0.004	188	12	0.63	0.902	0.1
650	Linngithigh	leni1238	0.011	2	28	0.97	0.342	0.1
31	Djinang	djin1253	0.002	24	25	1.00	0.569	0.1
620	Miriwoong	miri1266	0.002	538	14	0.70	0.906	0.1
800	Guwamu	guwa1243	0.008	3	21	1.00	0.622	0.1
941	Wambaya	nucl1328	0.002	5	22	0.96	0.610	0.1
631	Yalarnnga	yala1262	0.007	6	24	1.00	0.507	0.1
1024	Ngardily	west2437	0.009	3	23	1.00	0.527	0.1
790	Jaru	jaru1254	0.002	30	23	1.00	0.590	0.1
847	Ngalakgan	ngal1293	0.007	119	20	0.74	0.575	0.1
642	Mangala	mang1383	0.003	13	21	1.00	0.624	0.1
911	Nyawaygi	nyaw1247	0.006	67	15	0.83	0.807	0.1
923	Matngele	madn1237	0.007	109	18	0.72	0.641	0.1
62	Dhay'yi	dhal1246	0.003	228	10	0.38	0.662	0.1
737	Kukatj	guga1239	0.005	38	23	1.00	0.466	0.1
838	Gooniyandi	goon1238	0.002	15	23	1.00	0.492	0.1
967	Thaynakwithi	tyan1235	0.012	67	23	0.72	0.373	0.1
901	Guugu Yimidhirr	gugu1255	0.018	26	19	0.90	0.521	0.1
771	Kalkatungu	kalk1246	0.003	46	24	0.92	0.383	0.1
962	Gunya	guny1241	0.007	11	25	0.81	0.314	0.1
606	Mudburra	mudb1240	0.004	2	23	1.00	0.436	0.1
475	Southern Paakintyi	darl1243	0.003	175	13	0.50	0.676	0.1
853	Lardil	lard1243	0.002	50	25	1.00	0.398	0.1
972	Ogh Unyjan	kawa1290	0.013	31	22	0.88	0.310	0.1
546	Yanyuwa	yany1243	0.002	27	23	1.00	0.431	0.1
856	Patjtjamalh	wadj1254	0.004	2	25	0.93	0.260	0.1
1009	Gamilaraay	gami1243	0.005	20	21	1.00	0.414	0.1
99	Bidyara	bidy1243	0.004	7	19	1.00	0.521	0.1
778	Jiwarli	djiw1241	0.004	11	26	1.00	0.247	0.1
592	Muruwari	muru1266	0.003	63	22	0.88	0.318	0.1
1032	Kuku Yalanji	kuku1273	0.002	368	13	0.81	0.740	0.1
918	Atampaya	atam1239	0.008	1	25	1.00	0.245	0.1
237	Warluwarra	warl1256	0.004	22	26	0.79	0.166	0.1
1026	Gidabal	gida1240	0.004	283	14	0.70	0.604	0.1
200	Western Arrernte	west2441	0.012	8	25	1.00	0.206	0.1
206	Wayilwan	wayi1238	0.008	170	13	0.62	0.700	0.1

Lex ID	Language	Glottolog	λ	x_{min}	Phonemes	Frac. of inventory	p	Goodness-of-fi
288	Wangkatja	pini1245	0.002	48	23	1.00	0.301	0.1
940	Yawijibaya	yawi1239	0.013	9	18	0.78	0.391	0.1
958	Margany	marg1253	0.014	7	24	0.80	0.175	0.1
443	Payungu	bayu1240	0.006	5	26	1.00	0.176	0.1
949	Kok Nar	kokn1236	0.012	92	10	0.48	0.682	0.1
563	Ngarla	ngar1296	0.003	2	23	1.00	0.273	0.1
519	Nukunu	nugu1241	0.009	53	9	0.32	0.676	0.1
1019	Walmajarri	walm1241	0.001	226	20	0.87	0.392	0.1
957	Ngaanyatjarra	ngaa1240	0.002	57	23	1.00	0.246	0.1
910	Nungali	nung1291	0.007	114	14	0.88	0.637	0.1
919	Angkamuthi	angg1238	0.006	5	26	1.00	0.152	0.1
89	Bunuba	buna1275	0.006	7	24	1.00	0.196	0.1
1025	Waalubal	band1358	0.004	287	14	0.70	0.536	0.1
13	Dyirbal	dyir1250	0.005	59	16	1.00	0.498	0.1
985	Yaygir	yayg1236	0.005	107	17	0.85	0.364	0.1
762	Kariyarra	kari1304	0.010	2	24	1.00	0.174	0.1
252	Warlmanpa	warl1255	0.003	6	22	0.79	0.216	0.1
1016	Tharrkari	dhar1247	0.013	2	32	1.00	0.052	0.1
117	Wirangu	wira1265	0.008	3	23	1.00	0.190	0.1
598	Yandruwandha	yand1253	0.004	89	14	0.47	0.400	0.1
118	Badimaya	badi1246	0.008	3	24	1.00	0.169	0.1
417	Yindjibarndi	yind1247	0.005	17	25	1.00	0.152	0.1
982	Nhirrpi	nhir1234	0.017	1	30	1.00	0.060	0.1
787	Jawoyn	djau1244	0.003	63	27	1.00	0.109	0.1
920	Yadhaykenu	yadh1237	0.008	2	25	1.00	0.138	0.1
85	Burarra	bura1267	0.002	284	21	0.81	0.190	0.1
228	Warriyangga	wari1262	0.012	1	26	1.00	0.101	0.1
905	Malkana	malg1242	0.014	2	25	1.00	0.124	0.1
400	Thalanyji	dhal1245	0.004	161	7	0.27	0.593	0.1
929	Wanyjirra	wany1244	0.004	38	21	1.00	0.222	0.1
493	Nyiyaparli	nija1241	0.014	1	22	1.00	0.171	0.1
462	Panyjima	pany1241	0.005	106	8	0.32	0.594	0.1
113	Yulparija	yulp1239	0.002	151	20	0.87	0.224	0.1
835	Gugu Badhun	gugu1253	0.007	28	17	1.00	0.360	0.1
1008	Yuwaalaraay	yuwa1242	0.003	450	11	0.52	0.533	0.1
930	Purduna	burd1238	0.006	12	26	1.00	0.082	0.1
242	Warlpiri	warl1254	0.001	63	24	1.00	0.317	0.1
849	Waanyi	wany1247	0.002	211	15	0.75	0.395	0.1
433	Pitta Pitta	pitt1247	0.004	150	7	0.28	0.603	0.1
977	Warndarrang	wand1263	0.010	35	19	1.00	0.233	0.1
1029	Martuthunira	mart1255	0.004	8	26	1.00	0.084	0.1
1042	Thaayorre	thay1249	0.002	209	24	0.92	0.086	0.1
618	Mirniny	mirn1243	0.012	1	25	1.00	0.066	0.1
645	Malyangapa	maly1234	0.011	57	7	0.29	0.559	0.1
1030	Putijarra	pudi1238	0.003	1	25	1.00	0.062	0.1
734	Kukatja	kuka1246	0.001	35	23	1.00	0.222	0.1
269	Wangkumara	wong1246	0.006	4	30	1.00	0.023	0.1
554	Ngarluma	ngar1287	0.004	30	23	1.00	0.088	0.1
162	Adnyamathanha	adny1235	0.003	63	29	1.00	0.023	0.1
507	Nyangumarta	nyan1301	0.002	307	9	0.43	0.542	0.1
363	Yir Yoront	yiry1245	0.003	41	25	0.96	0.028	0.1
1007	Yuwaliyaay	yuwa1243	0.002	33	21	1.00	0.094	0.1

Lex ID	Language	Glottolog	λ	x_{min}	Phonemes	Frac. of inventory	p	Goodness-of-fi
865	Diyari	dier1241	0.015	13	23	0.92	0.040	0.1
866	Thirarri	dira1238	0.014	13	23	0.92	0.038	0.1
914	Yinhawangka	yinh1234	0.002	221	8	0.31	0.408	0.1
922	Nakara	naka1260	0.003	15	26	1.00	0.021	0.1
534	Nhanda	nhan1238	0.004	106	6	0.18	0.525	0.1
1006	Central Arrernte	mpar1238	0.004	27	25	1.00	0.024	0.1
1021	Watjarri	waja1257	0.004	5	26	1.00	0.018	0.1
101	Butchulla	baty1234	0.011	85	15	0.62	0.140	0.1
983	Nhangu	yann1237	0.003	130	19	0.61	0.023	0.1
91	Bularnu	bula1255	0.007	2	30	1.00	0.003	0.1
1040	Ngarinyin	ngar1284	0.001	2	27	1.00	0.044	0.2

Table S5.7. Poisson distribution.

Lex ID	Language variety	Glottolog	λ	Phonemes	p	Goodness-of-fit
972	Ogh Unyjan	kawa1290	95.36	25	0	0.43
363	Yir Yoront	yiry1245	323.77	26	0	0.46
206	Wayilwan	wayi1238	204.33	21	0	0.46
967	Thaynakwithi	tyan1235	114.44	32	0	0.46
787	Jawoyn	djau1244	385.67	27	0	0.47
928	Bakanh	paka1251	110.96	26	0	0.47
81	Dalabon	ngal1292	724.45	29	0	0.47
940	Yawijibaya	yawi1239	66.74	23	0	0.47
934	Urningangg	urni1239	92.19	27	0	0.47
821	Gumbaynggir	kumb1268	108.58	19	0	0.48
1007	Yuwaliyaay	yuwa1243	520.43	21	0	0.48
1008	Yuwaalaraay	yuwa1242	471.29	21	0	0.48
1009	Gamilaraay	gami1243	236.57	21	0	0.48
946	Kurtjar	gurd1238	220.93	28	0	0.48
101	Butchulla	baty1234	113.88	24	0	0.49
1026	Gidabal	gida1240	424.50	20	0	0.49
621	Mengerrdji	mang1382	91.27	26	0	0.50
1025	Waalubal	band1358	425.55	20	0	0.50
949	Kok Nar	kokn1236	107.24	21	0	0.50
968	Ogh Angkula	ikar1243	121.21	24	0	0.50
170	Wik-Ngathan	wikn1245	220.36	28	0	0.50
939	Unggumi	ungg1243	74.57	30	0	0.50
1043	Mbabaram	mbab1239	40.41	27	0	0.51
650	Linngithigh	leni1238	90.52	29	0	0.51
540	Ngiyambaa	wang1291	82.86	21	0	0.51
848	Wagiman	wage1238	334.41	27	0	0.51
847	Ngalakgan	ngal1293	208.41	27	0	0.51
923	Matngele	madn1237	183.48	25	0	0.51
744	Koko Bera	gugu1254	358.52	21	0	0.52
856	Patjtjamalh	wadj1254	215.96	27	0	0.52
1040	Ngarinyin	ngar1284	1852.33	27	0	0.52
901	Guugu Yimidhirr	gugu1255	75.29	21	0	0.52
38	Djabugay	dyaa1242	314.11	19	0	0.52
1003	Murrinh-patha	murr1259	100.48	25	0	0.52
853	Lardil	lard1243	644.88	25	0	0.52
790	Jaru	jaru1254	637.00	23	0	0.52
737	Kukatj	guga1239	221.43	23	0	0.52
800	Guwamu	guwa1243	125.10	21	0	0.52
89	Bunuba	buna1275	178.71	24	0	0.53
996	Emmi	amii1238	64.35	31	0	0.53
910	Nungali	nung1291	226.69	16	0	0.53
1002	Yintyingka	ayab1239	98.00	26	0	0.53
958	Margany	marg1253	64.57	30	0	0.53
30	Duungidjawu	duun1241	124.96	23	0	0.53
985	Yaygir	yayg1236	287.05	20	0	0.54
1042	Thaayorre	thay1249	891.58	26	0	0.54
978	Wotjobaluk	wotj1234	207.32	19	0	0.54
493	Nyiyaparli	nija1241	69.91	22	0	0.54
965	Oykangand	oyka1239	272.42	31	0	0.54
1011	Yorta Yorta	yort1237	84.17	24	0	0.54
979	Mawng	maun1240	876.45	22	0	0.55

Lex ID	Language variety	Glottolog	λ	Phonemes	p	Goodness-of-fit
611	Anguthimri	angu1242	60.22	37	0	0.55
841	Worrorra	woro1258	86.44	25	0	0.55
94	Biri	biri1256	234.89	18	0	0.55
857	Amurdak	amar1271	104.42	24	0	0.55
1001	Ngandi	ngan1295	188.47	34	0	0.55
5	Gangulu	gang1268	166.50	18	0	0.56
265	Wardaman	ward1246	522.41	22	0	0.56
427	Yidiny	yidi1250	540.16	19	0	0.56
740	Kugu Nganhcara	wikn1246	88.50	30	0	0.56
620	Miriwoong	miri1266	833.05	20	0	0.56
12	Erre	erre1238	86.59	27	0	0.56
63	Dharumbal	dhar1248	143.76	21	0	0.57
252	Warlmanpa	warl1255	237.64	28	0	0.57
642	Mangala	mang1383	375.52	21	0	0.57
1012	Warrgamay	warr1255	284.89	19	0	0.57
952	Ngawun	ngaw1240	84.55	22	0	0.57
982	Nhirrpi	nhir1234	59.30	30	0	0.58
305	Western Wakaya	waga1260	266.23	26	0	0.58
1032	Kuku Yalanji	kuku1273	754.94	16	0	0.58
977	Warndarrang	wand1263	137.26	19	0	0.58
911	Nyawaygi	nyaw1247	190.11	18	0	0.58
983	Nhangu	yann1237	348.19	31	0	0.58
752	Kija	kitj1240	455.96	24	0	0.58
1031	Ngadjunmaya	ngad1258	202.76	25	0	0.58
926	Limilngan	nucl1327	111.21	24	0	0.58
105	Bardi	bard1255	185.58	24	0	0.58
925	Larrakia	lara1258	153.45	29	0	0.58
852	Gurindji	guri1247	1478.52	23	0	0.59
377	Umpila	umpi1239	223.09	22	0	0.59
1016	Tharrkari	dhar1247	75.97	32	0	0.59
443	Payungu	bayu1240	183.73	26	0	0.59
228	Warriyangga	wari1262	80.85	26	0	0.59
945	Wiri	biri1256	122.44	18	0	0.59
905	Malkana	malg1242	73.84	25	0	0.60
204	Wemba Wemba	wemb1241	348.05	22	0	0.60
77	Dhangu	dhan1270	95.50	26	0	0.60
495	Nyikina	nyig1240	467.05	20	0	0.60
927	Tiwi	tiwi1244	1096.60	20	0	0.60
606	Mudburra	mudb1240	242.74	23	0	0.60
922	Nakara	naka1260	320.50	26	0	0.61
935	Rembarrnga	remb1249	221.36	28	0	0.61
237	Warluwarra	warl1256	215.55	33	0	0.61
915	Alawa	alaw1244	817.81	21	0	0.61
966	Wik Mungkan	wikm1247	1039.35	26	0	0.61
851	Bilinarra	bili1250	503.00	23	0	0.61
546	Yanyuwa	yany1243	675.48	23	0	0.61
845	Iwaidja	iwai1244	525.52	23	0	0.61
534	Nhanda	nhan1238	103.94	33	0	0.61
200	Western Arrernte	west2441	91.20	25	0	0.61
941	Wambaya	nucl1328	508.61	23	0	0.61
412	Ritharrngu	rita1239	310.35	31	0	0.61
118	Badimaya	badi1246	132.83	24	0	0.62

Lex ID	Language variety	Glottolog	λ	Phonemes	p	Goodness-of-fit
964	Olkol	ulku1238	373.90	29	0	0.62
920	Yadhaykenu	yadh1237	129.56	25	0	0.62
921	Gurr-Goni	gura1252	482.04	27	0	0.62
929	Wanyjirra	wany1244	271.57	21	0	0.62
417	Yindjibarndi	yind1247	203.76	25	0	0.62
598	Yandruwandha	yand1253	185.23	30	0	0.62
697	Kurrama	kurr1243	197.96	27	0	0.63
645	Malyangapa	maly1234	60.75	24	0	0.63
838	Gooniyandi	goon1238	453.39	23	0	0.63
1029	Martuthunira	mart1255	263.38	26	0	0.63
863	Marra	mara1385	265.40	20	0	0.64
917	Nyamal	nyam1271	254.79	24	0	0.64
592	Muruwari	muru1266	321.00	25	0	0.64
849	Waanyi	wany1247	487.40	20	0	0.64
962	Gunya	guny1241	119.45	31	0	0.64
1018	Djapu	djap1238	225.23	26	0	0.64
771	Kalkatungu	kalk1246	373.27	26	0	0.64
618	Mirniny	mirn1243	86.88	25	0	0.64
565	Ngarinyman	ngar1235	410.22	23	0	0.64
807	Gupapuyngu	gupa1247	520.00	31	0	0.64
1024	Ngardily	west2437	119.57	23	0	0.65
656	Kuugu Ya'u	kuuk1238	305.05	21	0	0.65
117	Wirangu	wira1265	125.87	23	0	0.65
1023	Wubuy	nung1290	294.08	26	0	0.65
760	Kartujarra	kart1247	238.70	23	0	0.65
919	Angkamuthi	angg1238	180.88	26	0	0.65
1019	Walmajarri	walm1241	1323.57	23	0	0.65
232	Warnman	wanm1242	295.09	23	0	0.65
943	Pintupi	pint1250	1472.17	23	0	0.65
631	Yalarnnga	yala1262	159.67	24	0	0.65
85	Burarra	bura1267	741.31	26	0	0.65
930	Purduna	burd1238	174.46	26	0	0.65
475	Southern Paakintyi	darl1243	326.04	26	0	0.65
62	Dhay'yi	dhal1246	296.73	26	0	0.65
778	Jiwarli	djiw1241	289.15	26	0	0.65
762	Kariyarra	kari1304	105.88	24	0	0.66
1030	Putijarra	pudi1238	297.12	25	0	0.66
99	Bidyara	bidy1243	246.32	19	0	0.66
767	Karajarri	kara1476	599.24	21	0	0.66
865	Diyari	dier1241	75.92	25	0	0.67
866	Thirarri	dira1238	75.96	25	0	0.67
91	Bularnu	bula1255	138.17	30	0	0.67
507	Nyangumarta	nyan1301	459.95	21	0	0.67
957	Ngaanyatjarra	ngaa1240	579.65	23	0	0.67
918	Atampaya	atam1239	124.20	25	0	0.67
31	Djinang	djin1253	673.56	25	0	0.68
13	Dyirbal	dyir1250	242.69	16	0	0.68
400	Thalanyji	dhal1245	175.85	26	0	0.69
835	Gugu Badhun	gugu1253	175.12	17	0	0.69
113	Yulparija	yulp1239	580.78	23	0	0.70
554	Ngarluma	ngar1287	293.52	23	0	0.70
288	Wangkatja	pini1245	608.83	23	0	0.70

Lex ID	Language variety	Glottolog	λ	Phonemes	p	Goodness-of-fit
734	Kukatja	kuka1246	1175.09	23	0	0.70
269	Wangkumara	wong1246	172.03	30	0	0.70
462	Panyjima	pany1241	129.20	25	0	0.70
519	Nukunu	nugu1241	71.93	28	0	0.70
563	Ngarla	ngar1296	392.61	23	0	0.71
242	Warlpiri	warl1254	1704.46	24	0	0.71
1006	Central Arrernte	mpar1238	286.56	25	0	0.72
162	Adnyamathanha	adny1235	412.72	29	0	0.72
433	Pitta Pitta	pitt1247	173.52	25	0	0.72
914	Yinhawangka	yinh1234	284.54	26	0	0.73
1021	Watjarri	waja1257	279.73	26	0	0.75

Table S5.8. Poisson distribution with x_{min}.

Lex ID	Language	Glottolog	λ	x_{min}	Phonemes	Frac. of inventory	p	Goodness-of-fit
1002	Yintyingka	ayab1239	0.011	11	24	0.92	0.97	0.07
915	Alawa	alaw1244	0.002	253	20	0.95	1.00	0.08
1023	Wubuy	nung1290	0.003	12	25	0.96	0.96	0.08
740	Kugu Nganhcara	wikn1246	0.011	11	26	0.87	0.91	0.08
966	Wik Mungkan	wikm1247	0.001	575	17	0.65	0.99	0.08
427	Yidiny	yidi1250	0.003	163	18	0.95	0.99	0.08
5	Gangulu	gang1268	0.006	9	18	1.00	0.99	0.08
996	Emmi	amii1238	0.016	2	31	1.00	0.83	0.08
265	Wardaman	ward1246	0.003	224	18	0.82	0.98	0.09
1018	Djapu	djap1238	0.005	25	26	1.00	0.88	0.09
925	Larrakia	lara1258	0.005	36	19	0.66	0.93	0.09
656	Kuugu Ya'u	kuuk1238	0.004	73	20	0.95	0.95	0.09
565	Ngarinyman	ngar1235	0.003	115	19	0.83	0.96	0.09
952	Ngawun	ngaw1240	0.011	10	19	0.86	0.92	0.09
412	Ritharrngu	rita1239	0.003	41	28	0.90	0.75	0.09
697	Kurrama	kurr1243	0.005	7	25	0.93	0.83	0.09
935	Rembarrnga	remb1249	0.006	64	25	0.89	0.82	0.09
377	Umpila	umpi1239	0.005	37	21	0.95	0.90	0.09
495	Nyikina	nyig1240	0.002	67	19	0.95	0.93	0.10
928	Bakanh	paka1251	0.011	52	19	0.73	0.84	0.10
621	Mengerrdji	mang1382	0.013	65	15	0.58	0.94	0.10
305	Western Wakaya	waga1260	0.004	1	26	1.00	0.79	0.10
964	Olkol	ulku1238	0.003	1	29	1.00	0.71	0.10
921	Gurr-Goni	gura1252	0.003	173	22	0.81	0.85	0.10
744	Koko Bera	gugu1254	0.005	313	11	0.52	0.98	0.10
852	Gurindji	guri1247	0.001	45	22	0.96	0.93	0.10
12	Erre	erre1238	0.012	1	27	1.00	0.70	0.10
926	Limilngan	nucl1327	0.009	3	23	0.96	0.79	0.10
540	Ngiyambaa	wang1291	0.013	27	16	0.76	0.87	0.10
939	Unggumi	ungg1243	0.012	10	23	0.77	0.73	0.10
1043	Mbabaram	mbab1239	0.026	8	23	0.85	0.65	0.10
945	Wiri	biri1256	0.009	6	18	1.00	0.92	0.10
807	Gupapuyngu	gupa1247	0.002	49	31	1.00	0.59	0.10
94	Biri	biri1256	0.004	12	18	1.00	0.92	0.10
934	Urningangg	urni1239	0.010	17	22	0.81	0.75	0.10
1011	Yorta Yorta	yort1237	0.012	14	21	0.88	0.76	0.10
1012	Warrgamay	warr1255	0.004	105	15	0.79	0.93	0.10
965	Oykangand	oyka1239	0.004	58	25	0.81	0.68	0.10
105	Bardi	bard1255	0.006	78	16	0.67	0.89	0.10
845	Iwaidja	iwai1244	0.002	24	23	1.00	0.80	0.10
63	Dharumbal	dhar1248	0.006	37	15	0.71	0.90	0.10
841	Worrorra	woro1258	0.013	22	22	0.88	0.72	0.10
863	Marra	mara1385	0.005	67	19	0.95	0.88	0.10
1001	Ngandi	ngan1295	0.005	3	33	0.97	0.44	0.10
170	Wik-Ngathan	wikn1245	0.007	231	13	0.46	0.93	0.10
767	Karajarri	kara1476	0.002	111	19	0.90	0.83	0.11
204	Wemba Wemba	wemb1241	0.004	97	20	0.91	0.79	0.11
611	Anguthimri	angu1242	0.014	7	28	0.76	0.43	0.11
821	Gumbaynggir	kumb1268	0.016	62	15	0.79	0.85	0.11
30	Duungidjawu	duun1241	0.010	43	19	0.83	0.80	0.11
979	Mawng	maun1240	0.001	86	22	1.00	0.81	0.11

Lex ID	Language	Glottolog	λ	x_{min}	Phonemes	Frac. of inventory	p	Goodness-of-fit
927	Tiwi	tiwi1244	0.001	89	20	1.00	0.87	0.11
81	Dalabon	ngal1292	0.002	580	18	0.62	0.85	0.11
1031	Ngadjunmaya	ngad1258	0.005	2	25	1.00	0.63	0.11
968	Ogh Angkula	ikar1243	0.010	30	23	0.96	0.62	0.11
851	Bilinarra	bili1250	0.002	162	19	0.83	0.80	0.11
857	Amurdak	amar1271	0.009	3	22	0.92	0.65	0.11
77	Dhangu	dhan1270	0.011	7	26	1.00	0.52	0.11
752	Kija	kitj1240	0.003	269	16	0.67	0.86	0.11
760	Kartujarra	kart1247	0.004	4	23	1.00	0.63	0.11
1003	Murrinh-patha	murr1259	0.011	7	25	1.00	0.53	0.11
943	Pintupi	pint1250	0.001	424	20	0.87	0.83	0.11
848	Wagiman	wage1238	0.004	130	22	0.81	0.63	0.11
917	Nyamal	nyam1271	0.004	2	22	0.92	0.63	0.11
978	Wotjobaluk	wotj1234	0.006	83	16	0.84	0.81	0.11
232	Warnman	wanm1242	0.003	202	9	0.39	0.95	0.12
946	Kurtjar	gurd1238	0.008	164	18	0.64	0.59	0.12
38	Djabugay	dyaa1242	0.004	188	12	0.63	0.90	0.12
650	Linngithigh	leni1238	0.011	2	28	0.97	0.34	0.12
31	Djinang	djin1253	0.002	24	25	1.00	0.57	0.12
620	Miriwoong	miri1266	0.002	538	14	0.70	0.91	0.12
800	Guwamu	guwa1243	0.008	3	21	1.00	0.62	0.12
941	Wambaya	nucl1328	0.002	5	22	0.96	0.61	0.12
631	Yalarnnga	yala1262	0.007	6	24	1.00	0.51	0.12
1024	Ngardily	west2437	0.009	3	23	1.00	0.53	0.12
790	Jaru	jaru1254	0.002	30	23	1.00	0.59	0.12
847	Ngalakgan	ngal1293	0.007	119	20	0.74	0.57	0.12
642	Mangala	mang1383	0.003	13	21	1.00	0.62	0.12
911	Nyawaygi	nyaw1247	0.006	67	15	0.83	0.81	0.12
923	Matngele	madn1237	0.007	109	18	0.72	0.64	0.12
62	Dhay'yi	dhal1246	0.003	228	10	0.38	0.66	0.12
737	Kukatj	guga1239	0.005	38	23	1.00	0.47	0.12
838	Gooniyandi	goon1238	0.002	15	23	1.00	0.49	0.12
967	Thaynakwithi	tyan1235	0.012	67	23	0.72	0.37	0.12
901	Guugu Yimidhirr	gugu1255	0.018	26	19	0.90	0.52	0.13
771	Kalkatungu	kalk1246	0.003	46	24	0.92	0.38	0.13
962	Gunya	guny1241	0.007	11	25	0.81	0.31	0.13
606	Mudburra	mudb1240	0.004	2	23	1.00	0.44	0.13
475	Southern Paakintyi	darl1243	0.003	175	13	0.50	0.68	0.13
853	Lardil	lard1243	0.002	50	25	1.00	0.40	0.13
972	Ogh Unyjan	kawa1290	0.013	31	22	0.88	0.31	0.13
546	Yanyuwa	yany1243	0.002	27	23	1.00	0.43	0.13
856	Patjtjamalh	wadj1254	0.004	2	25	0.93	0.26	0.13
1009	Gamilaraay	gami1243	0.005	20	21	1.00	0.41	0.13
99	Bidyara	bidy1243	0.004	7	19	1.00	0.52	0.13
778	Jiwarli	djiw1241	0.004	11	26	1.00	0.25	0.13
592	Muruwari	muru1266	0.003	63	22	0.88	0.32	0.13
1032	Kuku Yalanji	kuku1273	0.002	368	13	0.81	0.74	0.13
918	Atampaya	atam1239	0.008	1	25	1.00	0.24	0.14
237	Warluwarra	warl1256	0.004	22	26	0.79	0.17	0.14
1026	Gidabal	gida1240	0.004	283	14	0.70	0.60	0.14
200	Western Arrernte	west2441	0.012	8	25	1.00	0.21	0.14
206	Wayilwan	wayi1238	0.008	170	13	0.62	0.70	0.14

Lex ID	Language	Glottolog	λ	x_{min}	Phonemes	Frac. of inventory	p	Goodness-of-fit
288	Wangkatja	pini1245	0.002	48	23	1.00	0.30	0.14
940	Yawijibaya	yawi1239	0.013	9	18	0.78	0.39	0.14
958	Margany	marg1253	0.014	7	24	0.80	0.17	0.14
443	Payungu	bayu1240	0.006	5	26	1.00	0.18	0.14
949	Kok Nar	kokn1236	0.012	92	10	0.48	0.68	0.14
563	Ngarla	ngar1296	0.003	2	23	1.00	0.27	0.14
519	Nukunu	nugu1241	0.009	53	9	0.32	0.68	0.14
1019	Walmajarri	walm1241	0.001	226	20	0.87	0.39	0.14
957	Ngaanyatjarra	ngaa1240	0.002	57	23	1.00	0.25	0.14
910	Nungali	nung1291	0.007	114	14	0.88	0.64	0.14
919	Angkamuthi	angg1238	0.006	5	26	1.00	0.15	0.14
89	Bunuba	buna1275	0.006	7	24	1.00	0.20	0.14
1025	Waalubal	band1358	0.004	287	14	0.70	0.54	0.14
13	Dyirbal	dyir1250	0.005	59	16	1.00	0.50	0.15
985	Yaygir	yayg1236	0.005	107	17	0.85	0.36	0.15
762	Kariyarra	kari1304	0.010	2	24	1.00	0.17	0.15
252	Warlmanpa	warl1255	0.003	6	22	0.79	0.22	0.15
1016	Tharrkari	dhar1247	0.013	2	32	1.00	0.05	0.15
117	Wirangu	wira1265	0.008	3	23	1.00	0.19	0.15
598	Yandruwandha	yand1253	0.004	89	14	0.47	0.40	0.15
118	Badimaya	badi1246	0.008	3	24	1.00	0.17	0.15
417	Yindjibarndi	yind1247	0.005	17	25	1.00	0.15	0.15
982	Nhirrpi	nhir1234	0.017	1	30	1.00	0.06	0.15
787	Jawoyn	djau1244	0.003	63	27	1.00	0.11	0.15
920	Yadhaykenu	yadh1237	0.008	2	25	1.00	0.14	0.15
85	Burarra	bura1267	0.002	284	21	0.81	0.19	0.15
228	Warriyangga	wari1262	0.012	1	26	1.00	0.10	0.15
905	Malkana	malg1242	0.014	2	25	1.00	0.12	0.15
400	Thalanyji	dhal1245	0.004	161	7	0.27	0.59	0.15
929	Wanyjirra	wany1244	0.004	38	21	1.00	0.22	0.15
493	Nyiyaparli	nija1241	0.014	1	22	1.00	0.17	0.15
462	Panyjima	pany1241	0.005	106	8	0.32	0.59	0.15
113	Yulparija	yulp1239	0.002	151	20	0.87	0.22	0.15
835	Gugu Badhun	gugu1253	0.007	28	17	1.00	0.36	0.15
1008	Yuwaalaraay	yuwa1242	0.003	450	11	0.52	0.53	0.15
930	Purduna	burd1238	0.006	12	26	1.00	0.08	0.15
242	Warlpiri	warl1254	0.001	63	24	1.00	0.32	0.15
849	Waanyi	wany1247	0.002	211	15	0.75	0.39	0.15
433	Pitta Pitta	pitt1247	0.004	150	7	0.28	0.60	0.16
977	Warndarrang	wand1263	0.010	35	19	1.00	0.23	0.16
1029	Martuthunira	mart1255	0.004	8	26	1.00	0.08	0.16
1042	Thaayorre	thay1249	0.002	209	24	0.92	0.09	0.16
618	Mirniny	mirn1243	0.012	1	25	1.00	0.07	0.16
645	Malyangapa	maly1234	0.011	57	7	0.29	0.56	0.16
1030	Putijarra	pudi1238	0.003	1	25	1.00	0.06	0.16
734	Kukatja	kuka1246	0.001	35	23	1.00	0.22	0.16
269	Wangkumara	wong1246	0.006	4	30	1.00	0.02	0.16
554	Ngarluma	ngar1287	0.004	30	23	1.00	0.09	0.17
162	Adnyamathanha	adny1235	0.003	63	29	1.00	0.02	0.17
507	Nyangumarta	nyan1301	0.002	307	9	0.43	0.54	0.17
363	Yir Yoront	yiry1245	0.003	41	25	0.96	0.03	0.17
1007	Yuwaliyaay	yuwa1243	0.002	33	21	1.00	0.09	0.17

Lex ID	Language	Glottolog	λ	x_{min}	Phonemes	Frac. of inventory	p	Goodness-of-fit
865	Diyari	dier1241	0.015	13	23	0.92	0.04	0.17
866	Thirarri	dira1238	0.014	13	23	0.92	0.04	0.17
914	Yinhawangka	yinh1234	0.002	221	8	0.31	0.41	0.17
922	Nakara	naka1260	0.003	15	26	1.00	0.02	0.18
534	Nhanda	nhan1238	0.004	106	6	0.18	0.53	0.18
1006	Central Arrernte	mpar1238	0.004	27	25	1.00	0.02	0.18
1021	Watjarri	waja1257	0.004	5	26	1.00	0.02	0.18
101	Butchulla	baty1234	0.011	85	15	0.62	0.14	0.19
983	Nhangu	yann1237	0.003	130	19	0.61	0.02	0.19
91	Bularnu	bula1255	0.007	2	30	1.00	0.00	0.19
1040	Ngarinyin	ngar1284	0.001	2	27	1.00	0.04	0.20

Table S5.9. Comparing exponential and lognormal distributions using Vuong's likelihood ratio test.

* $p < 0.05$ after Bonferroni correction. ** $p < 0.01$ after Bonferroni correction.

lex ID	language variety	Glottolog	R	p	signif.
967	Thaynakwithi	tyan1235	3.84	0.000	*
81	Dalabon	ngal1292	3.80	0.000	*
923	Matngele	madn1237	2.56	0.005	
1007	Yuwaliyaay	yuwa1243	2.56	0.005	
30	Duungidjawu	duun1241	2.53	0.006	
650	Linngithigh	leni1238	2.47	0.007	
38	Djabugay	dyaa1242	2.44	0.007	
621	Mengerrdji	mang1382	2.44	0.007	
493	Nyiyaparli	nija1241	2.40	0.008	
1008	Yuwaalaraay	yuwa1242	2.38	0.009	
911	Nyawaygi	nyaw1247	2.35	0.009	
206	Wayilwan	wayi1238	2.34	0.010	
305	Western Wakaya	waga1260	2.14	0.016	
113	Yulparija	yulp1239	2.06	0.020	
620	Miriwoong	miri1266	2.03	0.021	
847	Ngalakgan	ngal1293	1.99	0.023	
853	Lardil	lard1243	1.97	0.025	
232	Warnman	wanm1242	1.90	0.028	
12	Erre	erre1238	1.90	0.029	
563	Ngarla	ngar1296	1.89	0.029	
851	Bilinarra	bili1250	1.88	0.030	
852	Gurindji	guri1247	1.87	0.031	
565	Ngarinyman	ngar1235	1.86	0.032	
170	Wik-Ngathan	wikn1245	1.85	0.032	
928	Bakanh	paka1251	1.81	0.035	
1019	Walmajarri	walm1241	1.79	0.037	
964	Olkol	ulku1238	1.73	0.042	
848	Wagiman	wage1238	1.70	0.044	
752	Kija	kitj1240	1.70	0.045	
1012	Warrgamay	warr1255	1.67	0.047	
863	Marra	mara1385	1.64	0.051	
1030	Putijarra	pudi1238	1.64	0.051	
965	Oykangand	oyka1239	1.62	0.052	
1009	Gamilaraay	gami1243	1.61	0.054	
734	Kukatja	kuka1246	1.60	0.055	
1011	Yorta Yorta	yort1237	1.50	0.067	
606	Mudburra	mudb1240	1.50	0.067	
949	Kok Nar	kokn1236	1.47	0.070	
941	Wambaya	nucl1328	1.46	0.072	
767	Karajarri	kara1476	1.43	0.076	
31	Djinang	djin1253	1.37	0.085	
787	Jawoyn	djau1244	1.28	0.100	
934	Urningangg	urni1239	1.26	0.104	
760	Kartujarra	kart1247	1.21	0.113	
1001	Ngandi	ngan1295	1.17	0.121	
838	Gooniyandi	goon1238	1.15	0.125	
790	Jaru	jaru1254	1.14	0.128	
1023	Wubuy	nung1290	1.11	0.134	

lex ID	language variety	Glottolog	R	p	signif.
921	Gurr-Goni	gura1252	1.08	0.141	
762	Kariyarra	kari1304	1.07	0.143	
1021	Watjarri	waja1257	1.05	0.147	
800	Guwamu	guwa1243	1.05	0.147	
856	Patjtjamalh	wadj1254	1.01	0.155	
1031	Ngadjunmaya	ngad1258	1.00	0.158	
978	Wotjobaluk	wotj1234	0.98	0.165	
952	Ngawun	ngaw1240	0.94	0.173	
737	Kukatj	guga1239	0.94	0.173	
1002	Yintyingka	ayab1239	0.94	0.174	
849	Waanyi	wany1247	0.92	0.178	
507	Nyangumarta	nyan1301	0.90	0.185	
914	Yinhawangka	yinh1234	0.89	0.186	
377	Umpila	umpi1239	0.88	0.189	
1042	Thaayorre	thay1249	0.88	0.190	
972	Ogh Unyjan	kawa1290	0.87	0.191	
966	Wik Mungkan	wikm1247	0.82	0.207	
1024	Ngardily	west2437	0.81	0.209	
917	Nyamal	nyam1271	0.80	0.213	
540	Ngiyambaa	wang1291	0.78	0.217	
363	Yir Yoront	yiry1245	0.77	0.220	
462	Panyjima	pany1241	0.77	0.221	
1003	Murrinh-patha	murr1259	0.75	0.228	
857	Amurdak	amar1271	0.74	0.230	
495	Nyikina	nyig1240	0.74	0.230	
1043	Mbabaram	mbab1239	0.73	0.233	
910	Nungali	nung1291	0.69	0.244	
242	Warlpiri	warl1254	0.66	0.256	
996	Emmi	amii1238	0.66	0.256	
99	Bidyara	bidy1243	0.64	0.260	
642	Mangala	mang1383	0.64	0.261	
1025	Waalubal	band1358	0.63	0.266	
1026	Gidabal	gida1240	0.62	0.267	
618	Mirniny	mirn1243	0.62	0.267	
926	Limilngan	nucl1327	0.62	0.267	
94	Biri	biri1256	0.60	0.274	
945	Wiri	biri1256	0.57	0.284	
979	Mawng	maun1240	0.57	0.285	
228	Warriyangga	wari1262	0.54	0.293	
740	Kugu Nganhcara	wikn1246	0.53	0.300	
929	Wanyjirra	wany1244	0.51	0.305	
841	Worrorra	woro1258	0.49	0.312	
935	Rembarrnga	remb1249	0.48	0.317	
631	Yalarnnga	yala1262	0.47	0.318	
697	Kurrama	kurr1243	0.47	0.320	
985	Yaygir	yayg1236	0.47	0.321	
5	Gangulu	gang1268	0.44	0.330	
13	Dyirbal	dyir1250	0.39	0.349	
918	Atampaya	atam1239	0.39	0.350	
1040	Ngarinyin	ngar1284	0.37	0.355	
845	Iwaidja	iwai1244	0.35	0.363	
105	Bardi	bard1255	0.33	0.369	

lex ID	language variety	Glottolog	*R*	*p*	signif.
118	Badimaya	badi1246	0.33	0.372	
915	Alawa	alaw1244	0.32	0.374	
265	Wardaman	ward1246	0.32	0.375	
1029	Martuthunira	mart1255	0.27	0.393	
835	Gugu Badhun	gugu1253	0.25	0.401	
546	Yanyuwa	yany1243	0.25	0.403	
204	Wemba Wemba	wemb1241	0.24	0.404	
63	Dharumbal	dhar1248	0.22	0.413	
417	Yindjibarndi	yind1247	0.21	0.416	
778	Jiwarli	djiw1241	0.21	0.416	
433	Pitta Pitta	pitt1247	0.21	0.417	
89	Bunuba	buna1275	0.16	0.435	
946	Kurtjar	gurd1238	0.16	0.436	
645	Malyangapa	maly1234	0.15	0.440	
905	Malkana	malg1242	0.14	0.446	
443	Payungu	bayu1240	0.13	0.447	
412	Ritharrngu	rita1239	0.11	0.456	
400	Thalanyji	dhal1245	0.10	0.462	
117	Wirangu	wira1265	0.09	0.465	
101	Butchulla	baty1234	0.07	0.471	
288	Wangkatja	pini1245	0.02	0.493	
925	Larrakia	lara1258	0.00	0.500	
957	Ngaanyatjarra	ngaa1240	-0.01	0.506	
656	Kuugu Ya'u	kuuk1238	-0.01	0.506	
920	Yadhaykenu	yadh1237	-0.02	0.508	
427	Yidiny	yidi1250	-0.03	0.513	
1018	Djapu	djap1238	-0.04	0.515	
821	Gumbaynggir	kumb1268	-0.08	0.530	
62	Dhay'yi	dhal1246	-0.10	0.541	
807	Gupapuyngu	gupa1247	-0.11	0.544	
77	Dhangu	dhan1270	-0.12	0.547	
982	Nhirrpi	nhir1234	-0.12	0.549	
968	Ogh Angkula	ikar1243	-0.15	0.561	
901	Guugu Yimidhirr	gugu1255	-0.18	0.571	
519	Nukunu	nugu1241	-0.21	0.582	
939	Unggumi	ungg1243	-0.25	0.600	
919	Angkamuthi	angg1238	-0.26	0.602	
1032	Kuku Yalanji	kuku1273	-0.26	0.604	
85	Burarra	bura1267	-0.27	0.607	
940	Yawijibaya	yawi1239	-0.34	0.634	
930	Purduna	burd1238	-0.35	0.635	
927	Tiwi	tiwi1244	-0.35	0.636	
977	Warndarrang	wand1263	-0.36	0.640	
598	Yandruwandha	yand1253	-0.38	0.646	
592	Muruwari	muru1266	-0.39	0.653	
611	Anguthimri	angu1242	-0.44	0.669	
534	Nhanda	nhan1238	-0.45	0.674	
554	Ngarluma	ngar1287	-0.46	0.678	
922	Nakara	naka1260	-0.47	0.681	
744	Koko Bera	gugu1254	-0.50	0.690	
252	Warlmanpa	warl1255	-0.55	0.709	
943	Pintupi	pint1250	-0.57	0.715	

lex ID	language variety	Glottolog	R	p	signif.
91	Bularnu	bula1255	-0.59	0.723	
771	Kalkatungu	kalk1246	-0.60	0.724	
269	Wangkumara	wong1246	-0.64	0.740	
958	Margany	marg1253	-0.66	0.745	
237	Warluwarra	warl1256	-0.67	0.750	
983	Nhangu	yann1237	-0.72	0.765	
1016	Tharrkari	dhar1247	-0.85	0.803	
475	Southern Paakintyi	darl1243	-0.88	0.812	
962	Gunya	guny1241	-1.02	0.846	
865	Diyari	dier1241	-1.06	0.855	
866	Thirarri	dira1238	-1.06	0.855	
200	Western Arrernte	west2441	-1.07	0.857	
162	Adnyamathanha	adny1235	-1.34	0.910	
1006	Central Arrernte	mpar1238	-1.58	0.943	

Table S5.10. Comparing exponential and lognormal distributions with x_{min} using Vuong's likelihood ratio test.

Part A. Using x_{min} from the exponential fit. $R > 0$ favours exponential.

* $p < 0.05$ after Bonferroni correction. ** $p < 0.01$ after Bonferroni correction.

lex ID	language variety	Glottolog	R	p	signif.
650	Linngithigh	leni1238	3.71	0.000	
856	Patjtjamalh	wadj1254	2.68	0.004	
1007	Yuwaliyaay	yuwa1243	2.56	0.005	
493	Nyiyaparli	nija1241	2.40	0.008	
305	Western Wakaya	waga1260	2.14	0.016	
853	Lardil	lard1243	1.97	0.025	
12	Erre	erre1238	1.90	0.029	
563	Ngarla	ngar1296	1.89	0.029	
964	Olkol	ulku1238	1.73	0.042	
1030	Putijarra	pudi1238	1.64	0.051	
252	Warlmanpa	warl1255	1.63	0.052	
1009	Gamilaraay	gami1243	1.61	0.054	
734	Kukatja	kuka1246	1.60	0.055	
917	Nyamal	nyam1271	1.51	0.065	
606	Mudburra	mudb1240	1.50	0.067	
1001	Ngandi	ngan1295	1.40	0.080	
941	Wambaya	nucl1328	1.40	0.081	
540	Ngiyambaa	wang1291	1.39	0.082	
31	Djinang	djin1253	1.37	0.085	
965	Oykangand	oyka1239	1.37	0.086	
787	Jawoyn	djau1244	1.28	0.100	
978	Wotjobaluk	wotj1234	1.26	0.104	
852	Gurindji	guri1247	1.24	0.108	
760	Kartujarra	kart1247	1.21	0.113	
838	Gooniyandi	goon1238	1.15	0.125	
790	Jaru	jaru1254	1.14	0.128	
857	Amurdak	amar1271	1.10	0.135	
762	Kariyarra	kari1304	1.07	0.143	
1023	Wubuy	nung1290	1.05	0.146	
1021	Watjarri	waja1257	1.05	0.147	
800	Guwamu	guwa1243	1.05	0.147	
1031	Ngadjunmaya	ngad1258	1.00	0.158	
737	Kukatj	guga1239	0.94	0.173	
926	Limilngan	nucl1327	0.88	0.190	
1042	Thaayorre	thay1249	0.88	0.190	
940	Yawijibaya	yawi1239	0.86	0.195	
1043	Mbabaram	mbab1239	0.86	0.195	
1002	Yintyingka	ayab1239	0.81	0.209	
1024	Ngardily	west2437	0.81	0.209	
1003	Murrinh-patha	murr1259	0.75	0.228	
495	Nyikina	nyig1240	0.71	0.238	
952	Ngawun	ngaw1240	0.70	0.241	
62	Dhay'yi	dhal1246	0.68	0.250	
427	Yidiny	yidi1250	0.66	0.255	
242	Warlpiri	warl1254	0.66	0.256	
996	Emmi	amii1238	0.66	0.256	
99	Bidyara	bidy1243	0.64	0.260	

lex ID	language variety	Glottolog	R	p	signif.
642	Mangala	mang1383	0.64	0.261	
105	Bardi	bard1255	0.63	0.266	
618	Mirniny	mirn1243	0.62	0.267	
697	Kurrama	kurr1243	0.62	0.267	
94	Biri	biri1256	0.60	0.274	
81	Dalabon	ngal1292	0.57	0.284	
945	Wiri	biri1256	0.57	0.284	
979	Mawng	maun1240	0.57	0.285	
925	Larrakia	lara1258	0.56	0.287	
228	Warriyangga	wari1262	0.54	0.293	
611	Anguthimri	angu1242	0.53	0.299	
929	Wanyjirra	wany1244	0.51	0.305	
400	Thalanyji	dhal1245	0.50	0.309	
645	Malyangapa	maly1234	0.49	0.311	
30	Duungidjawu	duun1241	0.48	0.317	
631	Yalarnnga	yala1262	0.47	0.318	
5	Gangulu	gang1268	0.44	0.330	
946	Kurtjar	gurd1238	0.44	0.330	
63	Dharumbal	dhar1248	0.42	0.336	
934	Urningangg	urni1239	0.39	0.348	
13	Dyirbal	dyir1250	0.39	0.349	
918	Atampaya	atam1239	0.39	0.350	
1040	Ngarinyin	ngar1284	0.37	0.355	
845	Iwaidja	iwai1244	0.35	0.363	
118	Badimaya	badi1246	0.33	0.372	
740	Kugu Nganhcara	wikn1246	0.31	0.378	
923	Matngele	madn1237	0.31	0.379	
972	Ogh Unyjan	kawa1290	0.28	0.391	
1029	Martuthunira	mart1255	0.27	0.393	
835	Gugu Badhun	gugu1253	0.25	0.401	
546	Yanyuwa	yany1243	0.25	0.403	
621	Mengerrdji	mang1382	0.24	0.404	
433	Pitta Pitta	pitt1247	0.24	0.405	
363	Yir Yoront	yiry1245	0.21	0.416	
417	Yindjibarndi	yind1247	0.21	0.416	
778	Jiwarli	djiw1241	0.21	0.416	
968	Ogh Angkula	ikar1243	0.20	0.419	
901	Guugu Yimidhirr	gugu1255	0.20	0.422	
821	Gumbaynggir	kumb1268	0.20	0.423	
1012	Warrgamay	warr1255	0.19	0.423	
915	Alawa	alaw1244	0.18	0.429	
752	Kija	kitj1240	0.17	0.434	
89	Bunuba	buna1275	0.16	0.435	
905	Malkana	malg1242	0.14	0.446	
443	Payungu	bayu1240	0.13	0.447	
911	Nyawaygi	nyaw1247	0.13	0.448	
232	Warnman	wanm1242	0.12	0.453	
620	Miriwoong	miri1266	0.11	0.454	
38	Djabugay	dyaa1242	0.10	0.462	
412	Ritharrngu	rita1239	0.09	0.462	
117	Wirangu	wira1265	0.09	0.465	
949	Kok Nar	kokn1236	0.08	0.469	

lex ID	language variety	Glottolog	R	p	signif.
534	Nhanda	nhan1238	0.05	0.482	
507	Nyangumarta	nyan1301	0.04	0.485	
288	Wangkatja	pini1245	0.02	0.493	
1025	Waalubal	band1358	0.00	0.500	
957	Ngaanyatjarra	ngaa1240	-0.01	0.506	
920	Yadhaykenu	yadh1237	-0.02	0.508	
744	Koko Bera	gugu1254	-0.04	0.515	
1018	Djapu	djap1238	-0.04	0.515	
914	Yinhawangka	yinh1234	-0.04	0.516	
204	Wemba Wemba	wemb1241	-0.06	0.523	
170	Wik-Ngathan	wikn1245	-0.08	0.533	
939	Unggumi	ungg1243	-0.10	0.538	
807	Gupapuyngu	gupa1247	-0.11	0.544	
77	Dhangu	dhan1270	-0.12	0.547	
982	Nhirrpi	nhir1234	-0.12	0.549	
1026	Gidabal	gida1240	-0.13	0.552	
1011	Yorta Yorta	yort1237	-0.13	0.553	
377	Umpila	umpi1239	-0.14	0.555	
985	Yaygir	yayg1236	-0.18	0.573	
1032	Kuku Yalanji	kuku1273	-0.20	0.578	
935	Rembarrnga	remb1249	-0.21	0.582	
592	Muruwari	muru1266	-0.21	0.583	
462	Panyjima	pany1241	-0.21	0.584	
848	Wagiman	wage1238	-0.21	0.585	
863	Marra	mara1385	-0.23	0.591	
919	Angkamuthi	angg1238	-0.26	0.602	
206	Wayilwan	wayi1238	-0.27	0.606	
519	Nukunu	nugu1241	-0.29	0.616	
656	Kuugu Ya'u	kuuk1238	-0.31	0.620	
767	Karajarri	kara1476	-0.31	0.621	
928	Bakanh	paka1251	-0.32	0.626	
910	Nungali	nung1291	-0.32	0.627	
930	Purduna	burd1238	-0.35	0.635	
927	Tiwi	tiwi1244	-0.35	0.636	
475	Southern Paakintyi	darl1243	-0.35	0.638	
977	Warndarrang	wand1263	-0.36	0.640	
598	Yandruwandha	yand1253	-0.42	0.662	
265	Wardaman	ward1246	-0.43	0.668	
554	Ngarluma	ngar1287	-0.46	0.678	
983	Nhangu	yann1237	-0.46	0.679	
851	Bilinarra	bili1250	-0.47	0.681	
922	Nakara	naka1260	-0.47	0.681	
966	Wik Mungkan	wikm1247	-0.48	0.684	
962	Gunya	guny1241	-0.49	0.687	
958	Margany	marg1253	-0.49	0.689	
565	Ngarinyman	ngar1235	-0.52	0.699	
967	Thaynakwithi	tyan1235	-0.57	0.714	
921	Gurr-Goni	gura1252	-0.57	0.715	
849	Waanyi	wany1247	-0.58	0.720	
847	Ngalakgan	ngal1293	-0.59	0.721	
91	Bularnu	bula1255	-0.59	0.723	
269	Wangkumara	wong1246	-0.64	0.740	

lex ID	language variety	Glottolog	R	p	signif.
841	Worrorra	woro1258	-0.74	0.771	
237	Warluwarra	warl1256	-0.78	0.782	
101	Butchulla	baty1234	-0.79	0.786	
85	Burarra	bura1267	-0.81	0.791	
113	Yulparija	yulp1239	-0.81	0.792	
943	Pintupi	pint1250	-0.84	0.799	
1016	Tharrkari	dhar1247	-0.85	0.803	
1008	Yuwaalaraay	yuwa1242	-0.90	0.817	
865	Diyari	dier1241	-0.98	0.837	
866	Thirarri	dira1238	-0.99	0.839	
1019	Walmajarri	walm1241	-1.00	0.841	
771	Kalkatungu	kalk1246	-1.05	0.853	
200	Western Arrernte	west2441	-1.07	0.857	
162	Adnyamathanha	adny1235	-1.34	0.910	
1006	Central Arrernte	mpar1238	-1.58	0.943	

Table S5.10. Comparing exponential and lognormal distributions with x_{min} using Vuong's likelihood ratio test.

Part B. Using x_{min} from the lognormal fit. $R > 0$ favours lognormal.

* $p < 0.05$ after Bonferroni correction. ** $p < 0.01$ after Bonferroni correction.

Lex ID	Language	Glottolog	R	p	Signif.
1021	Watjarri	waja1257	1.44	0.074	
91	Bularnu	bula1255	1.40	0.080	
762	Kariyarra	kari1304	1.37	0.085	
919	Angkamuthi	angg1238	1.36	0.087	
162	Adnyamathanha	adny1235	1.34	0.090	
1016	Tharrkari	dhar1247	1.32	0.093	
1030	Putijarra	pudi1238	1.26	0.103	
905	Malkana	malg1242	1.25	0.106	
1006	Central Arrernte	mpar1238	1.24	0.108	
400	Thalanyji	dhal1245	1.23	0.109	
957	Ngaanyatjarra	ngaa1240	1.21	0.113	
519	Nukunu	nugu1241	1.20	0.115	
288	Wangkatja	pini1245	1.17	0.121	
228	Warriyangga	wari1262	1.17	0.122	
507	Nyangumarta	nyan1301	1.16	0.123	
200	Western Arrernte	west2441	1.14	0.128	
443	Payungu	bayu1240	1.11	0.133	
269	Wangkumara	wong1246	1.08	0.139	
866	Thirarri	dira1238	1.06	0.145	
865	Diyari	dier1241	1.06	0.145	
242	Warlpiri	warl1254	1.06	0.146	
363	Yir Yoront	yiry1245	1.05	0.146	
778	Jiwarli	djiw1241	1.05	0.147	
922	Nakara	naka1260	1.05	0.148	
645	Malyangapa	maly1234	1.05	0.148	
534	Nhanda	nhan1238	1.02	0.154	
940	Yawijibaya	yawi1239	1.01	0.156	
563	Ngarla	ngar1296	1.01	0.156	
943	Pintupi	pint1250	1.01	0.157	
598	Yandruwandha	yand1253	1.00	0.158	
1019	Walmajarri	walm1241	1.00	0.159	
838	Gooniyandi	goon1238	0.99	0.161	
417	Yindjibarndi	yind1247	0.99	0.162	
1024	Ngardily	west2437	0.98	0.163	
918	Atampaya	atam1239	0.96	0.168	
771	Kalkatungu	kalk1246	0.94	0.173	
734	Kukatja	kuka1246	0.94	0.173	
807	Gupapuyngu	gupa1247	0.93	0.175	
967	Thaynakwithi	tyan1235	0.93	0.177	
985	Yaygir	yayg1236	0.89	0.186	
982	Nhirrpi	nhir1234	0.89	0.187	
917	Nyamal	nyam1271	0.87	0.193	
493	Nyiyaparli	nija1241	0.83	0.203	
642	Mangala	mang1383	0.83	0.204	
31	Djinang	djin1253	0.82	0.206	
118	Badimaya	badi1246	0.81	0.208	
81	Dalabon	ngal1292	0.80	0.212	

**Lex ID**	**Language**	**Glottolog**	R	p	**Signif.**
929	Wanyjirra	wany1244	0.80	0.213	
117	Wirangu	wira1265	0.80	0.213	
475	Southern Paakintyi	darl1243	0.79	0.214	
101	Butchulla	baty1234	0.79	0.214	
606	Mudburra	mudb1240	0.79	0.215	
85	Burarra	bura1267	0.79	0.215	
237	Warluwarra	warl1256	0.78	0.218	
845	Iwaidja	iwai1244	0.77	0.219	
1031	Ngadjunmaya	ngad1258	0.75	0.226	
611	Anguthimri	angu1242	0.75	0.227	
841	Worrorra	woro1258	0.74	0.229	
849	Waanyi	wany1247	0.73	0.233	
983	Nhangu	yann1237	0.72	0.235	
618	Mirniny	mirn1243	0.70	0.243	
946	Kurtjar	gurd1238	0.66	0.256	
767	Karajarri	kara1476	0.65	0.259	
113	Yulparija	yulp1239	0.64	0.260	
1011	Yorta Yorta	yort1237	0.63	0.265	
863	Marra	mara1385	0.63	0.265	
737	Kukatj	guga1239	0.63	0.266	
939	Unggumi	ungg1243	0.62	0.266	
252	Warlmanpa	warl1255	0.62	0.267	
697	Kurrama	kurr1243	0.62	0.267	
941	Wambaya	nucl1328	0.61	0.269	
927	Tiwi	tiwi1244	0.61	0.272	
99	Bidyara	bidy1243	0.60	0.273	
740	Kugu Nganhcara	wikn1246	0.59	0.277	
592	Muruwari	muru1266	0.59	0.277	
1001	Ngandi	ngan1295	0.58	0.280	
921	Gurr-Goni	gura1252	0.57	0.285	
170	Wik-Ngathan	wikn1245	0.55	0.290	
848	Wagiman	wage1238	0.55	0.292	
930	Purduna	burd1238	0.54	0.293	
857	Amurdak	amar1271	0.54	0.294	
412	Ritharrngu	rita1239	0.54	0.295	
656	Kuugu Ya'u	kuuk1238	0.54	0.296	
565	Ngarinyman	ngar1235	0.52	0.301	
744	Koko Bera	gugu1254	0.52	0.302	
1025	Waalubal	band1358	0.51	0.305	
265	Wardaman	ward1246	0.50	0.310	
935	Rembarrnga	remb1249	0.49	0.314	
966	Wik Mungkan	wikm1247	0.48	0.316	
977	Warndarrang	wand1263	0.48	0.317	
847	Ngalakgan	ngal1293	0.47	0.318	
851	Bilinarra	bili1250	0.47	0.319	
1008	Yuwaalaraay	yuwa1242	0.46	0.322	
650	Linngithigh	leni1238	0.46	0.322	
620	Miriwoong	miri1266	0.46	0.323	
923	Matngele	madn1237	0.45	0.325	
1040	Ngarinyin	ngar1284	0.43	0.333	
787	Jawoyn	djau1244	0.41	0.341	
621	Mengerrdji	mang1382	0.40	0.344	

Lex ID	Language	Glottolog	R	p	Signif.
760	Kartujarra	kart1247	0.38	0.351	
958	Margany	marg1253	0.38	0.352	
962	Gunya	guny1241	0.38	0.352	
1043	Mbabaram	mbab1239	0.36	0.361	
952	Ngawun	ngaw1240	0.35	0.362	
1018	Djapu	djap1238	0.35	0.364	
945	Wiri	biri1256	0.34	0.368	
928	Bakanh	paka1251	0.33	0.371	
910	Nungali	nung1291	0.32	0.373	
554	Ngarluma	ngar1287	0.31	0.379	
12	Erre	erre1238	0.31	0.380	
1026	Gidabal	gida1240	0.30	0.382	
206	Wayilwan	wayi1238	0.27	0.394	
1032	Kuku Yalanji	kuku1273	0.26	0.396	
790	Jaru	jaru1254	0.23	0.408	
105	Bardi	bard1255	0.21	0.415	
462	Panyjima	pany1241	0.21	0.416	
901	Guugu Yimidhirr	gugu1255	0.18	0.429	
1003	Murrinh-patha	murr1259	0.18	0.430	
852	Gurindji	guri1247	0.16	0.436	
968	Ogh Angkula	ikar1243	0.15	0.439	
5	Gangulu	gang1268	0.14	0.443	
204	Wemba Wemba	wemb1241	0.14	0.443	
30	Duungidjawu	duun1241	0.14	0.444	
377	Umpila	umpi1239	0.14	0.445	
1023	Wubuy	nung1290	0.12	0.451	
77	Dhangu	dhan1270	0.12	0.453	
62	Dhay'yi	dhal1246	0.10	0.459	
979	Mawng	maun1240	0.10	0.460	
89	Bunuba	buna1275	0.08	0.467	
821	Gumbaynggir	kumb1268	0.08	0.470	
1029	Martuthunira	mart1255	0.07	0.471	
631	Yalarnnga	yala1262	0.05	0.478	
856	Patjtjamalh	wadj1254	0.04	0.483	
914	Yinhawangka	yinh1234	0.04	0.484	
540	Ngiyambaa	wang1291	0.03	0.488	
920	Yadhaykenu	yadh1237	0.02	0.492	
800	Guwamu	guwa1243	0.01	0.495	
925	Larrakia	lara1258	0.00	0.498	
13	Dyirbal	dyir1250	0.00	0.499	
996	Emmi	amii1238	-0.02	0.507	
94	Biri	biri1256	-0.08	0.531	
38	Djabugay	dyaa1242	-0.10	0.538	
495	Nyikina	nyig1240	-0.11	0.544	
232	Warnman	wanm1242	-0.12	0.547	
911	Nyawaygi	nyaw1247	-0.13	0.552	
853	Lardil	lard1243	-0.13	0.553	
972	Ogh Unyjan	kawa1290	-0.15	0.559	
752	Kija	kitj1240	-0.17	0.566	
915	Alawa	alaw1244	-0.18	0.571	
1012	Warrgamay	warr1255	-0.18	0.571	
934	Urningangg	urni1239	-0.19	0.577	

Lex ID	Language	Glottolog	*R*	*p*	Signif.
433	Pitta Pitta	pitt1247	-0.21	0.583	
978	Wotjobaluk	wotj1234	-0.24	0.595	
546	Yanyuwa	yany1243	-0.25	0.597	
835	Gugu Badhun	gugu1253	-0.25	0.599	
305	Western Wakaya	waga1260	-0.28	0.610	
1002	Yintyingka	ayab1239	-0.34	0.633	
949	Kok Nar	kokn1236	-0.35	0.638	
926	Limilngan	nucl1327	-0.42	0.664	
63	Dharumbal	dhar1248	-0.43	0.668	
1042	Thaayorre	thay1249	-0.50	0.691	
965	Oykangand	oyka1239	-0.63	0.736	
427	Yidiny	yidi1250	-0.66	0.745	
1009	Gamilaraay	gami1243	-1.61	0.946	
964	Olkol	ulku1238	-1.75	0.960	
1007	Yuwaliyaay	yuwa1243	-2.56	0.995	

Table S5.11. Comparing power law and exponential distributions with x_{min} using Vuong's likelihood ratio test.

Part A. Using x_{min} from the power law fit. $R > 0$ favours power law.

* $p < 0.05$ after Bonferroni correction. ** $p < 0.01$ after Bonferroni correction.

Lex ID	Language	Glottolog	R	p	Signif.
1042	Thaayorre	thay1249	2.27	0.012	
982	Nhirrpi	nhir1234	1.80	0.036	
200	Western Arrernte	west2441	1.72	0.043	
1016	Tharrkari	dhar1247	1.70	0.045	
905	Malkana	malg1242	1.59	0.056	
1007	Yuwaliyaay	yuwa1243	1.58	0.057	
162	Adnyamathanha	adny1235	1.56	0.059	
534	Nhanda	nhan1238	1.54	0.061	
762	Kariyarra	kari1304	1.49	0.069	
790	Jaru	jaru1254	1.41	0.080	
1040	Ngarinyin	ngar1284	1.37	0.086	
935	Rembarrnga	remb1249	1.31	0.095	
1021	Watjarri	waja1257	1.26	0.104	
919	Angkamuthi	angg1238	1.25	0.106	
620	Miriwoong	miri1266	1.25	0.106	
554	Ngarluma	ngar1287	1.24	0.107	
89	Bunuba	buna1275	1.24	0.108	
930	Purduna	burd1238	1.24	0.108	
847	Ngalakgan	ngal1293	1.21	0.114	
1006	Central Arrernte	mpar1238	1.21	0.114	
918	Atampaya	atam1239	1.19	0.117	
1031	Ngadjunmaya	ngad1258	1.12	0.130	
1029	Martuthunira	mart1255	1.12	0.132	
920	Yadhaykenu	yadh1237	1.11	0.133	
443	Payungu	bayu1240	1.10	0.136	
400	Thalanyji	dhal1245	1.09	0.137	
91	Bularnu	bula1255	1.05	0.147	
968	Ogh Angkula	ikar1243	1.05	0.148	
914	Yinhawangka	yinh1234	1.04	0.150	
81	Dalabon	ngal1292	1.03	0.151	
1030	Putijarra	pudi1238	1.02	0.154	
563	Ngarla	ngar1296	1.00	0.158	
778	Jiwarli	djiw1241	0.99	0.160	
288	Wangkatja	pini1245	0.99	0.161	
1008	Yuwaalaraay	yuwa1242	0.98	0.163	
940	Yawijibaya	yawi1239	0.97	0.166	
519	Nukunu	nugu1241	0.96	0.168	
957	Ngaanyatjarra	ngaa1240	0.93	0.176	
118	Badimaya	badi1246	0.92	0.180	
841	Worrorra	woro1258	0.91	0.181	
228	Warriyangga	wari1262	0.90	0.184	
1019	Walmajarri	walm1241	0.89	0.186	
958	Margany	marg1253	0.89	0.186	
985	Yaygir	yayg1236	0.89	0.186	
1009	Gamilaraay	gami1243	0.88	0.190	
507	Nyangumarta	nyan1301	0.87	0.191	
853	Lardil	lard1243	0.87	0.193	

Lex ID	Language	Glottolog	R	p	Signif.
1001	Ngandi	ngan1295	0.86	0.194	
917	Nyamal	nyam1271	0.86	0.195	
787	Jawoyn	djau1244	0.84	0.199	
631	Yalarnnga	yala1262	0.82	0.206	
943	Pintupi	pint1250	0.82	0.207	
939	Unggumi	ungg1243	0.80	0.212	
433	Pitta Pitta	pitt1247	0.80	0.213	
606	Mudburra	mudb1240	0.79	0.214	
972	Ogh Unyjan	kawa1290	0.75	0.226	
1043	Mbabaram	mbab1239	0.73	0.232	
1024	Ngardily	west2437	0.73	0.234	
237	Warluwarra	warl1256	0.72	0.236	
117	Wirangu	wira1265	0.71	0.239	
242	Warlpiri	warl1254	0.71	0.239	
1011	Yorta Yorta	yort1237	0.71	0.240	
945	Wiri	biri1256	0.69	0.244	
642	Mangala	mang1383	0.68	0.247	
921	Gurr-Goni	gura1252	0.68	0.248	
929	Wanyjirra	wany1244	0.68	0.250	
13	Dyirbal	dyir1250	0.66	0.256	
922	Nakara	naka1260	0.65	0.257	
845	Iwaidja	iwai1244	0.65	0.258	
417	Yindjibarndi	yind1247	0.65	0.259	
618	Mirniny	mirn1243	0.64	0.263	
101	Butchulla	baty1234	0.63	0.265	
269	Wangkumara	wong1246	0.62	0.268	
85	Burarra	bura1267	0.62	0.269	
849	Waanyi	wany1247	0.61	0.271	
645	Malyangapa	maly1234	0.60	0.275	
767	Karajarri	kara1476	0.60	0.275	
979	Mawng	maun1240	0.60	0.275	
852	Gurindji	guri1247	0.59	0.277	
493	Nyiyaparli	nija1241	0.59	0.278	
462	Panyjima	pany1241	0.58	0.280	
856	Patjtjamalh	wadj1254	0.57	0.285	
206	Wayilwan	wayi1238	0.56	0.287	
866	Thirarri	dira1238	0.55	0.292	
983	Nhangu	yann1237	0.54	0.296	
865	Diyari	dier1241	0.54	0.296	
838	Gooniyandi	goon1238	0.52	0.301	
232	Warnman	wanm1242	0.51	0.304	
857	Amurdak	amar1271	0.51	0.304	
941	Wambaya	nucl1328	0.51	0.305	
77	Dhangu	dhan1270	0.46	0.324	
949	Kok Nar	kokn1236	0.45	0.326	
475	Southern Paakintyi	darl1243	0.44	0.330	
927	Tiwi	tiwi1244	0.44	0.331	
31	Djinang	djin1253	0.43	0.332	
113	Yulparija	yulp1239	0.43	0.334	
835	Gugu Badhun	gugu1253	0.42	0.338	
377	Umpila	umpi1239	0.40	0.346	
863	Marra	mara1385	0.37	0.354	

Lex ID	Language	Glottolog	R	p	Signif.
807	Gupapuyngu	gupa1247	0.37	0.355	
1003	Murrinh-patha	murr1259	0.36	0.359	
967	Thaynakwithi	tyan1235	0.36	0.359	
740	Kugu Nganhcara	wikn1246	0.36	0.361	
977	Warndarrang	wand1263	0.34	0.367	
848	Wagiman	wage1238	0.34	0.367	
760	Kartujarra	kart1247	0.33	0.370	
5	Gangulu	gang1268	0.32	0.374	
63	Dharumbal	dhar1248	0.29	0.386	
592	Muruwari	muru1266	0.28	0.389	
611	Anguthimri	angu1242	0.28	0.391	
204	Wemba Wemba	wemb1241	0.27	0.394	
1023	Wubuy	nung1290	0.27	0.395	
565	Ngarinyman	ngar1235	0.26	0.396	
851	Bilinarra	bili1250	0.26	0.399	
966	Wik Mungkan	wikm1247	0.25	0.402	
697	Kurrama	kurr1243	0.24	0.405	
952	Ngawun	ngaw1240	0.22	0.412	
412	Ritharrngu	rita1239	0.21	0.416	
771	Kalkatungu	kalk1246	0.21	0.418	
363	Yir Yoront	yiry1245	0.21	0.418	
38	Djabugay	dyaa1242	0.20	0.422	
656	Kuugu Ya'u	kuuk1238	0.19	0.425	
901	Guugu Yimidhirr	gugu1255	0.15	0.440	
99	Bidyara	bidy1243	0.15	0.441	
915	Alawa	alaw1244	0.10	0.462	
734	Kukatja	kuka1246	0.09	0.464	
800	Guwamu	guwa1243	0.08	0.467	
540	Ngiyambaa	wang1291	0.08	0.467	
62	Dhay'yi	dhal1246	0.07	0.473	
752	Kija	kitj1240	0.04	0.483	
934	Urningangg	urni1239	0.02	0.490	
105	Bardi	bard1255	0.01	0.496	
1032	Kuku Yalanji	kuku1273	0.00	0.499	
265	Wardaman	ward1246	-0.02	0.507	
962	Gunya	guny1241	-0.02	0.508	
252	Warlmanpa	warl1255	-0.03	0.513	
910	Nungali	nung1291	-0.03	0.513	
12	Erre	erre1238	-0.04	0.517	
946	Kurtjar	gurd1238	-0.10	0.539	
1018	Djapu	djap1238	-0.11	0.543	
1012	Warrgamay	warr1255	-0.13	0.552	
427	Yidiny	yidi1250	-0.13	0.553	
170	Wik-Ngathan	wikn1245	-0.14	0.554	
546	Yanyuwa	yany1243	-0.15	0.558	
925	Larrakia	lara1258	-0.15	0.560	
821	Gumbaynggir	kumb1268	-0.16	0.563	
598	Yandruwandha	yand1253	-0.18	0.571	
911	Nyawaygi	nyaw1247	-0.18	0.572	
923	Matngele	madn1237	-0.21	0.582	
978	Wotjobaluk	wotj1234	-0.25	0.600	
926	Limilngan	nucl1327	-0.31	0.621	

Lex ID	Language	Glottolog	R	p	Signif.
495	Nyikina	nyig1240	-0.32	0.624	
621	Mengerrdji	mang1382	-0.32	0.624	
928	Bakanh	paka1251	-0.34	0.631	
94	Biri	biri1256	-0.34	0.631	
744	Koko Bera	gugu1254	-0.35	0.637	
996	Emmi	amii1238	-0.36	0.642	
1026	Gidabal	gida1240	-0.37	0.643	
1002	Yintyingka	ayab1239	-0.37	0.645	
1025	Waalubal	band1358	-0.45	0.675	
650	Linngithigh	leni1238	-0.52	0.700	
305	Western Wakaya	waga1260	-0.72	0.764	
30	Duungidjawu	duun1241	-0.75	0.772	
737	Kukatj	guga1239	-0.75	0.773	
964	Olkol	ulku1238	-0.77	0.780	
965	Oykangand	oyka1239	-0.90	0.817	

Table S5.11. Comparing power law and exponential distributions with x_{min} using Vuong's likelihood ratio test.

Part B. Using x_{min} from the exponential fit. $R > 0$ favours exponential.

* $p < 0.05$ after Bonferroni correction. ** $p < 0.01$ after Bonferroni correction.

Lex ID	Language	Glottolog	R	p	Signif.
363	Yir Yoront	yiry1245	4.78	0.000	**
918	Atampaya	atam1239	4.40	0.000	**
996	Emmi	amii1238	4.34	0.000	**
117	Wirangu	wira1265	4.13	0.000	**
618	Mirniny	mirn1243	3.82	0.000	*
1031	Ngadjunmaya	ngad1258	3.74	0.000	*
228	Warriyangga	wari1262	3.69	0.000	*
99	Bidyara	bidy1243	3.66	0.000	*
920	Yadhaykenu	yadh1237	3.59	0.000	*
1024	Ngardily	west2437	3.45	0.000	*
642	Mangala	mang1383	3.39	0.000	
982	Nhirrpi	nhir1234	3.37	0.000	
305	Western Wakaya	waga1260	3.35	0.000	
917	Nyamal	nyam1271	3.34	0.000	
800	Guwamu	guwa1243	3.27	0.001	
493	Nyiyaparli	nija1241	3.24	0.001	
762	Kariyarra	kari1304	3.24	0.001	
852	Gurindji	guri1247	3.22	0.001	
760	Kartujarra	kart1247	3.21	0.001	
845	Iwaidja	iwai1244	3.17	0.001	
934	Urningangg	urni1239	3.14	0.001	
856	Patjtjamalh	wadj1254	3.14	0.001	
77	Dhangu	dhan1270	3.12	0.001	
1003	Murrinh-patha	murr1259	3.09	0.001	
94	Biri	biri1256	3.09	0.001	
737	Kukatj	guga1239	3.08	0.001	
12	Erre	erre1238	3.07	0.001	
118	Badimaya	badi1246	3.04	0.001	
941	Wambaya	nucl1328	3.02	0.001	
650	Linngithigh	leni1238	2.96	0.002	
5	Gangulu	gang1268	2.95	0.002	
563	Ngarla	ngar1296	2.95	0.002	
1016	Tharrkari	dhar1247	2.94	0.002	
838	Gooniyandi	goon1238	2.93	0.002	
945	Wiri	biri1256	2.91	0.002	
857	Amurdak	amar1271	2.90	0.002	
1009	Gamilaraay	gami1243	2.86	0.002	
606	Mudburra	mudb1240	2.86	0.002	
905	Malkana	malg1242	2.85	0.002	
697	Kurrama	kurr1243	2.83	0.002	
1030	Putijarra	pudi1238	2.81	0.002	
734	Kukatja	kuka1246	2.81	0.002	
964	Olkol	ulku1238	2.71	0.003	
546	Yanyuwa	yany1243	2.68	0.004	
252	Warlmanpa	warl1255	2.67	0.004	
1042	Thaayorre	thay1249	2.65	0.004	
443	Payungu	bayu1240	2.65	0.004	

Lex ID	Language	Glottolog	R	p	Signif.
269	Wangkumara	wong1246	2.64	0.004	
740	Kugu Nganhcara	wikn1246	2.62	0.004	
1021	Watjarri	waja1257	2.55	0.005	
1029	Martuthunira	mart1255	2.55	0.005	
1023	Wubuy	nung1290	2.50	0.006	
1040	Ngarinyin	ngar1284	2.48	0.007	
1001	Ngandi	ngan1295	2.46	0.007	
31	Djinang	djin1253	2.43	0.008	
926	Limilngan	nucl1327	2.40	0.008	
1002	Yintyingka	ayab1239	2.37	0.009	
1007	Yuwaliyaay	yuwa1243	2.35	0.009	
787	Jawoyn	djau1244	2.35	0.009	
1018	Djapu	djap1238	2.31	0.010	
919	Angkamuthi	angg1238	2.31	0.011	
377	Umpila	umpi1239	2.29	0.011	
965	Oykangand	oyka1239	2.29	0.011	
204	Wemba Wemba	wemb1241	2.25	0.012	
91	Bularnu	bula1255	2.25	0.012	
853	Lardil	lard1243	2.23	0.013	
790	Jaru	jaru1254	2.18	0.015	
417	Yindjibarndi	yind1247	2.17	0.015	
957	Ngaanyatjarra	ngaa1240	2.14	0.016	
242	Warlpiri	warl1254	2.12	0.017	
778	Jiwarli	djiw1241	2.06	0.020	
952	Ngawun	ngaw1240	2.01	0.022	
968	Ogh Angkula	ikar1243	1.98	0.024	
412	Ritharrngu	rita1239	1.97	0.025	
771	Kalkatungu	kalk1246	1.95	0.025	
1011	Yorta Yorta	yort1237	1.91	0.028	
30	Duungidjawu	duun1241	1.91	0.028	
939	Unggumi	ungg1243	1.91	0.028	
835	Gugu Badhun	gugu1253	1.90	0.029	
979	Mawng	maun1240	1.89	0.029	
554	Ngarluma	ngar1287	1.88	0.030	
89	Bunuba	buna1275	1.85	0.032	
495	Nyikina	nyig1240	1.85	0.032	
656	Kuugu Ya'u	kuuk1238	1.82	0.034	
288	Wangkatja	pini1245	1.80	0.036	
631	Yalarnnga	yala1262	1.78	0.038	
925	Larrakia	lara1258	1.75	0.040	
1043	Mbabaram	mbab1239	1.74	0.041	
611	Anguthimri	angu1242	1.74	0.041	
767	Karajarri	kara1476	1.73	0.042	
200	Western Arrernte	west2441	1.71	0.044	
922	Nakara	naka1260	1.70	0.044	
807	Gupapuyngu	gupa1247	1.70	0.044	
911	Nyawaygi	nyaw1247	1.70	0.045	
929	Wanyjirra	wany1244	1.68	0.046	
63	Dharumbal	dhar1248	1.68	0.046	
923	Matngele	madn1237	1.59	0.055	
427	Yidiny	yidi1250	1.58	0.057	
13	Dyirbal	dyir1250	1.58	0.057	

Lex ID	Language	Glottolog	R	p	Signif.
863	Marra	mara1385	1.56	0.059	
927	Tiwi	tiwi1244	1.55	0.060	
928	Bakanh	paka1251	1.47	0.070	
1006	Central Arrernte	mpar1238	1.47	0.071	
1012	Warrgamay	warr1255	1.47	0.071	
940	Yawijibaya	yawi1239	1.46	0.073	
265	Wardaman	ward1246	1.41	0.080	
1026	Gidabal	gida1240	1.40	0.080	
921	Gurr-Goni	gura1252	1.39	0.082	
958	Margany	marg1253	1.32	0.093	
915	Alawa	alaw1244	1.31	0.095	
978	Wotjobaluk	wotj1234	1.30	0.096	
930	Purduna	burd1238	1.26	0.104	
1025	Waalubal	band1358	1.24	0.107	
540	Ngiyambaa	wang1291	1.23	0.109	
1019	Walmajarri	walm1241	1.23	0.109	
962	Gunya	guny1241	1.22	0.111	
977	Warndarrang	wand1263	1.21	0.113	
972	Ogh Unyjan	kawa1290	1.20	0.115	
621	Mengerrdji	mang1382	1.17	0.121	
946	Kurtjar	gurd1238	1.11	0.132	
565	Ngarinyman	ngar1235	1.08	0.140	
237	Warluwarra	warl1256	1.04	0.150	
62	Dhay'yi	dhal1246	1.03	0.151	
985	Yaygir	yayg1236	1.03	0.151	
105	Bardi	bard1255	1.01	0.156	
935	Rembarrnga	remb1249	1.00	0.159	
744	Koko Bera	gugu1254	0.98	0.163	
38	Djabugay	dyaa1242	0.98	0.164	
901	Guugu Yimidhirr	gugu1255	0.97	0.166	
848	Wagiman	wage1238	0.91	0.182	
752	Kija	kitj1240	0.91	0.182	
849	Waanyi	wany1247	0.86	0.195	
113	Yulparija	yulp1239	0.85	0.197	
81	Dalabon	ngal1292	0.82	0.206	
592	Muruwari	muru1266	0.81	0.209	
851	Bilinarra	bili1250	0.77	0.220	
841	Worrorra	woro1258	0.72	0.235	
821	Gumbaynggir	kumb1268	0.71	0.238	
598	Yandruwandha	yand1253	0.70	0.241	
400	Thalanyji	dhal1245	0.64	0.261	
847	Ngalakgan	ngal1293	0.60	0.276	
462	Panyjima	pany1241	0.59	0.277	
620	Miriwoong	miri1266	0.58	0.280	
232	Warnman	wanm1242	0.58	0.282	
967	Thaynakwithi	tyan1235	0.54	0.293	
645	Malyangapa	maly1234	0.53	0.297	
170	Wik-Ngathan	wikn1245	0.51	0.304	
914	Yinhawangka	yinh1234	0.47	0.320	
433	Pitta Pitta	pitt1247	0.44	0.331	
966	Wik Mungkan	wikm1247	0.39	0.347	
949	Kok Nar	kokn1236	0.38	0.352	

**Lex ID**	**Language**	**Glottolog**	R	p	**Signif.**
507	Nyangumarta	nyan1301	0.35	0.363	
519	Nukunu	nugu1241	0.33	0.370	
983	Nhangu	yann1237	0.30	0.384	
910	Nungali	nung1291	0.29	0.385	
1032	Kuku Yalanji	kuku1273	0.27	0.394	
534	Nhanda	nhan1238	0.26	0.398	
1008	Yuwaalaraay	yuwa1242	0.25	0.401	
475	Southern Paakintyi	darl1243	0.25	0.403	
943	Pintupi	pint1250	0.24	0.403	
206	Wayilwan	wayi1238	0.14	0.443	
866	Thirarri	dira1238	0.12	0.452	
865	Diyari	dier1241	0.08	0.467	
162	Adnyamathanha	adny1235	0.03	0.489	
85	Burarra	bura1267	-0.23	0.591	
101	Butchulla	baty1234	-0.63	0.735	

Table S5.12. Comparing power law and lognormal distributions with x_{min} using Vuong's likelihood ratio test.

Part A. Using x_{min} from the power law fit. $R > 0$ favours power law.

* $p < 0.05$ after Bonferroni correction. ** $p < 0.01$ after Bonferroni correction.

Lex ID	Language	Glottolog	R	p	Signif.
200	Western Arrernte	west2441	0.66	0.253	
1007	Yuwaliyaay	yuwa1243	0.60	0.274	
1042	Thaayorre	thay1249	0.42	0.338	
1016	Tharrkari	dhar1247	0.36	0.358	
847	Ngalakgan	ngal1293	0.30	0.383	
905	Malkana	malg1242	0.29	0.387	
982	Nhirrpi	nhir1234	0.25	0.400	
968	Ogh Angkula	ikar1243	0.24	0.405	
363	Yir Yoront	yiry1245	0.22	0.415	
1040	Ngarinyin	ngar1284	0.18	0.430	
853	Lardil	lard1243	0.17	0.431	
1009	Gamilaraay	gami1243	0.17	0.431	
935	Rembarrnga	remb1249	0.16	0.435	
790	Jaru	jaru1254	0.12	0.453	
1031	Ngadjunmaya	ngad1258	0.09	0.466	
1008	Yuwaalaraay	yuwa1242	0.07	0.472	
985	Yaygir	yayg1236	0.05	0.479	
620	Miriwoong	miri1266	0.05	0.480	
787	Jawoyn	djau1244	0.03	0.488	
762	Kariyarra	kari1304	0.02	0.492	
89	Bunuba	buna1275	0.01	0.496	
945	Wiri	biri1256	0.00	0.499	
162	Adnyamathanha	adny1235	0.00	0.500	
206	Wayilwan	wayi1238	-0.02	0.507	
118	Badimaya	badi1246	-0.03	0.512	
1043	Mbabaram	mbab1239	-0.03	0.512	
13	Dyirbal	dyir1250	-0.05	0.522	
841	Worrorra	woro1258	-0.06	0.524	
940	Yawijibaya	yawi1239	-0.07	0.527	
918	Atampaya	atam1239	-0.08	0.531	
1006	Central Arrernte	mpar1238	-0.09	0.534	
1011	Yorta Yorta	yort1237	-0.10	0.540	
540	Ngiyambaa	wang1291	-0.12	0.547	
412	Ritharrngu	rita1239	-0.12	0.547	
606	Mudburra	mudb1240	-0.12	0.547	
618	Mirniny	mirn1243	-0.12	0.549	
856	Patjtjamalh	wadj1254	-0.12	0.549	
972	Ogh Unyjan	kawa1290	-0.13	0.552	
930	Purduna	burd1238	-0.13	0.552	
949	Kok Nar	kokn1236	-0.13	0.553	
1001	Ngandi	ngan1295	-0.13	0.553	
554	Ngarluma	ngar1287	-0.14	0.555	
642	Mangala	mang1383	-0.15	0.559	
939	Unggumi	ungg1243	-0.16	0.564	
977	Warndarrang	wand1263	-0.16	0.565	
917	Nyamal	nyam1271	-0.18	0.570	
849	Waanyi	wany1247	-0.18	0.572	

**Lex ID**	**Language**	**Glottolog**	*R*	*p*	**Signif.**
919	Angkamuthi	angg1238	-0.18	0.572	
920	Yadhaykenu	yadh1237	-0.18	0.573	
1029	Martuthunira	mart1255	-0.18	0.573	
845	Iwaidja	iwai1244	-0.19	0.576	
534	Nhanda	nhan1238	-0.19	0.577	
5	Gangulu	gang1268	-0.20	0.578	
958	Margany	marg1253	-0.20	0.578	
1003	Murrinh-patha	murr1259	-0.21	0.582	
767	Karajarri	kara1476	-0.21	0.582	
631	Yalarnnga	yala1262	-0.21	0.583	
921	Gurr-Goni	gura1252	-0.21	0.583	
929	Wanyjirra	wany1244	-0.21	0.583	
427	Yidiny	yidi1250	-0.23	0.590	
443	Payungu	bayu1240	-0.23	0.592	
400	Thalanyji	dhal1245	-0.23	0.593	
910	Nungali	nung1291	-0.24	0.593	
101	Butchulla	baty1234	-0.24	0.594	
1019	Walmajarri	walm1241	-0.24	0.595	
63	Dharumbal	dhar1248	-0.24	0.596	
778	Jiwarli	djiw1241	-0.24	0.596	
563	Ngarla	ngar1296	-0.24	0.596	
38	Djabugay	dyaa1242	-0.25	0.597	
911	Nyawaygi	nyaw1247	-0.25	0.601	
493	Nyiyaparli	nija1241	-0.26	0.602	
852	Gurindji	guri1247	-0.26	0.604	
941	Wambaya	nucl1328	-0.27	0.608	
204	Wemba Wemba	wemb1241	-0.28	0.609	
934	Urningangg	urni1239	-0.28	0.610	
967	Thaynakwithi	tyan1235	-0.28	0.612	
740	Kugu Nganhcara	wikn1246	-0.29	0.613	
857	Amurdak	amar1271	-0.30	0.617	
863	Marra	mara1385	-0.30	0.617	
462	Panyjima	pany1241	-0.30	0.619	
835	Gugu Badhun	gugu1253	-0.31	0.621	
1021	Watjarri	waja1257	-0.32	0.625	
923	Matngele	madn1237	-0.32	0.625	
288	Wangkatja	pini1245	-0.32	0.627	
228	Warriyangga	wari1262	-0.32	0.627	
752	Kija	kitj1240	-0.33	0.629	
866	Thirarri	dira1238	-0.33	0.630	
914	Yinhawangka	yinh1234	-0.33	0.630	
865	Diyari	dier1241	-0.34	0.633	
952	Ngawun	ngaw1240	-0.35	0.636	
979	Mawng	maun1240	-0.35	0.637	
1030	Putijarra	pudi1238	-0.35	0.639	
252	Warlmanpa	warl1255	-0.36	0.640	
760	Kartujarra	kart1247	-0.37	0.644	
170	Wik-Ngathan	wikn1245	-0.37	0.644	
519	Nukunu	nugu1241	-0.37	0.644	
565	Ngarinyman	ngar1235	-0.37	0.645	
848	Wagiman	wage1238	-0.38	0.647	
12	Erre	erre1238	-0.38	0.648	

Lex ID	Language	Glottolog	R	p	Signif.
377	Umpila	umpi1239	-0.38	0.648	
113	Yulparija	yulp1239	-0.38	0.648	
1024	Ngardily	west2437	-0.38	0.649	
957	Ngaanyatjarra	ngaa1240	-0.39	0.651	
925	Larrakia	lara1258	-0.39	0.652	
946	Kurtjar	gurd1238	-0.39	0.653	
77	Dhangu	dhan1270	-0.39	0.654	
915	Alawa	alaw1244	-0.40	0.655	
117	Wirangu	wira1265	-0.40	0.656	
943	Pintupi	pint1250	-0.40	0.656	
417	Yindjibarndi	yind1247	-0.40	0.656	
744	Koko Bera	gugu1254	-0.41	0.658	
237	Warluwarra	warl1256	-0.41	0.661	
821	Gumbaynggir	kumb1268	-0.42	0.662	
507	Nyangumarta	nyan1301	-0.42	0.664	
433	Pitta Pitta	pitt1247	-0.43	0.667	
734	Kukatja	kuka1246	-0.43	0.668	
978	Wotjobaluk	wotj1234	-0.44	0.672	
901	Guugu Yimidhirr	gugu1255	-0.45	0.675	
737	Kukatj	guga1239	-0.46	0.678	
656	Kuugu Ya'u	kuuk1238	-0.46	0.678	
1023	Wubuy	nung1290	-0.46	0.679	
85	Burarra	bura1267	-0.46	0.679	
983	Nhangu	yann1237	-0.47	0.680	
242	Warlpiri	warl1254	-0.48	0.684	
1026	Gidabal	gida1240	-0.48	0.685	
265	Wardaman	ward1246	-0.49	0.689	
305	Western Wakaya	waga1260	-0.50	0.690	
927	Tiwi	tiwi1244	-0.50	0.692	
81	Dalabon	ngal1292	-0.50	0.693	
592	Muruwari	muru1266	-0.51	0.695	
1012	Warrgamay	warr1255	-0.51	0.695	
1025	Waalubal	band1358	-0.52	0.697	
598	Yandruwandha	yand1253	-0.52	0.697	
621	Mengerrdji	mang1382	-0.52	0.699	
1032	Kuku Yalanji	kuku1273	-0.53	0.701	
838	Gooniyandi	goon1238	-0.53	0.703	
91	Bularnu	bula1255	-0.54	0.706	
922	Nakara	naka1260	-0.55	0.708	
31	Djinang	djin1253	-0.56	0.711	
851	Bilinarra	bili1250	-0.56	0.712	
800	Guwamu	guwa1243	-0.57	0.717	
232	Warnman	wanm1242	-0.58	0.717	
611	Anguthimri	angu1242	-0.58	0.719	
99	Bidyara	bidy1243	-0.59	0.724	
966	Wik Mungkan	wikm1247	-0.60	0.726	
62	Dhay'yi	dhal1246	-0.60	0.727	
94	Biri	biri1256	-0.65	0.741	
645	Malyangapa	maly1234	-0.65	0.741	
697	Kurrama	kurr1243	-0.66	0.745	
1018	Djapu	djap1238	-0.68	0.751	
928	Bakanh	paka1251	-0.69	0.755	

Lex ID	Language	Glottolog	R	p	Signif.
105	Bardi	bard1255	-0.74	0.771	
1002	Yintyingka	ayab1239	-0.75	0.774	
495	Nyikina	nyig1240	-0.75	0.774	
30	Duungidjawu	duun1241	-0.76	0.776	
996	Emmi	amii1238	-0.77	0.780	
807	Gupapuyngu	gupa1247	-0.79	0.785	
926	Limilngan	nucl1327	-0.82	0.794	
269	Wangkumara	wong1246	-0.85	0.803	
546	Yanyuwa	yany1243	-0.86	0.806	
771	Kalkatungu	kalk1246	-0.89	0.814	
964	Olkol	ulku1238	-0.91	0.820	
650	Linngithigh	leni1238	-0.93	0.824	
965	Oykangand	oyka1239	-0.93	0.824	
475	Southern Paakintyi	darl1243	-0.95	0.828	
962	Gunya	guny1241	-1.42	0.923	

Table S5.12. Comparing power law and lognormal distributions with x_{min} using Vuong's likelihood ratio test.

Part B. Using x_{min} from the lognormal fit. $R > 0$ favours lognormal.

* $p < 0.05$ after Bonferroni correction. ** $p < 0.01$ after Bonferroni correction.

Lex ID	Language	Glottolog	R	p	Signif.
920	Yadhaykenu	yadh1237	3.41	0.000	
433	Pitta Pitta	pitt1247	3.33	0.000	
77	Dhangu	dhan1270	3.09	0.001	
982	Nhirrpi	nhir1234	2.96	0.002	
935	Rembarrnga	remb1249	2.94	0.002	
631	Yalarnnga	yala1262	2.90	0.002	
996	Emmi	amii1238	2.89	0.002	
305	Western Wakaya	waga1260	2.80	0.003	
105	Bardi	bard1255	2.79	0.003	
901	Guugu Yimidhirr	gugu1255	2.79	0.003	
1029	Martuthunira	mart1255	2.77	0.003	
1023	Wubuy	nung1290	2.74	0.003	
848	Wagiman	wage1238	2.69	0.004	
546	Yanyuwa	yany1243	2.68	0.004	
968	Ogh Angkula	ikar1243	2.65	0.004	
760	Kartujarra	kart1247	2.64	0.004	
946	Kurtjar	gurd1238	2.62	0.004	
598	Yandruwandha	yand1253	2.61	0.005	
1018	Djapu	djap1238	2.59	0.005	
650	Linngithigh	leni1238	2.56	0.005	
1042	Thaayorre	thay1249	2.56	0.005	
412	Ritharrngu	rita1239	2.53	0.006	
30	Duungidjawu	duun1241	2.52	0.006	
983	Nhangu	yann1237	2.49	0.006	
853	Lardil	lard1243	2.49	0.006	
534	Nhanda	nhan1238	2.37	0.009	
204	Wemba Wemba	wemb1241	2.33	0.010	
618	Mirniny	mirn1243	2.33	0.010	
926	Limilngan	nucl1327	2.32	0.010	
62	Dhay'yi	dhal1246	2.29	0.011	
1016	Tharrkari	dhar1247	2.28	0.011	
1043	Mbabaram	mbab1239	2.26	0.012	
1011	Yorta Yorta	yort1237	2.25	0.012	
1019	Walmajarri	walm1241	2.25	0.012	
852	Gurindji	guri1247	2.25	0.012	
930	Purduna	burd1238	2.22	0.013	
237	Warluwarra	warl1256	2.21	0.014	
979	Mawng	maun1240	2.19	0.014	
967	Thaynakwithi	tyan1235	2.18	0.015	
847	Ngalakgan	ngal1293	2.17	0.015	
620	Miriwoong	miri1266	2.16	0.015	
918	Atampaya	atam1239	2.15	0.016	
377	Umpila	umpi1239	2.15	0.016	
964	Olkol	ulku1238	2.14	0.016	
81	Dalabon	ngal1292	2.13	0.016	
800	Guwamu	guwa1243	2.12	0.017	
1009	Gamilaraay	gami1243	2.12	0.017	

Lex ID	Language	Glottolog	R	p	Signif.
118	Badimaya	badi1246	2.11	0.018	
965	Oykangand	oyka1239	2.10	0.018	
63	Dharumbal	dhar1248	2.07	0.019	
925	Larrakia	lara1258	2.04	0.021	
966	Wik Mungkan	wikm1247	2.04	0.021	
923	Matngele	madn1237	1.96	0.025	
928	Bakanh	paka1251	1.95	0.026	
1007	Yuwaliyaay	yuwa1243	1.94	0.026	
265	Wardaman	ward1246	1.93	0.027	
269	Wangkumara	wong1246	1.91	0.028	
790	Jaru	jaru1254	1.90	0.029	
495	Nyikina	nyig1240	1.89	0.029	
363	Yir Yoront	yiry1245	1.88	0.030	
744	Koko Bera	gugu1254	1.84	0.033	
856	Patjtjamalh	wadj1254	1.82	0.035	
978	Wotjobaluk	wotj1234	1.72	0.042	
962	Gunya	guny1241	1.71	0.043	
621	Mengerrdji	mang1382	1.70	0.045	
162	Adnyamathanha	adny1235	1.67	0.047	
835	Gugu Badhun	gugu1253	1.65	0.049	
697	Kurrama	kurr1243	1.65	0.050	
1032	Kuku Yalanji	kuku1273	1.65	0.050	
89	Bunuba	buna1275	1.62	0.053	
787	Jawoyn	djau1244	1.59	0.056	
1025	Waalubal	band1358	1.58	0.057	
1012	Warrgamay	warr1255	1.57	0.058	
921	Gurr-Goni	gura1252	1.57	0.058	
1040	Ngarinyin	ngar1284	1.57	0.058	
927	Tiwi	tiwi1244	1.56	0.060	
200	Western Arrernte	west2441	1.56	0.060	
934	Urningangg	urni1239	1.55	0.061	
170	Wik-Ngathan	wikn1245	1.52	0.064	
565	Ngarinyman	ngar1235	1.51	0.065	
1026	Gidabal	gida1240	1.51	0.066	
493	Nyiyaparli	nija1241	1.50	0.067	
821	Gumbaynggir	kumb1268	1.49	0.068	
1002	Yintyingka	ayab1239	1.48	0.069	
841	Worrorra	woro1258	1.48	0.069	
5	Gangulu	gang1268	1.46	0.072	
94	Biri	biri1256	1.43	0.076	
911	Nyawaygi	nyaw1247	1.43	0.076	
13	Dyirbal	dyir1250	1.41	0.080	
737	Kukatj	guga1239	1.40	0.081	
771	Kalkatungu	kalk1246	1.39	0.082	
1001	Ngandi	ngan1295	1.37	0.086	
915	Alawa	alaw1244	1.37	0.086	
427	Yidiny	yidi1250	1.35	0.089	
866	Thirarri	dira1238	1.35	0.089	
865	Diyari	dier1241	1.32	0.094	
1008	Yuwaalaraay	yuwa1242	1.29	0.099	
851	Bilinarra	bili1250	1.27	0.103	
540	Ngiyambaa	wang1291	1.23	0.109	

Lex ID	Language	Glottolog	R	p	Signif.
857	Amurdak	amar1271	1.22	0.112	
117	Wirangu	wira1265	1.18	0.118	
252	Warlmanpa	warl1255	1.18	0.119	
941	Wambaya	nucl1328	1.13	0.129	
1031	Ngadjunmaya	ngad1258	1.10	0.135	
475	Southern Paakintyi	darl1243	1.10	0.136	
734	Kukatja	kuka1246	1.00	0.160	
752	Kija	kitj1240	0.97	0.166	
38	Djabugay	dyaa1242	0.94	0.175	
767	Karajarri	kara1476	0.91	0.182	
952	Ngawun	ngaw1240	0.90	0.184	
922	Nakara	naka1260	0.87	0.192	
113	Yulparija	yulp1239	0.86	0.194	
417	Yindjibarndi	yind1247	0.81	0.209	
645	Malyangapa	maly1234	0.81	0.210	
945	Wiri	biri1256	0.80	0.212	
592	Muruwari	muru1266	0.80	0.212	
807	Gupapuyngu	gupa1247	0.79	0.215	
31	Djinang	djin1253	0.78	0.218	
943	Pintupi	pint1250	0.75	0.227	
85	Burarra	bura1267	0.74	0.230	
462	Panyjima	pany1241	0.70	0.241	
232	Warnman	wanm1242	0.70	0.242	
242	Warlpiri	warl1254	0.68	0.248	
656	Kuugu Ya'u	kuuk1238	0.67	0.250	
910	Nungali	nung1291	0.66	0.255	
949	Kok Nar	kokn1236	0.64	0.260	
914	Yinhawangka	yinh1234	0.63	0.263	
606	Mudburra	mudb1240	0.62	0.268	
519	Nukunu	nugu1241	0.61	0.271	
563	Ngarla	ngar1296	0.60	0.273	
99	Bidyara	bidy1243	0.59	0.276	
611	Anguthimri	angu1242	0.58	0.281	
917	Nyamal	nyam1271	0.58	0.282	
228	Warriyangga	wari1262	0.57	0.283	
91	Bularnu	bula1255	0.54	0.294	
838	Gooniyandi	goon1238	0.53	0.297	
1021	Watjarri	waja1257	0.52	0.303	
554	Ngarluma	ngar1287	0.51	0.306	
206	Wayilwan	wayi1238	0.47	0.321	
863	Marra	mara1385	0.46	0.322	
929	Wanyjirra	wany1244	0.46	0.324	
905	Malkana	malg1242	0.45	0.325	
958	Margany	marg1253	0.44	0.330	
507	Nyangumarta	nyan1301	0.42	0.336	
957	Ngaanyatjarra	ngaa1240	0.39	0.349	
939	Unggumi	ungg1243	0.39	0.350	
1024	Ngardily	west2437	0.38	0.351	
12	Erre	erre1238	0.38	0.352	
972	Ogh Unyjan	kawa1290	0.37	0.355	
443	Payungu	bayu1240	0.36	0.360	
1003	Murrinh-patha	murr1259	0.36	0.360	

Lex ID	Language	Glottolog	R	p	Signif.
1030	Putijarra	pudi1238	0.35	0.361	
288	Wangkatja	pini1245	0.32	0.373	
642	Mangala	mang1383	0.31	0.377	
740	Kugu Nganhcara	wikn1246	0.29	0.387	
778	Jiwarli	djiw1241	0.28	0.390	
101	Butchulla	baty1234	0.24	0.406	
400	Thalanyji	dhal1245	0.23	0.407	
845	Iwaidja	iwai1244	0.19	0.424	
919	Angkamuthi	angg1238	0.18	0.428	
849	Waanyi	wany1247	0.18	0.428	
1006	Central Arrernte	mpar1238	0.17	0.431	
977	Warndarrang	wand1263	0.16	0.435	
762	Kariyarra	kari1304	0.08	0.470	
940	Yawijibaya	yawi1239	0.07	0.473	
985	Yaygir	yayg1236	-0.06	0.522	

References

Bowe, Heather & Stephen Morey. 1999. *The Yorta Yorta (Bangerang) language of the Murray Goulburn including Yabula Yabula* (Pacific Linguistics Series C 154). Canberra: Pacific Linguistics. 286 pp.

Breen, Gavan. 1976. An introduction to Gog-Nar. In Peter Sutton (ed.), *Languages of Cape York*, vol. 6 (Australian Aboriginal Studies: Research and Regional Studies), 243–259. Canberra: Australian Institute of Aboriginal Studies.

Breen, Gavan. 1978. Bularnu phonology and grammar.

Breen, Gavan. 1981a. Margany and Gunya. In R. M. W. Dixon & Barry Blake (eds.), *Handbook of Australian languages*, vol. 2, 275–394. Amsterdam: John Benjamins.

Chang, Winston et al. 2018. *shiny: Web Application Framework for R.* R package version 1.2.0. https://CRAN.R-project.org/package=shiny.

Crowley, Terry. 1983. Uradhi. In R. M. W. Dixon & Barry J. Blake (eds.), *Handbook of Australian languages*, vol. 3, 5 vols., 307–428. Amsterdam: John Benjamins.

Harvey, Mark. 2001. *A grammar of Limilngan: A language of the Mary River region, Northern Territory, Australia* (Pacific Linguistics 516). Canberra: Pacific Linguistics. 209 pp. https://doi.org/10.15144/PL-516.

McEntee, John & Pearl McKenzie. 1992. *Adna-mat-na English dictionary.* Adelaide: the authors. 125 pp.

Moran, Steven & Daniel McCloy (eds.). 2019. *PHOIBLE 2.0.* Jena: Max Planck Institute for the Science of Human History. https://phoible.org/.

O'Grady, Geoffrey N., C. F. Voegelin & F. M. Voegelin. 1966. Languages of the World: Indo-Pacific fascicle 6. *Anthropological Linguistics* 8(2). 1–199.

Round, Erich R. 2019. Phonemic inventories of Australia [Database of 392 languages]. In Steven Moran & Daniel McCloy (eds.), *PHOIBLE 2.0.* Jena: Max Planck Institute for the Science of Human History.

Schebeck, B. 1974. *Texts on the social system of the Atynyamatana people with grammatical notes* (Pacific Linguistics Series D 21). Canberra: Pacific Linguistics. https://doi.org/10.15144/PL-D21.

Sommer, Bruce A. N.d.(b). Koko Narr. Fryer Library Bruce Sommer Collection. UQFL476_b10f03_64, UQFL476_b10f03_65. Brisbane.

Wangka Maya Pilbara Aboriginal Language Centre & Australian Institute of Aboriginal and Torres Strait Islander Studies. 2004. *Putijarra-English wordlist, English-Putijarra finder topical wordlist &sketch morphology.* South Hedland, WA, Australia: Wangka Maya Pilbara Aboriginal Language Centre. 118 pp.
